# Integrated Whole-Transcriptome Analysis to Elucidate the Core Regulatory Network of circRNA Involved in Ovarian Development and Reproductive Capacity Differences in Sheep: circRNA2058-miR-9226-5p-*MET* Axis

**DOI:** 10.3390/ani15213077

**Published:** 2025-10-23

**Authors:** Bo Gu, Anqi Wang, Xinmiao Yu, Ying Li, Yao Cong, Huaizhi Jiang

**Affiliations:** 1 College of Life Science, Jilin Normal University, Siping 136000, China; 17743110597@163.com (B.G.);; 2 College of Animal Science and Technology, Jilin Agricultural University, Changchun 130118, China

**Keywords:** Ujumqin sheep, small-tailed Han sheep, circRNA-miRNA-mRNA, ovary

## Abstract

**Simple Summary:**

This study investigated the genetic regulation of ovarian development in sheep with high and low fecundity. Using whole-transcriptome sequencing of ovaries from different breeds and developmental stages, the researchers constructed competing endogenous RNA (ceRNA) networks. These networks revealed key interactions between circRNAs, miRNAs, and mRNAs. A central regulatory axis, circRNA2058-miR-9226-5p-*MET*, was identified and experimentally validated. This axis acts as an potential critical molecular switch controlling follicular development. The findings provide important insights into the molecular mechanisms behind sheep reproduction and offer a foundation for developing new strategies to improve reproductive efficiency in sheep.

**Abstract:**

(1) Background: This study aims to systematically identify key candidate genes and the regulatory networks governing ovarian development in sheep breeds with divergent fecundity. Focusing on elucidating the central regulatory roles of these factors during distinct ovarian developmental stages in highly prolific breeds, the research seeks to reveal the mechanism by which multilevel regulatory networks synergistically determine ewe reproductive capacity. (2) Methods: This study utilized the ovaries from the low-fecundity sheep breed Ujumqin sheep, the high-fecundity breed small-tailed Han sheep, and various developmental stages of small-tailed Han sheep as research subjects. Through whole-transcriptome sequencing analysis, differentially expressed mRNAs(DEGs) and non-coding RNAs (ncRNAs) were screened, and a ceRNA regulatory network was constructed and subjected to bioinformatic analysis. The dual-luciferase reporter gene detection system was employed to validate the targeting relationships within the obtained key circRNA-miRNA-mRNA networks. Finally, qRT-PCR was used to verify the accuracy of the sequencing results. (3) Results: Our analysis constructed two distinct ceRNA networks: one from different fecundity groups (116 DECs, 46 DEMs, 82 DEGs) and another from different ovarian stages (186 DECs, 143 DEMs, 338 DEGs). Functional enrichment revealed key reproduction-related pathways, including Mitogen-Activated Protein Kinase(MAPK), Janus Kinase-Signal Transducer and Activator of Transcription(JAK-STAT), and WNT signaling in the fecundity comparison, and MAPK, Ras, WNT, Hippo signaling in the developmental stage comparison. Integrated analysis identified a core circRNA-miRNA-mRNA network, pinpointing circRNA2058-miR-9226-5p-*MET* as a central regulatory axis. The dual-luciferase assay confirmed that circRNA2058 acts as a sponge for miR-9226-5p, thereby mediating *MET* expression. qRT-PCR validation of randomly selected RNAs confirmed the sequencing reliability. (4) Conclusions: this study deciphers a synergistic regulatory network and identifies, for the first time, the pivotal circRNA2058-miR-9226-5p-*MET* ceRNA axis as an potential critical molecular switch driving follicular dominance in sheep. This discovery provides a molecular foundation for targeting core regulators of ovine reproductive efficiency and offers significant insights for innovative strategies in enhancing sheep reproduction.

## 1. Introduction

Since the 1980s, market demand for mutton sheep has steadily increased, making the mutton sheep industry a key focus in the development of China’s sheep husbandry. To enhance the reproductive performance of sheep populations, China has prioritized the establishment of robust breeding systems, integrating both traditional and modern breeding techniques to develop and select new breeds. The small-tailed Han sheep, recognized for its year-round estrus and high prolificacy, is frequently utilized as a maternal breed [1]. However, despite China’s rich diversity of sheep genetic resources, high-fertility breeds remain scarce. The overwhelming majority of sheep breeds exhibit low fertility. The Ujumqin sheep, a representative low-fertility breed, is renowned as “one of the breeds producing the highest quality meat among livestock” in its region [2]. Its dairy products, meat, and wool are all of superior quality. Nevertheless, its application is significantly constrained by reproductive characteristics such as a long breeding cycle, low reproductive rate, and seasonal breeding patterns. Consequently, employing breeding strategies to improve the reproductive efficiency of low-fertility populations has become a strategic priority.

The ovary, as the central organ of the ewe reproductive system, exhibits a structure and functional status closely linked to fertility. Consequently, research into the reproductive regulatory functions of the ovary has consistently been a focal and challenging area in sheep husbandry. The advent of second-generation high-throughput parallel sequencing (Next-generation sequencing, NGS) technologies within molecular breeding has enabled transcriptome analyses based on RNA sequencing (RNA-seq). These analyses have revealed that the ovary harbors a complex transcriptional network, encompassing both coding and non-coding genes, which closely regulates key reproductive traits in sheep. This approach provides a crucial technical platform and research perspective for advancing our understanding of the molecular mechanisms through which the ovary modulates ovine reproductive performance. A comparative analysis of ovarian follicles from Boer goats and Macheng black goats revealed 37 DECs, suggesting that the expression levels of circRNAs may significantly influence ovarian follicle development and potentially contribute to the differences in reproductive rates between the two breeds [3]. During an in-depth analysis of Xiang pig ovarian samples collected during the estrus and diestrus stages, 156 DECs were identified. They proposed that these DECs may regulate gene expression during the porcine estrous cycle. Additionally, host genes of these DECs were predicted, revealing 432 miRNA targets. It was noted that each circRNA could contain one or more miRNA binding sites [4]. Previous research demonstrated that in bovine oocytes, ciRS-187 may upregulate BMPR2 expression by suppressing miR-187 [5]. Similarly, a novel circRNA, circSLC41A1, was found to enhance SRSF1 expression through competitive binding with miR-9820-5p, thereby regulating apoptosis in porcine follicular granulosa cells [6]. The study revealed that chi_circ_0031209 and chi_circ_0019448 play critical roles in reproduction during the luteal phase by modulating prolactin receptor expression in both high- and low-yield goats. Conversely, chi_circ_0014542 is involved in regulating *WNT5A* expression during the follicular phase, thereby modulating follicular development in mammals [7]. Comprehensive transcriptome sequencing of ovaries from sheep with divergent fertility revealed 180 DECs. We further demonstrated that novel_circ_0041512 modulates ovarian development in sheep by sponging miR-125b [8]. Previous research identified five key ceRNA interaction networks that regulate goat reproductive performance. Further analysis showed that these networks primarily influence ovarian function and reproductive performance by regulating biological processes such as germ cell development and oocyte development [9].

In summary, this study utilized ovarian tissues from the low-fecundity breed Ujumqin sheep (Ud group), the high-fecundity breed small-tailed Han sheep (Sad group), and small-tailed Han sheep at different developmental stages (Som—juvenile, Stm—pre-pubertal, Sad—sexually mature) as research subjects. With three biological replicates per group, we performed whole-transcriptome sequencing and subsequent bioinformatic analyses. The primary objectives were to identify key candidate genes and regulatory networks capable of concurrently modulating both breed-specific fecundity differences and developmental stage-dependent reproductive capacity in sheep. This research aims to elucidate the underlying multi-dimensional regulation mechanisms governing ovine reproductive efficiency. Furthermore, the findings are expected to provide a theoretical foundation and valuable references for improving reproductive performance in low-prolificacy sheep breeds and enhancing overall ewe fertility through targeted breeding programs.

## 2. Materials and Methods

### 2.1. Sample Collection

Ovarian tissues were collected from small-tailed Han sheep and Ujumqin sheep reared at Guofeng Livestock Breeding Farm (Songyuan, China). Three healthy one-month-old lambs, six-month-old lambs, and three adult multiparous small-tailed Han sheep ewes—all under identical feeding conditions—were selected, along with three adult multiparous Ujumqin sheep ewes. All adult ewes were confirmed to be in diestrus. Following slaughter, ovarian tissue blocks (3–5 cm^3^) were excised and immediately flash-frozen in liquid nitrogen for subsequent experiments. All methods were carried out in accordance with relevant guidelines set by the Ministry of Agriculture of the People’s Republic of China. All experimental protocols were approved by the Ethics Committee for Science and Technology of Jilin Normal University (KJLL20250403).

### 2.2. Total RNA Extraction and Library Construction

Total RNA was extracted from ovarian tissues using TRIzol reagent (Thermo Fisher Scienrific, Waltham, MA, USA), and its concentration and integrity were assessed using the NanoDrop2000 spectrophotometer (Thermo Fisher Scienrific, Waltham, MA, USA) and the Agilent Bioanalyzer (Agilent Technologies, Inc., Santa Clara, CA, USA). Strand-specific libraries (fr-firststrand) were constructed using the ribosomal RNA (rRNA) depletion method. After passing quality inspection, high-throughput sequencing was performed on the Illumina NovaSeq^TM^ 6000 platform (Illumina Inc., San Diego, CA, USA). Sequencing was conducted in paired-end mode with a read length of 150 bp (PE150), enabling simultaneous detection of both mRNA and circRNA expression levels. For miRNA analysis, small RNA (sRNA) sequencing libraries were prepared using the TruSeqTM Small RNA Sample Prep Kit (Illumina, San Diego, CA, USA). After library qualification, sequencing was carried out on the Illumina HiSeq 2000/2500 platform (Illumina Inc., San Diego, CA, USA), generating single-end 50 bp reads (SE50).

### 2.3. Sequencing Data Quality Control and Analysis

Raw sequencing data were processed using Cutadapt (v1.9) [10] to remove adapter sequences and low-quality reads, yielding high-quality clean data. Clean reads were then aligned to the reference genome using HISAT2 (v2.2.1) [11,12,13]. Gene-level read counts were quantified based on genomic coordinates defined in the genome annotation file. Using the alignment results, transcript reconstruction was conducted with StringTie (v2.1.6) [13,14,15], and the expression levels of all identified genes in each sample were subsequently quantified.

circRNA analysis diverges from mRNA analysis by focusing on reads that discordantly map to the reference genome v107. Reads failing to align linearly were analyzed to detect back-splice junctions (BSJs). circRNAs were identified using the intersection of predictions from both CIRCexplorer2 (v2.2.6) [16] and CIRI (v2.0.2) [17].

miRNA processing was performed using ACGT101-miR (v4.2). The pipeline included: 3′adapter trimming, removal of low-complexity sequences, and size selection (retaining reads 18–26 nt in length). Filtered reads were sequentially aligned against multiple RNA databases (including mRNA, RFam, and Repbase) to remove non-miRNA sequences. Final miRNA identification was achieved by mapping reads to precursor miRNAs and the reference genome.

### 2.4. Differential Expression Analysis and Target Gene Prediction

For the identification and quantification of circRNAs, we relied on back-splice junction (BSJ) reads. Differential expression analysis of circRNAs between groups was performed using edgeR (v3.22.5), with circRNAs satisfying |log_2_fc| ≥ 1 and *q* < 0.05 considered significantly differentially expressed. Given the inherent variation in circRNA expression across different tissues and developmental stages, we annotated the circRNAs based on their genomic locations and their associations with genes. Functional annotation of the circRNAs was primarily derived from the known functions of their parental genes.

For miRNAs, those with *p* < 0.05 were deemed differentially expressed. Target genes of these significantly differentially expressed miRNAs were predicted by intersecting the results from TargetScan (v5.0) and miRanda (v3.3a), applying stringent thresholds (TargetScan_score ≥ 50 and miRanda_Energy < −10).

### 2.5. Construction and Bioinformatics Analysis of the ceRNA Network

A crucial function of circRNAs is their ability to act as molecular sponges that sequester miRNAs. This competitive binding disrupts the regulatory activity of miRNAs on their target genes, ultimately altering protein expression levels. Within the ceRNA regulatory network, miRNAs occupy a central position. By calculating the Pearson correlation coefficient (r) and using thresholds of r < −0.4 and *p* < 0.05, negatively correlated circRNA-miRNA and miRNA-mRNA pairs, as well as positively correlated circRNA-mRNA pairs, were selected to construct a competing triple circRNA-miRNA-mRNA regulatory network. The network was visualized using Cytoscape (v3.10.1) to illustrate these regulatory interactions.

Furthermore, target genes within this regulatory network were subjected to functional annotation and signaling pathway analysis utilizing the Gene Ontology (GO) database (http://geneontology.org/ (accessed on 1 April 2021)) and the Kyoto Encyclopedia of Genes and Genomes (KEGG) database (http://www.genome.jp/kegg/ (accessed on 1 April 2021)). 

### 2.6. qRT-PCR Validation

To validate the accuracy of the RNA-seq results, four circRNAs and four miRNAs were randomly selected from each comparison group and subjected to qRT-PCR validation, with primer sequences detailed in Supplementary Table S1. β-actin and U6 were selected as reference genes, with their stability shown in Supplementary Figure S1. Relative quantification was performed using the 2^−ΔΔCt^ method, followed by one-way ANOVA and significance testing using SPSS 18.0.

To further confirm the circularity of the designed circRNA primers, PCR amplification was performed for all eight circRNAs using both cDNA and gDNA templates. The resulting products were analyzed by 1.5% agarose gel electrophoresis to validate their circular characteristics.

### 2.7. Binding Site Prediction

Prediction of binding sites was performed using the RNAhybrid software (v2.1.2) based on ncRNA sequence information, followed by dual-luciferase reporter assay for verification.

### 2.8. Recombinant Plasmids Preparation

Based on predicted interaction regions, mutant and wild-type sequences were designed and directionally cloned into the pCHECK2 plasmid backbone.

### 2.9. Cell Transfection

HEK293T cells were thawed and cultured in 25 cm^2^ flasks at 37 °C. Upon reaching 90% confluence, cells were trypsinized and seeded at equal density into 96-well plates. Triplicate biological replicates were prepared. Co-transfection was performed according to experimental groupings, with transfection completed the following day.

### 2.10. Reporter Signal Measurement

Plates were equilibrated at room temperature for 30 min. Luciferase Assay Reagent I (equal to culture medium volume) was added to each well, followed by gentle pipetting. After 10 min incubation, luminescence signals were measured using a microplate reader (BMG LABTECH, Ortenberg, Germany).

### 2.11. Normalization Control Measurement

An equal volume of Stop & Glo^®^ Reagent (Promega, Madison, WI, USA) was added to each well. Following gentle mixing and 10 min incubation at room temperature, renilla signals were recorded using the same instrument.

### 2.12. Data Normalization

The relative luminescence ratio was calculated by normalizing the reporter gene activity to the internal control activity for each individual sample.

## 3. Results

### 3.1. Sequencing Data Analysis

Ovarian tissues were collected from twelve samples: three one-month-old Small-tailed Han sheep (Som1, Som2, Som3), three six-month-old Small-tailed Han sheep (Stm1, Stm2, Stm3), three adult multiparous Small-tailed Han ewes (Sad1, Sad2, Sad3), and three adult multiparous Ujimqin sheep ewes (Ud1, Ud2, Ud3). Total RNA from all samples passed quality control, with both concentration and integrity meeting the requirements for sequencing, as detailed in Supplementary Table S2. Following sequencing, raw reads were filtered; the resulting data are presented in Table 1 and Table 2. For both mRNA and circRNA sequencing, over 98.5% of bases achieved a Q20 score and over 95% achieved a Q30 score in each sample. The GC content ranged from 45% to 50.5%. Similarly, for sRNA sequencing, Q20 and Q30 scores exceeded 95% in all samples, with GC content between 50.62% and 51.98%. These results demonstrate high-quality sequencing data with qualified quality control metrics, confirming their suitability for subsequent analyses.

### 3.2. Screening and Identification of circRNAs and miRNAs

**Figure 1:**
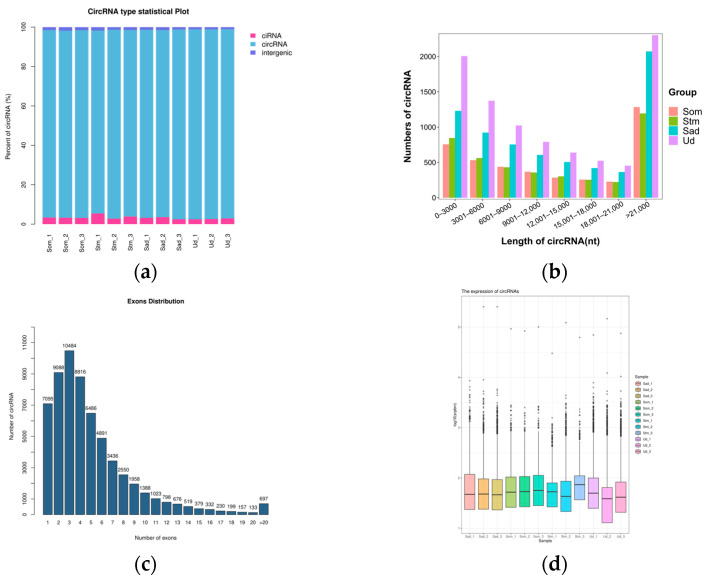
General Characteristics of circRNAs. (**a**): circRNA type statistics; (**b**): circRNA sequence length; (**c**): circRNA exon count distribution; (**d**): circRNA expression distribution map.

CircRNA identification results revealed that 4587, 3860, 4332, 3167, 6729, 2989, 7025, 6533, 7645, 8564, 11248, and 8435 circRNAs were detected in the 12 samples (Som1, Som2, Som3, Stm1, Stm2, Stm3, Sad1, Sad2, Sad3, Ud1, Ud2, Ud3), respectively. Further analysis of the genomic origins of the identified circRNAs demonstrated that exonic circRNAs constituted the predominant category, followed by intronic ciRNAs and intergeniccircRNAs (Figure 1a). The length of the identified circRNAs primarily ranged between 200 and 20,000 nucleotides (nt) (Figure 1b), and the majority were formed by the circularization of 2 to 4 exons (Figure 1c). Following normalization, the transcript expression levels of circRNAs were comparable across the different groups (Figure 1d).

**Figure 2:**
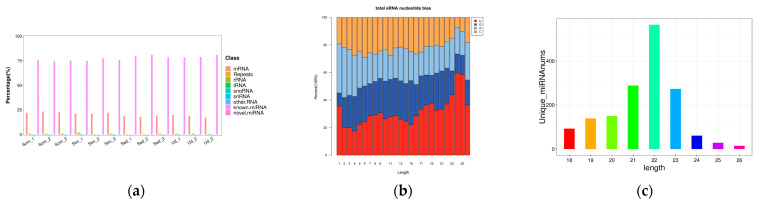
General characteristics of miRNA. (**a**): Categories of identified ncRNAs via sequencing in Som, Stm, Sad and Ud groups; (**b**): Bias distribution of the first base of miRNA sequence; (**c**): Length distribution of clean reads from identified miRNA fragments.

The miRNA annotation results revealed that novel miRNAs constituted the largest proportion, followed by known miRNAs and mRNAs. The proportions of all other types were nearly negligible (Figure 2a). The percentage of total rRNA serves as an indicator of sample quality. High-quality animal samples typically exhibit total rRNA content constituting less than 40% [18]. In this study, the total rRNA content in the different ovarian libraries was close to 0%, indicating exceptionally high sample quality. Furthermore, analysis of the first nucleotide preference for the identified miRNAs demonstrated that both known and predicted miRNAs exhibited a marked preference for uracil (U) at their 5′ termini (Figure 2b). The length distribution of miRNAs was highly consistent across all libraries, ranging predominantly from 20 to 24 nt. The most abundant miRNAs were 22 nt in length, consistent with typical miRNA sizes (Figure 2c).

### 3.3. Differential Expression Analysis

**Figure 3:**
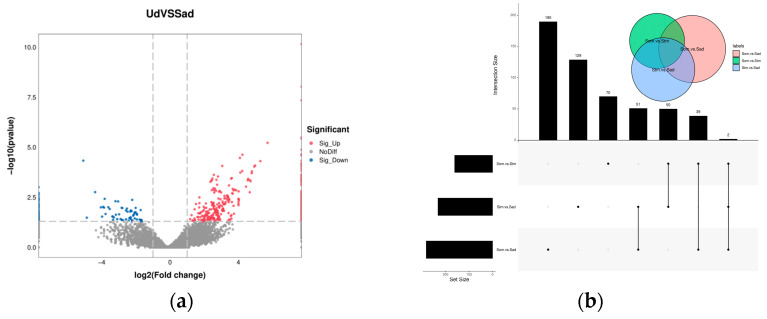
Volcano plot and Venn diagram of differentially expressed circRNAs. (**a**): Different fecundity groups; (**b**): different ovarian developmental stages.

In the differential expression analysis of circRNAs, we identified a total of 517 DECs between the different fertility groups (Sad vs. Ud), comprising 408 up-regulated and 109 down-regulated circRNAs (Figure 3a). For comparisons between different ovarian developmental stages: the Som vs. Stm comparison yielded 161 DECs (77 up-regulated, 84 down-regulated); Stm vs. Sad yielded 232 DECs (50 up-regulated, 182 down-regulated); and Som vs. Sad yielded 282 DECs (53 up-regulated, 229 down-regulated) (Figure 3b). The complete DECs were listed in Supplementary Tables S3–S6.

**Figure 4:**
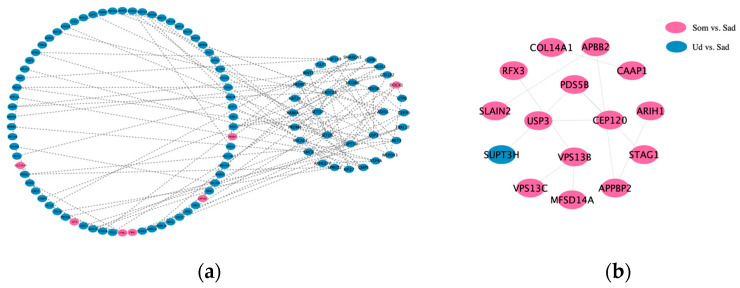
PPI analysis of DE circRNA host gene. (**a**): Up-regulated host gene; (**b**): down-regulated host gene.

To elucidate the relationships among DECs in different comparison groups, protein–protein interaction (PPI) networks were constructed for both up- and down-regulated host genes using interactions with a Confidence score ≥ 0.40. The results are presented in Figure 4. Analysis of the protein interactions within these networks revealed the following: In the up-regulated host gene network: The 102 up-regulated genes formed 113 interaction pairs. Hub genes identified within this network included *EXOC6B*, *SUPT3H*, *USP*3, *PDS5B*, and *NCOA*6. In the down-regulated host gene network: the 15 down-regulated genes formed 12 interaction pairs. Hub genes in this network were *VPS13B*, *PDS5B*, and *USP*3. Notably, *USP*3 emerged as a common hub gene shared between both networks. It exhibited significant interactions with several key nodes, including *PDS5B*, *CEP*120, *CEP*70, *KAT2B*, *FBXL*17, and *SUPT3H*. This suggests that *USP*3 may play a critical role and potentially influence female germ cell development.

**Figure 5:**
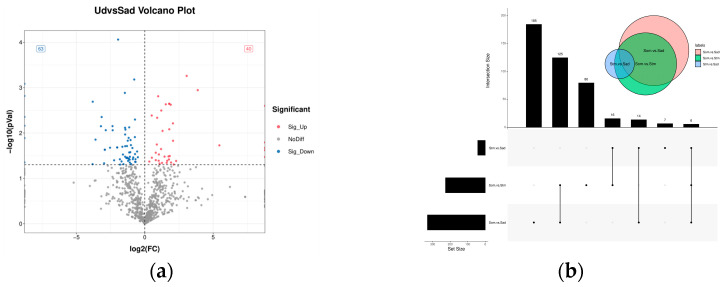
Volcano plot and Venn diagram of differentially expressed miRNAs. (**a**): Different fecundity groups; (**b**): different ovarian developmental stages.

In the screening analysis for DEMs, 103 DEMs were identified between the different fecundity comparison groups (Sad vs. Ud), comprising 40 up-regulated and 63 down-regulated miRNAs, as illustrated in Figure 5a. Comparative analysis of different ovarian developmental stages revealed 227 DEMs between the Som vs. Stm groups (170 up-regulated, 57 down-regulated), 43 DEMs between the Stm vs. Sad groups (22 up-regulated, 21 down-regulated), and 330 DEMs between the Som vs. Sad groups (279 up-regulated, 51 down-regulated), shown in Figure 5b. The complete DEMs were listed in Supplementary Tables S7–S10.

**Figure 6:**
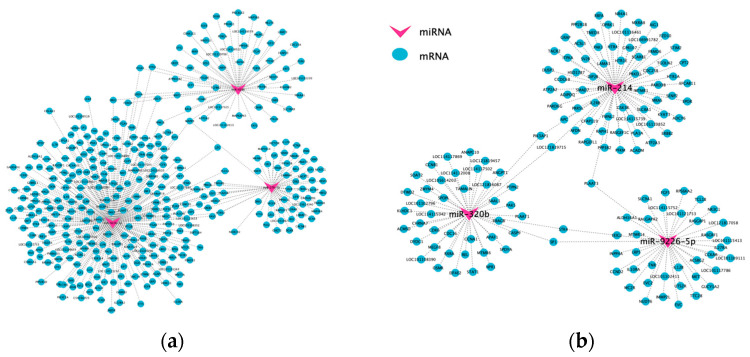
Overview of miRNA and target genes networks. (**a**): The DEM-DEG interaction network in the Ud vs. Sad group; (**b**): the DEM-DEG interaction network in the Som vs. Stm vs. Sad group.

To elucidate the regulatory role of miRNAs in female sheep reproduction, we removed novel genes and screened for reproduction-associated DEMs and DEGs from each comparative group. Subsequently, miRNA-mRNA regulatory networks were constructed to explore interactions between candidate DEMs and their target DEGs in ovarian function. Following comparisons between fertility groups, we identified and constructed a focused DEM-DEG regulatory network comprising 3 known DEMs (miR-214, miR-320b, miR-9226-5p) and 143 DEGs (Figure 6a). Similarly, analysis of ovarian developmental stages revealed a compact ceRNA network involving 3 known miRNAs (miR-9226-5p, miR-27b-3p, miR-4286) and 344 DEGs (Figure 6b).

### 3.4. ceRNA Network Analysis

**Figure 7:**
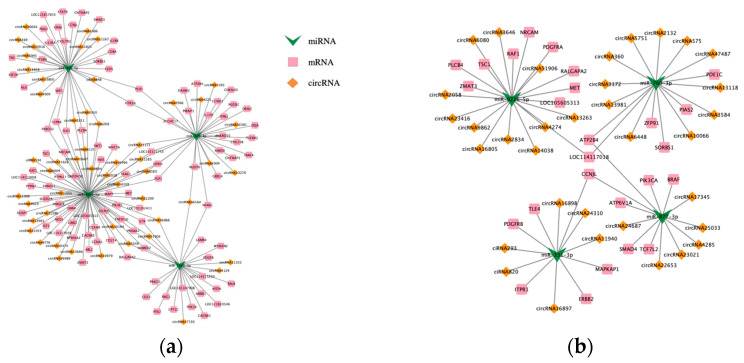
ceRNA regulatory network. (**a**): Different fecundity groups; (**b**): different ovarian developmental stages.

Focusing on DEMs, we integrated DECs acting as miRNA sponges and DEGs targeted by DEMs. DEMs were identified from the intersection of two comparative groups, and a regulatory network was constructed. Due to the excessively large network size, core genes were selected as the intersection of the top 10 miRNAs with the highest interaction probability across relationship pairs. This approach yielded two concise ceRNA networks relevant to our study, as illustrated in Figure 7. The ceRNA network derived from the comparison of different fecundity groups (Ud vs. Sad) comprised 116 DECs, 46 DEMs, and 82 DEGs (Figure 7a). The network obtained from comparing different ovarian developmental stages (Som vs. Stm vs. Sad) consisted of 186 DECs, 143 DEMs, and 338 DEGs, forming a circRNA-miRNA-mRNA regulatory network (Figure 7b).

**Figure 8:**
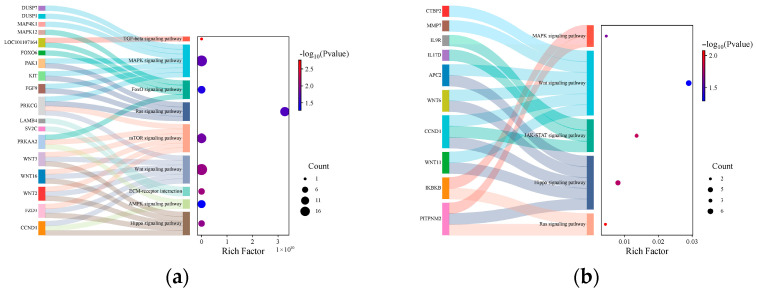
Bubble map of target gene KEGG-enriched Sankey. (**a**): Different fecundity groups; (**b**): different ovarian developmental stages.

Further GO and KEGG enrichment analyses were performed on the target genes within the ceRNA regulatory network. In the comparison groups of sheep with differing fecundity, several biological processes and pathways related to reproduction were significantly enriched (Figure 8a), including the MAPK signaling pathway, JAK-STAT signaling pathway, and WNT signaling pathway. In the comparison groups of different developmental stages, genes such as *PAK*1, *KIT*, *FGF*9, and *PRKCG* were found to be concurrently involved in both the MAPK signaling pathway and the Ras signaling pathway. Additionally, genes including *WNT*3, *WNT*16, *WNT*2, and *FZD*3 participated simultaneously in both the WNT signaling pathway and the Hippo signaling pathway (Figure 8b). These genes represent potential candidate genes implicated in reproductive processes.

**Figure 9:**
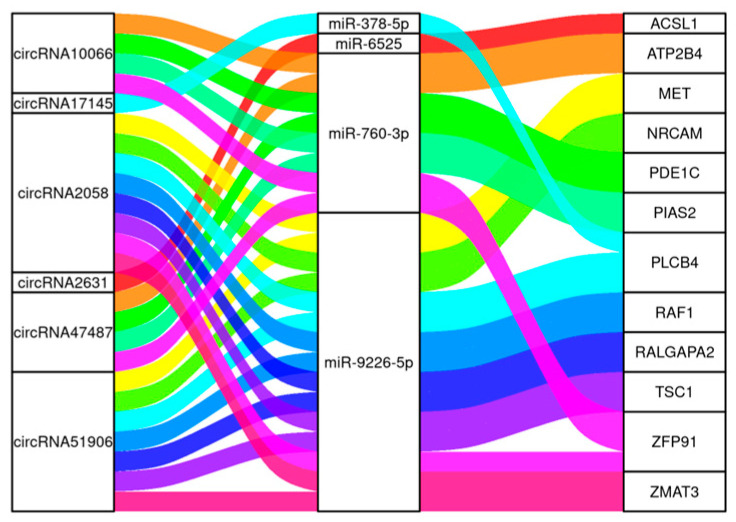
ceRNA network Sankey diagram.

Taken together, we constructed a circRNA-miRNA-mRNA regulatory network (Figure 9) by selecting genes functionally associated with reproduction that were co-regulated in the ceRNA networks of both comparison groups. This network comprised 4 miRNAs, 6 circRNAs, and 12 mRNAs, forming 22 interaction pairs. Functional analysis implicated the *MET* gene in ovarian development and follicular dominance. Consequently, the circRNA2058-miR-9226-5p-*MET* axis was selected for validation using a dual-luciferase reporter assay to confirm the predicted targeting relationships.

### 3.5. qRT-PCR and Sanger Verification

**Figure 10:**
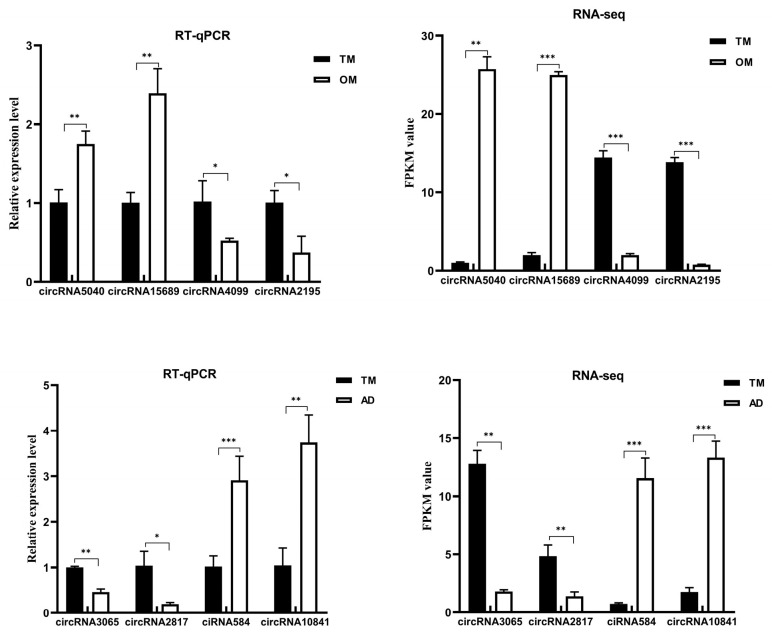
qRT-PCR validation of the RNA-Seq data for circRNAs. (* *p* < 0.05, ** *p* < 0.01, *** *p* < 0.001).

Validation of eight selected circRNAs by qRT-PCR is presented in Figure 10. The expression trends observed in both RNA-seq and qRT-PCR analyses were consistent, indicating the reliability of the RNA-seq results. Validation of the designed circRNA primers demonstrated their specificity: amplification using cDNA templates yielded bands of the expected size, while no bands were produced with genomic DNA (gDNA) templates (Supplementary Figure S2). Sanger sequencing of the cDNA-derived amplicons confirmed that the sequences spanning the back-splice junctions matched the circRNA sequencing data, verifying the circular nature of these transcripts (Supplementary Figure S2). In this study, we identified circRNAs based on their back-splicing junctions using divergent primers and Sanger sequencing. While this is a widely accepted computational and initial experimental approach, it is important to note that more rigorous biochemical assays, such as resistance to RNase R digestion or subcellular fractionation, would further solidify the circular conformation of these RNAs. The inability to include such assays herein represents a limitation of our current validation, and confirming the circular nature with these methods will be a priority in our future functional investigations.

**Figure 11:**
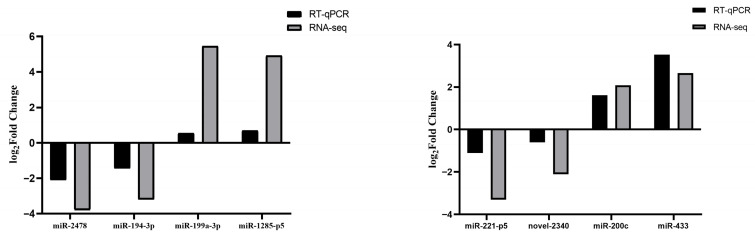
qRT-PCR validation of the RNA-Seq data for miRNAs.

The relative quantification results of eight randomly selected miRNAs from the two comparison groups are shown in Figure 11. Their expression levels agreed with the RNA-seq data, further demonstrating the accuracy and reproducibility of the RNA-seq results.

### 3.6. Dual-Luciferase Reporter Assay

**Figure 12:**
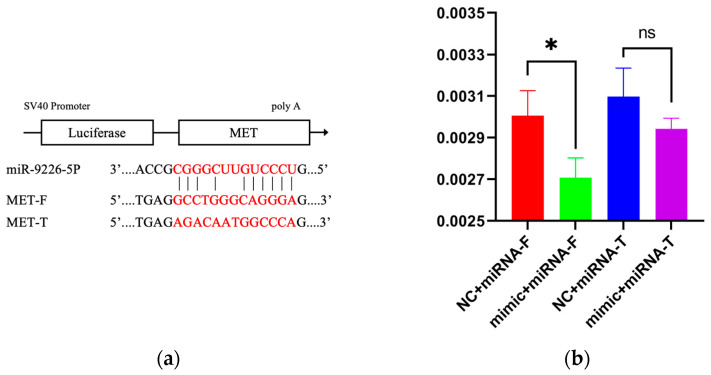

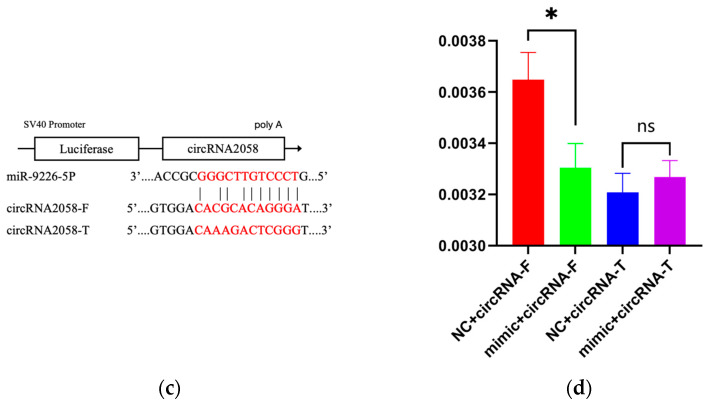
Targeting relationship validation. (**a**): Complementary nucleotide sequence of *MET*-3′UTR and miR-9226-5p; (**b**): *MET* and miR-9226-5p double luciferase report results; (**c**): complementary nucleotide sequence of circRNA2058-3′UTR and miR-9226-5p; (**d**): circRNA2058 and miR-9226-5p double luciferase report results. (* *p* < 0.05, ns—not significant).

As depicted in Figure 12a, the base pairing occurs between the 3′UTR region of the *MET* gene and miR-9226-5p. Luciferase reporter assays (Figure 12b) demonstrated that miR-9226-5p significantly suppressed the luciferase activity of *MET*-F compared to the NC control group (*p* < 0.05), indicating specific binding between them. Following site-directed mutagenesis, this regulatory effect was abolished, as evidenced by the lack of a statistically significant difference (*p* > 0.05) in luciferase activity for the mutant construct *MET*-T, confirming successful mutant construction. The binding site for miR-9226-5p within the sequence of circRNA2058 (Figure 12c) was validated by luciferase assays using a reporter construct containing the BSJ-spanning fragment (Figure 12d). Transfection with miR-9226-5p significantly reduced the reporter activity of circRNA2058-F (*p* < 0.05), whereas the luciferase intensity of the mutant circRNA2058-T remained stable (*p* > 0.05). These results successfully confirm the interaction between this circular RNA and the miRNA.

## 4. Discussion

Elucidating the genetic basis of prolificacy traits is critical for molecular breeding in sheep. The ovarian microenvironment, a central regulatory hub within the female reproductive axis, critically governs follicular development dynamics and ovulation rates in mammals, thereby profoundly influencing reproductive success. Consequently, ovarian tissue serves as a primary sequencing target for identifying key functional genes associated with multiple lambing and high fecundity. Emerging research highlights ncRNAs as pivotal regulators within ovarian gene networks, offering significant theoretical insights into ovine reproductive mechanisms. Among these, circRNAs exhibit exceptional intracellular stability and resistance to degradation due to their closed-loop structure. Functioning as efficient molecular sponges, circRNAs sequester miRNAs to derepress their target mRNAs, establishing ceRNA networks. Existing studies confirm that ceRNA networks play crucial roles in mammalian reproductive hormone synthesis, follicular maturation/atresia, and early embryonic development regulation [19,20,21]. Therefore, elucidating the expression dynamics and functional roles of ceRNA networks in sheep ovaries—across varying fertility phenotypes and developmental stages—holds substantial biological significance for deciphering the regulatory mechanisms underlying ovine reproductive performance.

This study employed RNA-Seq technology coupled with differential expression analysis to profile mRNA and ncRNA expression in groups differing in fecundity and at various ovarian developmental stages. Subsequent construction of a ceRNA network and bioinformatic analyses identified several reproduction-associated signaling pathways. These include the MAPK, JAK-STAT, and WNT signaling pathways (in the fecundity comparison groups), and the MAPK, Ras, WNT, and Hippo signaling pathways (in the ovarian developmental stage groups). The Hippo signaling pathway primarily regulates organ size and contact inhibition [22,23]. Within the ovary, its inhibited state may indirectly enhance ovulation rates by promoting primordial follicle activation [24] and granulosa cell proliferation [25]. This mechanism potentially contributes to variations in sheep fecundity. Studies indicate that all components of the Hippo pathway are expressed in the ovary, where it plays a critical role in regulating mammalian germ cell proliferation, follicular development, and corpus luteum formation [26]. The JAK-STAT signaling pathway is highly activated in dominant follicles, regulating granulosa cell proliferation and differentiation. JAK3 was found to interact with proteins such as LEPROTL1, INHBA, and CDKN1B, influencing granulosa cell viability by phosphorylating STAT family members, thereby determining follicular maturation or atresia [27]. The MAPK signaling pathway serves as a key messenger transmitting extracellular signals from the cell membrane to the nucleus [28]. It acts as a central molecular hub in regulating ovarian function, significantly influencing reproductive efficiency by coordinating follicular development, hormone synthesis, and cell fate determination [29,30]. The Ras signaling pathway is a key regulator of cell proliferation, differentiation, and apoptosis, and plays a critical role in the mammalian reproductive system. Its functions include regulating follicular development, oocyte maturation, steroid hormone synthesis, and ovulation [31,32]. The Wnt signaling pathway, a crucial pathway in reproduction, has been extensively investigated. Studies have demonstrated that miR-458b-5p regulates follicular development in the chicken ovary by targeting *CTNNB*1 (β-catenin), a key gene within the Wnt signaling pathway [33]. Furthermore, microRNA-3061 was shown to induce premature ovarian insufficiency in mice by downregulating the expression of Wnt signaling pathway genes [34]. Additionally, research revealed that miR-29c targets *FZD*4, a Wnt pathway receptor, thereby inhibiting both Wnt signaling activation and granulosa cell (GC) apoptosis mediated by *SMAD*4-induced *FZD*4 [35].

Comprehensive analysis of genes enriched in reproduction-related pathways identified *MET* as a common hub gene within the reproductive networks of both comparison groups, suggesting its critical role in reproductive regulation. The *MET* gene encodes the sole receptor for hepatocyte growth factor (HGF). Their specific binding interaction regulates gonadal development and germ cell proliferation [36]. Previous research has proposed *MET* as a key candidate gene influencing reproductive traits in Chinese Holstein cattle [37]. Furthermore, *MET* directly acts on vascular endothelial cells to modulate angiogenesis, enhance capillary density, and increase blood flow, thereby influencing injury repair and embryonic development [38,39]. During bovine follicular formation, Parrott’s team demonstrated that *MET* in both theca and granulosa cells, in concert with estrogen and luteinizing hormone (LH), mediates granulosa cell proliferation by modulating the localized expression of HGF, thereby driving follicular maturation [40]. Collectively, these findings underscore the significant role of the *MET* gene in the genetic architecture of reproductive traits.

Comprehensive analysis of the constructed ceRNA network, validated using the dual-luciferase reporter assay system, ultimately confirmed that miR-9226-5p directly targets and regulates *MET* expression. Furthermore, circRNA2058 was demonstrated to function as a molecular sponge for miR-9226-5p. These findings collectively indicate that the circRNA2058-miR-9226-5p-*MET* ceRNA axis plays a potential regulatory role in the high fecundity trait of sheep. It should be noted that the dual-luciferase validation experiments in this study were performed in HEK293T cells. While this is a reliable method for demonstrating direct molecular interactions, it lacks the ovarian-specific cellular context. In future studies, we will ultimately confirm the functionality of this regulatory pathway in a physiologically relevant setting by overexpressing/knocking down circRNA2058 in primary sheep granulosa cells or ovarian tissues and directly examining subsequent changes in *MET* mRNA and protein levels.

## 5. Conclusions

This study employed transcriptome-wide co-analysis to construct a multi-dimensional ceRNA network implicated in the regulation of ovine ovarian development. From this network, we identified key candidate genes capable of concurrently modulating reproductive capacity across different sheep breeds and developmental stages. Particular focus was placed on the core regulatory axis comprising circRNA2058-miR-9226-5p-*MET*. The targeting relationships within this axis were validated using the dual-luciferase reporter assay system. These findings provide novel targets and a theoretical foundation for elucidating the genetic mechanisms underlying high fecundity in sheep, developing molecular breeding markers, and preventing reproductive disorders. Consequently, this work holds significant scientific and practical implications for enhancing ovine reproductive efficiency and advancing efficient breeding practices.

## Figures and Tables

**Table 1:** mRNA and circRNA sequencing data production quality.

Sample	Raw Data	Valid Data	Mapped Reads/%	Q20%	Q30%	GC Content/%
Som_1	95,584,162	92,544,232	87,671,478 (94.73%)	99.97	97.81	45.00
Som_2	93,194,002	90,150,580	85,397,972 (94.73%)	99.97	97.84	44.00
Som_3	93,566,726	90,707,218	84,656,216 (93.33%)	99.96	97.58	45.00
Stm_1	93,780,650	90,919,404	86,062,585 (94.66%)	99.97	97.78	45.00
Stm_2	91,917,068	88,670,432	82,933,703 (93.53%)	99.98	98.30	49.00
Stm_3	71,302,642	67,362,198	60,153,911 (89.30%)	98.50	94.59	45.00
Sad_1	94,092,386	91,107,390	86,158,306 (94.57%)	99.96	97.78	45.00
Sad_2	97,790,686	94,803,474	89,533,252 (94.44%)	99.96	97.71	45.00
Sad_3	89,825,602	86,701,034	80,875,819 (93.28%)	99.97	98.16	48.50
Ud_1	73,320,580	70,898,902	65,359,324 (92.19%)	99.85	97.83	47.00
Ud_2	92,249,184	87,955,300	82,065,791 (93.30%)	99.77	97.86	50.50
Ud_3	71,516,612	68,959,698	63,802,517 (92.52%)	99.81	97.60	48.00

**Table 2:** sRNA sequencing data production quality.

Sample	Raw Data	Valid Data	Mapped Reads/%	Q20%	Q30%	GC Content/%
Som_1	13,916,096	9,366,661	9,257,071 (98.83%)	97.91	96.89	51.78
Som_2	12,153,463	7,973,465	7,861,836 (98.60%)	97.67	96.68	51.60
Som_3	15,083,990	9,398,231	9,259,137 (98.52%)	97.02	95.67	51.78
Stm_1	9,076,908	5,229,613	5,161,628 (98.70%)	97.74	96.52	51.98
Stm_2	8,729,542	7,235,145	7,180,158 (99.24%)	97.63	96.44	51.03
Stm_3	10,569,235	5,463,213	5,393,284 (98.72%)	97.35	96.04	50.62
Sad_1	10,567,624	6,971,109	6,898,609 (98.96%)	97.92	96.91	51.40
Sad_2	9,796,916	7,951,578	7,863,315 (98.89%)	97.92	96.96	51.22
Sad_3	10,036,758	6,868,427	6,794,248 (98.92%)	97.29	96.07	51.11
Ud_1	11,079,361	6,789,313	6,743,146 (99.32%)	99.24	97.13	51.14
Ud_2	11,405,958	7,210,142	7,160,392 (99.31%)	99.26	97.10	50.87
Ud_3	11,156,801	7,387,622	7,321,133 (99.10%)	99.28	97.18	50.94

## Data Availability

The raw RNA-seq data generated in this study have been deposited in the NCBI Sequence Read Archive (SRA) under the BioProject accession number PRJNA1289050 and PRJNA1253307.

## Supplementary Materials

The following supporting information can be downloaded at https://www.mdpi.com/article/10.3390/ani15213077/s1, Figure S1: the stability of reference genes; Figure S2: verification of DECs; Table S1: qRT-PCR primers sequence information; Table S2: Detection results of total RNA quality; Table S3: complete DECs lists (Ud vs Sad); Table S4: complete DECs lists (Som vs. Stm); Table S5: complete DECs lists (Stm vs. Sad); Table S6: complete DECs lists (Som vs. Sad); Table S7: complete DEMs lists (Ud vs. Sad); Table S8: complete DEMs lists (Som vs. Stm); Table S9: complete DEMs lists (Stm vs. Sad); Table S10: complete DEMs lists (Som vs. Sad).

## Abbreviations

The following abbreviations are used in this manuscript:

DECs	differentially expressed circRNAs
DEMs	differentially expressed miRNAs
DEGs	differentially expressed mRNAs
ncRNA	non-coding RNA
ceRNA	competing endogenous RNA
GO	Gene Ontology
KEGG	Kyoto Encyclopedia of Genes and Genomes
NGS	Next-generation sequencing
