# Supplementary material for: Integrated Whole-Transcriptome Analysis to Elucidate the Core Regulatory Network of circRNA Involved in Ovarian Development and Reproductive Capacity Differences in Sheep: circRNA2058-miR-9226-5p-MET Axis
Source: Animals (Basel). 2025 Oct 23;15(21):3077. doi: 10.3390/ani15213077 (PMC12607438; doi:10.3390/ani15213077)
Supplement: Supplementary file 1 [file animals-15-03077-s001.zip › animals-3877789-supplementary.pdf]

Table S1 qRT-PCR prsimers sequence information

Gene name	Sequence information	Length/bp
β-actin	F: GATCTGGCACCACACCTTCTA	115
	R: GATCTGGGTCATCTTCTCACG	
circRNA4099	F:GGAGAAATCCTACCCCCTCTACAC	196
	R: CATCTCAATTGATTCTGTGGATTTTC	
circRNA2195	F:GCTTTGAATGGTGTGGAGGATG	208
	R:CGCAGAGGGTCTGGGAGAAG	
circRNA15689	F:CACAGAGTCAAGAGACTAACGGGC	179
	R:CTCTCCAACTTCCACAATTTGCTC	
circRNA5040	F:CAAAACGTGAAGAAATAATGGAGAG	206
	R:CACATTTGGATCCAACAACTGATG	
circRNA10841	F:ACTCTGTGCTTTATGGCAAGGTAG	206
	R:GTTGGTTAAGGGTATTTGTGCATC	
circRNA584	F: CAGCTGGCCATAGGCAACG	180
	R:TCTCGATCAGCAGAGAGATTCAATG	
circRNA2817	F: TTGATCCAGAATTCTCCTGAGTGC	190
	R:GTTGACTATATAATTGTTGAGAAATAACTG	
circRNA1367	F:GGAAGACACTCTGTGCTTTATGGC	213
	R: GTTGGTTAAGGGTATTTGTGCATC	
U6	F:TGAAGCGTGCTCGCTTCGGC	/
miR-199a	F:CAGCCTTAACAGTAGTCTGCACATT	/
miR-194	F:TATAGTCCAGTGGGGCTGCTGT	/
miR-2478	F:CGCGTCGTATCCCACTTCTG	/
miR-1285-p5	F:CGGATCAGTAGTGGGATCGC	/
miR-433-3p	F:AACGGCATCATGATGGGCTCC	/
miR-200c	F:AAGCGCCTTAATACTGCCGGG	/
novel-2340	F:ACGAGATCCTCCTGACTCCAGG	/
miR-221-p5	F:CCTGCTGGACCTGGCATACAAT	/

Table S2 Detection results of total RNA quality

Groups	Samples	concentration (μg/μL)	RIN	Amount (μg)
Som	Som-1	0.26	9.30	11.02
	Som-2	0.31	9.10	13.10
	Som-3	0.28	8.90	12.06
Stm	Stm-1	0.24	8.80	10.26
	Stm-2	0.83	7.30	34.71
	Stm-3	0.35	8.70	13.78
Sad	Sad-1	0.38	8.50	15.75
	Sad-2	0.31	8.60	12.95
	Sad-3	0.24	7.20	9.96
Ud	Ud-1	0.49	8.20	22.06
	Ud-2	0.05	7.50	2.26
	Ud-3	0.07	10.00	3.23

Table S3　complete DECs lists (Ud vs Sad)

Accession	Ud1_srpbm	Ud2_srpbm	Ud3_srpbm	Sad2_srpbm	Sad1_srpbm	Sad3_srpbm	log_2FC	regulation	pvalue	qvalue
circRNA5040	1124.61	774.57	935.07	0	0	0	inf	up	0.00	0.00
circRNA15689	724.75	464.74	519.48	0	0	0	inf	up	0.00	0.00
circRNA4085	549.81	361.47	519.48	0	0	0	inf	up	0.00	0.00
circRNA13134	374.87	258.19	228.57	0	0	0	inf	up	0.00	0.01
circRNA13969	324.89	206.55	311.69	0	0	0	inf	up	0.00	0.01
circRNA11940	574.80	180.73	498.70	23.97	0	0	5.71	up	0.00	0.01
circRNA16753	374.87	193.64	187.01	0	0	0	inf	up	0.00	0.02
circRNA13959	249.91	154.91	270.13	0	0	0	inf	up	0.00	0.02
circRNA2058	599.79	245.28	477.92	0	23.62	46.44	4.24	up	0.00	0.02
circRNA16777	324.89	219.46	145.45	0	0	0	inf	up	0.00	0.02
circRNA7338	274.91	348.56	124.68	0	0	0	inf	up	0.00	0.02
circRNA5925	2624.10	2749.73	2971.43	0	165.31	325.06	4.09	up	0.00	0.03
circRNA4099	0	25.82	0	263.70	354.24	255.40	-5.08	down	0.00	0.03
circRNA404	274.91	116.19	249.35	0	0	0	inf	up	0.00	0.03
circRNA16520	1274.56	374.38	166.23	0	0	46.44	5.29	up	0.00	0.03
circRNA13981	199.93	116.19	249.35	0	0	0	inf	up	0.00	0.04
circRNA1094	174.94	154.91	187.01	0	0	0	inf	up	0.00	0.04
circRNA17163	199.93	167.82	145.45	0	0	0	inf	up	0.00	0.04
circRNA13572	249.91	103.28	249.35	0	0	0	inf	up	0.00	0.04
circRNA1756	349.88	167.82	228.57	0	23.62	0	4.98	up	0.00	0.04
circRNA16897	1499.49	0	1246.75	0	0	0	inf	up	0.00	0.04
circRNA1204	1824.38	1342.59	1142.86	191.78	47.23	278.62	3.06	up	0.00	0.04
circRNA5035	199.93	258.19	249.35	0	0	23.22	4.93	up	0.00	0.04
circRNA17182	174.94	129.10	207.79	0	0	0	inf	up	0.00	0.04
ciRNA305	249.91	219.46	83.12	0	0	0	inf	up	0.00	0.05
circRNA14209	149.95	180.73	145.45	0	0	0	inf	up	0.00	0.06
circRNA8541	449.85	219.46	228.57	47.95	0	0	4.23	up	0.00	0.06
circRNA14316	249.91	167.82	228.57	0	0	23.22	4.80	up	0.00	0.06
circRNA16804	249.91	284.01	270.13	0	0	46.44	4.11	up	0.00	0.06
circRNA7589	149.95	258.19	249.35	23.97	0	0	4.78	up	0.00	0.07
circRNA9845	149.95	116.19	207.79	0	0	0	inf	up	0.00	0.07
circRNA2707	599.79	735.84	893.51	95.89	141.70	139.31	2.56	up	0.00	0.07
circRNA17420	74.97	245.28	187.01	0	0	0	inf	up	0.00	0.07
circRNA4236	174.94	180.73	103.90	0	0	0	inf	up	0.00	0.08
circRNA1279	674.77	568.02	415.58	119.86	70.85	46.44	2.81	up	0.00	0.08
circRNA23181	224.92	77.46	187.01	0	0	0	inf	up	0.00	0.09
circRNA17211	149.95	167.82	124.68	0	0	0	inf	up	0.00	0.09
circRNA6443	399.86	400.19	374.03	95.89	0	0	3.61	up	0.00	0.09
circRNA13780	124.96	167.82	145.45	0	0	0	inf	up	0.00	0.09

circRNA3455	1349.54	890.76	1038.96	95.89	188.93	301.84	2.48	up	0.00	0.09
circRNA6959	224.92	374.38	249.35	47.95	0	23.22	3.58	up	0.00	0.09
circRNA4243	99.97	232.37	124.68	0	0	0	inf	up	0.00	0.09
circRNA7253	224.92	219.46	124.68	23.97	0	0	4.57	up	0.00	0.09
circRNA16799	124.96	180.73	124.68	0	0	0	inf	up	0.00	0.09
circRNA13691	424.85	477.65	498.70	95.89	70.85	46.44	2.72	up	0.00	0.09
circRNA14398	0	413.10	602.60	0	0	0	inf	up	0.00	0.09
circRNA12591	299.90	142.00	145.45	0	0	23.22	4.66	up	0.00	0.09
circRNA6680	249.91	167.82	124.68	0	0	23.22	4.55	up	0.00	0.09
circRNA3279	774.73	735.84	519.48	119.86	165.31	92.87	2.42	up	0.00	0.09
circRNA4206	699.76	839.12	1018.18	95.89	212.54	162.53	2.44	up	0.00	0.09
circRNA5751	99.97	167.82	166.23	0	0	0	inf	up	0.00	0.09
circRNA2638	324.89	193.64	103.90	0	23.62	0	4.72	up	0.00	0.10
circRNA23285	149.95	90.37	187.01	0	0	0	inf	up	0.00	0.10
circRNA17293	124.96	193.64	103.90	0	0	0	inf	up	0.00	0.10
circRNA6178	724.75	1213.49	851.95	143.84	212.54	185.75	2.36	up	0.00	0.10
circRNA8052	249.91	51.64	187.01	0	0	0	inf	up	0.00	0.11
circRNA4445	324.89	400.19	394.81	71.92	47.23	46.44	2.76	up	0.00	0.13
circRNA5837	174.94	51.64	249.35	0	0	0	inf	up	0.00	0.13
circRNA9808	199.93	90.37	124.68	0	0	0	inf	up	0.00	0.13
circRNA3074	449.85	271.10	311.69	0	94.46	0	3.45	up	0.00	0.13
circRNA11352	149.95	103.28	270.13	23.97	0	0	4.45	up	0.00	0.15
circRNA3696	0	0	0	47.95	354.24	139.31	-inf	down	0.00	0.16
circRNA7153	399.86	374.38	457.14	47.95	70.85	92.87	2.54	up	0.00	0.18
circRNA2778	1499.49	1523.32	332.47	143.84	188.93	208.97	2.63	up	0.00	0.18
circRNA2407	199.93	193.64	207.79	0	47.23	0	3.67	up	0.00	0.19
circRNA3172	124.96	296.92	228.57	0	47.23	0	3.78	up	0.00	0.20
circRNA10916	474.84	451.83	477.92	47.95	70.85	139.31	2.44	up	0.00	0.20
circRNA3646	674.77	258.19	477.92	95.89	47.23	92.87	2.58	up	0.00	0.20
circRNA14774	374.87	180.73	124.68	0	0	46.44	3.87	up	0.00	0.20
circRNA8476	149.95	232.37	228.57	0	0	46.44	3.72	up	0.00	0.20
circRNA1745	2199.25	2491.54	2119.48	71.92	236.16	742.99	2.70	up	0.00	0.21
circRNA13950	149.95	77.46	145.45	0	0	0	inf	up	0.00	0.21
circRNA16045	274.91	245.28	187.01	23.97	23.62	46.44	2.91	up	0.00	0.21
circRNA13412	74.97	438.92	249.35	0	47.23	0	4.01	up	0.00	0.22
circRNA5150	274.91	206.55	207.79	47.95	23.62	23.22	2.86	up	0.00	0.22
circRNA27816	99.97	103.28	145.45	0	0	0	inf	up	0.00	0.22
circRNA2631	0	25.82	0	143.84	94.46	301.84	-4.39	down	0.00	0.22
circRNA8274	99.97	142.00	103.90	0	0	0	inf	up	0.00	0.22
circRNA1793	399.86	64.55	124.68	0	0	23.22	4.67	up	0.00	0.22
circRNA3440	1949.33	1536.23	1350.65	0	236.16	371.50	2.99	up	0.00	0.23

circRNA5058	349.88	245.28	540.26	47.95	70.85	69.66	2.59	up	0.00	0.23
circRNA15659	324.89	0	374.03	0	0	0	inf	up	0.00	0.23
circRNA4118	0	0	0	47.95	47.23	510.81	-inf	down	0.00	0.23
circRNA17138	174.94	142.00	270.13	0	47.23	0	3.64	up	0.00	0.24
circRNA13799	299.90	167.82	124.68	0	0	46.44	3.67	up	0.00	0.24
circRNA6770	0	335.65	228.57	0	0	0	inf	up	0.00	0.24
circRNA23607	149.95	77.46	124.68	0	0	0	inf	up	0.00	0.24
circRNA11053	0	0	0	143.84	70.85	185.75	-inf	down	0.00	0.25
circRNA2195	0	0	0	143.84	94.46	139.31	-inf	down	0.00	0.25
circRNA4095	99.97	129.10	103.90	0	0	0	inf	up	0.00	0.25
circRNA16805	174.94	64.55	124.68	0	0	0	inf	up	0.00	0.27
circRNA17534	524.82	271.10	166.23	95.89	0	0	3.33	up	0.00	0.28
circRNA2138	99.97	206.55	187.01	23.97	23.62	0	3.37	up	0.00	0.28
circRNA10962	374.87	219.46	249.35	0	0	92.87	3.18	up	0.00	0.29
circRNA17193	199.93	64.55	103.90	0	0	0	inf	up	0.00	0.29
circRNA3753	0	0	0	143.84	165.31	69.66	-inf	down	0.00	0.29
circRNA4852	124.96	90.37	103.90	0	0	0	inf	up	0.00	0.30
circRNA27903	124.96	90.37	103.90	0	0	0	inf	up	0.00	0.30
circRNA575	99.97	232.37	166.23	23.97	23.62	0	3.39	up	0.00	0.30
circRNA13745	149.95	167.82	187.01	47.95	0	0	3.40	up	0.00	0.31
circRNA1177	2299.21	1252.22	1870.13	407.54	637.63	603.68	1.72	up	0.00	0.32
circRNA3278	99.97	77.46	145.45	0	0	0	inf	up	0.00	0.33
circRNA9595	224.92	38.73	124.68	0	0	0	inf	up	0.00	0.33
circRNA12356	499.83	271.10	145.45	95.89	0	0	3.26	up	0.00	0.33
circRNA9254	0	0	0	167.81	0	417.93	-inf	down	0.00	0.33
circRNA2991	274.91	180.73	187.01	0	70.85	0	3.18	up	0.00	0.33
circRNA24724	149.95	116.19	62.34	0	0	0	inf	up	0.00	0.33
ciRNA69	0	0	0	95.89	165.31	92.87	-inf	down	0.00	0.33
circRNA4961	324.89	206.55	311.69	71.92	0	46.44	2.83	up	0.00	0.33
circRNA4069	0	0	0	47.95	283.39	92.87	-inf	down	0.00	0.33
circRNA15421	124.96	154.91	103.90	23.97	0	0	4.00	up	0.00	0.33
ciRNA81	0	0	41.56	143.84	330.62	116.09	-3.83	down	0.00	0.33
circRNA7725	124.96	103.28	249.35	0	23.62	23.22	3.35	up	0.00	0.33
circRNA16617	99.97	232.37	187.01	0	47.23	0	3.46	up	0.00	0.33
circRNA1615	199.93	154.91	187.01	0	23.62	46.44	2.95	up	0.00	0.34
circRNA10066	0	0	0	167.81	70.85	116.09	-inf	down	0.00	0.35
circRNA4715	99.97	154.91	124.68	0	0	23.22	4.03	up	0.00	0.35
circRNA8705	124.96	116.19	145.45	0	23.62	0	4.03	up	0.00	0.35
ciRNA650	74.97	90.37	145.45	0	0	0	inf	up	0.00	0.35
circRNA239	324.89	361.47	124.68	1678.09	1180.79	905.52	-2.21	down	0.00	0.35
circRNA6448	124.96	103.28	83.12	0	0	0	inf	up	0.00	0.35

circRNA2100	0	0	0	431.51	141.70	0	-inf	down	0.00	0.35
circRNA13338	99.97	167.82	249.35	0	0	46.44	3.48	up	0.00	0.35
circRNA3060	124.96	77.46	103.90	0	0	0	inf	up	0.00	0.36
circRNA17153	1149.61	697.11	561.04	0	0	232.18	3.37	up	0.00	0.36
circRNA13821	0	0	0	95.89	118.08	116.09	-inf	down	0.00	0.36
circRNA8414	99.97	90.37	103.90	0	0	0	inf	up	0.00	0.36
circRNA16722	124.96	167.82	41.56	0	0	0	inf	up	0.00	0.36
circRNA11062	74.97	142.00	83.12	0	0	0	inf	up	0.00	0.37
circRNA13360	149.95	77.46	83.12	0	0	0	inf	up	0.00	0.37
circRNA3091	74.97	0	0	287.67	259.77	139.31	-3.20	down	0.00	0.38
circRNA5387	174.94	129.10	83.12	23.97	0	0	4.01	up	0.01	0.38
circRNA17145	0	38.73	41.56	215.75	212.54	232.18	-3.04	down	0.01	0.38
circRNA8299	149.95	206.55	249.35	71.92	0	0	3.07	up	0.01	0.38
circRNA5962	49.98	129.10	124.68	0	0	0	inf	up	0.01	0.38
circRNA3179	74.97	116.19	103.90	0	0	0	inf	up	0.01	0.38
circRNA2979	124.96	116.19	62.34	0	0	0	inf	up	0.01	0.38
circRNA13524	99.97	180.73	41.56	0	0	0	inf	up	0.01	0.38
circRNA22974	174.94	129.10	145.45	0	47.23	0	3.25	up	0.01	0.38
circRNA17442	174.94	64.55	332.47	0	0	46.44	3.62	up	0.01	0.38
circRNA6083	249.91	167.82	83.12	0	0	46.44	3.43	up	0.01	0.38
circRNA4333	299.90	245.28	332.47	23.97	47.23	92.87	2.42	up	0.01	0.38
circRNA8374	149.95	90.37	62.34	0	0	0	inf	up	0.01	0.38
circRNA22423	149.95	38.73	145.45	0	0	0	inf	up	0.01	0.38
circRNA11327	149.95	38.73	145.45	0	0	0	inf	up	0.01	0.38
circRNA2459	0	0	0	143.84	165.31	46.44	-inf	down	0.01	0.38
circRNA13306	124.96	154.91	41.56	0	0	0	inf	up	0.01	0.39
circRNA6395	274.91	284.01	270.13	71.92	0	69.66	2.55	up	0.01	0.40
circRNA6419	49.98	142.00	103.90	0	0	0	inf	up	0.01	0.40
circRNA2515	849.71	568.02	415.58	167.81	0	162.53	2.47	up	0.01	0.40
circRNA7484	249.91	142.00	124.68	23.97	0	46.44	2.88	up	0.01	0.40
circRNA6824	74.97	129.10	83.12	0	0	0	inf	up	0.01	0.40
circRNA1046	3023.96	2323.71	1787.01	551.37	495.93	1114.49	1.72	up	0.01	0.40
ciRNA543	224.92	245.28	124.68	0	0	69.66	3.09	up	0.01	0.40
circRNA5277	124.96	77.46	83.12	0	0	0	inf	up	0.01	0.41
circRNA7583	624.79	271.10	519.48	143.84	47.23	139.31	2.10	up	0.01	0.41
circRNA11760	74.97	193.64	103.90	0	0	23.22	4.00	up	0.01	0.41
circRNA4104	0	0	0	119.86	94.46	92.87	-inf	down	0.01	0.42
circRNA16522	124.96	167.82	145.45	0	0	46.44	3.24	up	0.01	0.42
circRNA8418	124.96	64.55	103.90	0	0	0	inf	up	0.01	0.42
circRNA11894	299.90	193.64	187.01	0	70.85	46.44	2.54	up	0.01	0.42
circRNA2392	1699.42	1329.68	1703.90	647.26	448.70	557.24	1.52	up	0.01	0.42

circRNA5940	124.96	154.91	145.45	0	0	46.44	3.20	up	0.01	0.42
circRNA3559	149.95	180.73	145.45	0	23.62	46.44	2.76	up	0.01	0.43
circRNA16344	74.97	180.73	166.23	23.97	23.62	0	3.15	up	0.01	0.43
circRNA5917	374.87	219.46	332.47	0	70.85	92.87	2.50	up	0.01	0.44
circRNA3021	324.89	0	166.23	0	0	0	inf	up	0.01	0.44
circRNA16676	74.97	142.00	62.34	0	0	0	inf	up	0.01	0.45
circRNA24668	0	0	0	71.92	141.70	92.87	-inf	down	0.01	0.45
circRNA13676	124.96	51.64	124.68	0	0	0	inf	up	0.01	0.45
circRNA14614	199.93	90.37	83.12	0	0	23.22	4.01	up	0.01	0.45
circRNA11061	249.91	335.65	187.01	0	94.46	46.44	2.45	up	0.01	0.45
circRNA12065	74.97	116.19	83.12	0	0	0	inf	up	0.01	0.45
circRNA1120	224.92	142.00	187.01	71.92	0	0	2.95	up	0.01	0.46
circRNA10136	0	0	0	191.78	283.39	0	-inf	down	0.01	0.46
circRNA4167	0	0	0	335.62	141.70	0	-inf	down	0.01	0.46
circRNA45	0	0	0	335.62	141.70	0	-inf	down	0.01	0.46
circRNA16971	349.88	25.82	540.26	0	0	69.66	3.72	up	0.01	0.47
circRNA8375	349.88	116.19	353.25	0	70.85	69.66	2.54	up	0.01	0.47
circRNA16986	49.98	154.91	83.12	0	0	0	inf	up	0.01	0.47
circRNA9390	249.91	232.37	228.57	47.95	0	69.66	2.60	up	0.01	0.47
circRNA24384	49.98	142.00	83.12	0	0	0	inf	up	0.01	0.47
circRNA9789	99.97	51.64	145.45	0	0	0	inf	up	0.01	0.47
circRNA3262	724.75	361.47	436.36	0	0	185.75	3.04	up	0.01	0.47
circRNA13702	174.94	64.55	62.34	0	0	0	inf	up	0.01	0.47
circRNA6362	224.92	309.83	145.45	0	94.46	0	2.85	up	0.01	0.48
circRNA10274	174.94	0	270.13	0	0	0	inf	up	0.01	0.48
circRNA20451	74.97	129.10	62.34	0	0	0	inf	up	0.01	0.49
circRNA6384	974.67	490.56	290.91	167.81	165.31	116.09	1.97	up	0.01	0.49
circRNA23052	74.97	129.10	62.34	0	0	0	inf	up	0.01	0.49
circRNA1361	24.99	0	0	95.89	165.31	116.09	-3.92	down	0.01	0.50
circRNA13411	199.93	142.00	166.23	0	0	69.66	2.87	up	0.01	0.50
circRNA23278	124.96	38.73	124.68	0	0	0	inf	up	0.01	0.50
circRNA17255	124.96	38.73	124.68	0	0	0	inf	up	0.01	0.50
ciRNA117	0	0	0	143.84	118.08	46.44	-inf	down	0.01	0.50
circRNA3584	0	38.73	0	215.75	141.70	116.09	-3.61	down	0.01	0.50
circRNA2110	0	25.82	0	191.78	188.93	46.44	-4.05	down	0.01	0.51
circRNA5274	0	0	0	0	259.77	185.75	-inf	down	0.01	0.51
circRNA548	799.73	413.10	561.04	191.78	212.54	139.31	1.71	up	0.01	0.51
circRNA22783	74.97	77.46	103.90	0	0	0	inf	up	0.01	0.51
circRNA3132	0	25.82	0	95.89	118.08	162.53	-3.87	down	0.01	0.51
ciRNA854	49.98	77.46	145.45	0	0	0	inf	up	0.01	0.51
circRNA2595	99.97	116.19	311.69	23.97	47.23	0	2.89	up	0.01	0.51

circRNA24687	224.92	38.73	62.34	0	0	0	inf	up	0.01	0.51
circRNA3735	49.98	25.82	0	143.84	94.46	301.84	-2.83	down	0.01	0.51
circRNA4932	824.72	322.74	187.01	95.89	118.08	92.87	2.12	up	0.01	0.51
circRNA5639	149.95	167.82	228.57	23.97	0	69.66	2.54	up	0.01	0.51
circRNA3219	174.94	38.73	83.12	0	0	0	inf	up	0.01	0.51
circRNA3813	149.95	90.37	62.34	311.65	377.85	534.02	-2.02	down	0.01	0.51
circRNA13318	99.97	90.37	62.34	0	0	0	inf	up	0.01	0.51
circRNA1445	99.97	90.37	62.34	0	0	0	inf	up	0.01	0.51
circRNA4124	49.98	0	41.56	119.86	354.24	162.53	-2.80	down	0.01	0.51
circRNA2027	224.92	154.91	83.12	0	47.23	23.22	2.72	up	0.01	0.51
circRNA7781	574.80	348.56	228.57	95.89	47.23	139.31	2.03	up	0.01	0.51
circRNA5900	124.96	116.19	166.23	0	47.23	0	3.11	up	0.01	0.53
circRNA2705	49.98	51.64	20.78	167.81	212.54	325.06	-2.53	down	0.01	0.53
circRNA17359	124.96	116.19	166.23	47.95	0	0	3.09	up	0.01	0.53
circRNA3999	199.93	103.28	249.35	0	47.23	46.44	2.56	up	0.01	0.55
circRNA3634	0	0	0	71.92	141.70	69.66	-inf	down	0.01	0.55
circRNA11102	0	38.73	0	119.86	165.31	162.53	-3.53	down	0.01	0.55
circRNA14042	149.95	142.00	103.90	0	0	46.44	3.09	up	0.01	0.55
circRNA23890	74.97	142.00	41.56	0	0	0	inf	up	0.01	0.55
circRNA512	49.98	142.00	62.34	0	0	0	inf	up	0.01	0.55
circRNA22511	124.96	103.28	41.56	0	0	0	inf	up	0.01	0.55
circRNA7963	299.90	116.19	332.47	47.95	23.62	69.66	2.41	up	0.01	0.55
circRNA2124	999.66	1394.23	1059.74	407.54	472.32	394.71	1.44	up	0.01	0.55
circRNA2375	249.91	90.37	519.48	0	47.23	92.87	2.62	up	0.01	0.56
circRNA17137	324.89	245.28	249.35	71.92	0	92.87	2.31	up	0.01	0.57
circRNA2835	149.95	180.73	145.45	71.92	0	0	2.73	up	0.01	0.58
circRNA24310	149.95	77.46	41.56	0	0	0	inf	up	0.01	0.58
circRNA17625	99.97	193.64	103.90	47.95	0	0	3.05	up	0.01	0.59
circRNA2484	299.90	309.83	332.47	743.15	1157.18	1207.36	-1.72	down	0.01	0.59
circRNA3210	74.97	142.00	187.01	0	0	46.44	3.12	up	0.01	0.59
circRNA2179	1224.58	413.10	1288.31	71.92	283.39	394.71	1.96	up	0.01	0.59
ciRNA1250	74.97	25.82	207.79	0	0	0	inf	up	0.01	0.59
circRNA2604	99.97	219.46	124.68	47.95	0	23.22	2.64	up	0.01	0.59
circRNA11127	174.94	142.00	145.45	0	0	69.66	2.73	up	0.01	0.60
circRNA4264	74.97	25.82	62.34	311.65	188.93	301.84	-2.30	down	0.01	0.60
circRNA3318	174.94	180.73	0	0	0	0	inf	up	0.01	0.60
circRNA17143	124.96	90.37	0	215.75	283.39	789.43	-2.58	down	0.01	0.60
circRNA14940	74.97	77.46	83.12	0	0	0	inf	up	0.01	0.60
circRNA17664	149.95	154.91	83.12	0	0	46.44	3.06	up	0.01	0.60
circRNA2274	0	0	0	71.92	94.46	92.87	-inf	down	0.01	0.60
ciRNA74	0	0	0	167.81	236.16	0	-inf	down	0.01	0.60

circRNA17389	124.96	64.55	62.34	0	0	0	inf	up	0.01	0.60
circRNA5580	174.94	116.19	103.90	0	0	46.44	3.09	up	0.01	0.60
circRNA2566	74.97	258.19	83.12	0	0	46.44	3.16	up	0.01	0.60
circRNA13884	99.97	51.64	103.90	0	0	0	inf	up	0.01	0.60
circRNA4203	174.94	116.19	103.90	0	0	46.44	3.09	up	0.01	0.60
circRNA14130	149.95	103.28	228.57	0	0	69.66	2.79	up	0.02	0.61
circRNA2424	149.95	193.64	0	0	0	0	inf	up	0.02	0.61
circRNA8379	49.98	51.64	62.34	239.73	354.24	185.75	-2.25	down	0.02	0.61
circRNA51906	0	0	0	0	259.77	139.31	-inf	down	0.02	0.61
circRNA16918	74.97	90.37	62.34	0	0	0	inf	up	0.02	0.61
circRNA14229	74.97	90.37	62.34	0	0	0	inf	up	0.02	0.61
circRNA2923	824.72	503.47	623.38	215.75	0	208.97	2.20	up	0.02	0.62
circRNA2504	274.91	309.83	207.79	71.92	70.85	69.66	1.90	up	0.02	0.62
circRNA8194	49.98	167.82	41.56	0	0	0	inf	up	0.02	0.62
circRNA17256	49.98	167.82	41.56	0	0	0	inf	up	0.02	0.62
circRNA8158	224.92	38.73	41.56	0	0	0	inf	up	0.02	0.62
circRNA2148	74.97	0	103.90	407.54	283.39	208.97	-2.33	down	0.02	0.62
circRNA1125	574.80	632.57	498.70	0	188.93	208.97	2.10	up	0.02	0.63
circRNA709	0	64.55	103.90	311.65	377.85	255.40	-2.49	down	0.02	0.63
circRNA7025	49.98	322.74	207.79	0	47.23	46.44	2.63	up	0.02	0.64
circRNA23021	124.96	25.82	124.68	0	0	0	inf	up	0.02	0.64
circRNA16898	149.95	25.82	103.90	0	0	0	inf	up	0.02	0.64
circRNA23752	74.97	51.64	124.68	0	0	0	inf	up	0.02	0.64
circRNA17317	149.95	51.64	62.34	0	0	0	inf	up	0.02	0.64
circRNA4800	199.93	271.10	124.68	0	94.46	0	2.66	up	0.02	0.64
circRNA13808	249.91	103.28	124.68	71.92	0	0	2.73	up	0.02	0.64
circRNA3838	99.97	129.10	103.90	431.51	637.63	278.62	-2.02	down	0.02	0.64
circRNA6076	174.94	180.73	103.90	0	0	69.66	2.72	up	0.02	0.64
circRNA22805	124.96	38.73	83.12	0	0	0	inf	up	0.02	0.65
circRNA23250	99.97	38.73	103.90	0	0	0	inf	up	0.02	0.65
ciRNA533	224.92	284.01	311.69	71.92	70.85	92.87	1.80	up	0.02	0.65
circRNA16503	0	0	0	47.95	94.46	116.09	-inf	down	0.02	0.65
circRNA3227	49.98	38.73	124.68	359.59	283.39	255.40	-2.07	down	0.02	0.66
circRNA47487	0	0	0	95.89	47.23	116.09	-inf	down	0.02	0.66
circRNA13421	249.91	142.00	166.23	47.95	70.85	0	2.23	up	0.02	0.68
circRNA8393	99.97	116.19	290.91	71.92	0	0	2.82	up	0.02	0.69
circRNA14435	149.95	154.91	270.13	47.95	23.62	46.44	2.28	up	0.02	0.69
circRNA10188	0	335.65	166.23	0	23.62	23.22	3.42	up	0.02	0.69
circRNA16660	199.93	142.00	103.90	0	0	69.66	2.68	up	0.02	0.69
circRNA760	699.76	374.38	935.07	311.65	118.08	208.97	1.65	up	0.02	0.69
circRNA9156	74.97	38.73	124.68	0	0	0	inf	up	0.02	0.69

circRNA380	699.76	864.94	1475.33	359.59	472.32	92.87	1.72	up	0.02	0.69
circRNA16994	74.97	38.73	124.68	0	0	0	inf	up	0.02	0.69
circRNA14038	74.97	116.19	41.56	0	0	0	inf	up	0.02	0.69
circRNA26020	74.97	116.19	41.56	0	0	0	inf	up	0.02	0.69
circRNA3854	274.91	232.37	290.91	71.92	70.85	92.87	1.76	up	0.02	0.69
circRNA3128	99.97	64.55	62.34	191.78	259.77	510.81	-2.08	down	0.02	0.69
circRNA5151	99.97	258.19	166.23	23.97	70.85	23.22	2.15	up	0.02	0.69
circRNA9865	0	0	0	47.95	141.70	69.66	-inf	down	0.02	0.69
circRNA8556	224.92	180.73	270.13	0	0	116.09	2.54	up	0.02	0.69
circRNA24954	99.97	64.55	62.34	0	0	0	inf	up	0.02	0.70
circRNA27401	99.97	64.55	62.34	0	0	0	inf	up	0.02	0.70
circRNA8828	124.96	51.64	145.45	23.97	0	0	3.75	up	0.02	0.70
circRNA813	199.93	77.46	124.68	527.40	779.32	232.18	-1.94	down	0.02	0.71
circRNA11200	0	25.82	0	143.84	47.23	162.53	-3.78	down	0.02	0.72
circRNA13432	49.98	51.64	145.45	0	0	0	inf	up	0.02	0.72
circRNA16646	149.95	77.46	145.45	0	47.23	0	2.98	up	0.02	0.73
circRNA5541	99.97	348.56	41.56	47.95	0	23.22	2.78	up	0.02	0.73
circRNA18541	74.97	51.64	103.90	0	0	0	inf	up	0.02	0.73
circRNA9898	0	0	0	95.89	70.85	69.66	-inf	down	0.02	0.73
circRNA23501	0	0	0	95.89	70.85	69.66	-inf	down	0.02	0.73
circRNA4032	0	77.46	0	167.81	165.31	278.62	-2.98	down	0.02	0.73
circRNA6323	474.84	232.37	290.91	95.89	141.70	0	2.07	up	0.02	0.75
circRNA6025	124.96	167.82	124.68	263.70	779.32	557.24	-1.94	down	0.02	0.76
circRNA22762	49.98	0	0	215.75	188.93	46.44	-3.17	down	0.02	0.76
circRNA352	549.81	116.19	187.01	47.95	70.85	69.66	2.18	up	0.02	0.76
circRNA7505	199.93	0	145.45	0	0	0	inf	up	0.02	0.76
circRNA10244	99.97	77.46	41.56	215.75	212.54	534.02	-2.14	down	0.02	0.76
circRNA14626	199.93	0	145.45	0	0	0	inf	up	0.02	0.76
circRNA14110	99.97	77.46	41.56	0	0	0	inf	up	0.02	0.76
circRNA13924	49.98	77.46	83.12	0	0	0	inf	up	0.02	0.76
circRNA17102	99.97	77.46	41.56	0	0	0	inf	up	0.02	0.76
circRNA7376	99.97	77.46	103.90	0	0	23.22	3.60	up	0.02	0.76
circRNA4056	224.92	142.00	166.23	0	0	92.87	2.52	up	0.02	0.76
circRNA808	1849.37	1329.68	1059.74	671.24	519.55	580.46	1.26	up	0.02	0.76
circRNA2475	349.88	38.73	228.57	0	70.85	23.22	2.71	up	0.02	0.76
circRNA23416	49.98	64.55	103.90	0	0	0	inf	up	0.02	0.76
circRNA23389	49.98	64.55	103.90	0	0	0	inf	up	0.02	0.76
circRNA16923	49.98	64.55	103.90	0	0	0	inf	up	0.02	0.76
circRNA17345	174.94	38.73	41.56	0	0	0	inf	up	0.02	0.76
circRNA5911	49.98	38.73	145.45	0	0	0	inf	up	0.02	0.76
circRNA20565	49.98	129.10	41.56	0	0	0	inf	up	0.02	0.76

circRNA478	149.95	90.37	145.45	23.97	0	46.44	2.45	up	0.02	0.76
circRNA8095	824.72	245.28	581.82	191.78	0	185.75	2.13	up	0.02	0.76
circRNA50335	0	0	0	95.89	47.23	92.87	-inf	down	0.03	0.76
circRNA3141	0	0	0	263.70	0	92.87	-inf	down	0.03	0.77
circRNA15727	74.97	90.37	166.23	23.97	23.62	0	2.80	up	0.03	0.77
circRNA6080	74.97	103.28	41.56	0	0	0	inf	up	0.03	0.77
circRNA16915	174.94	25.82	62.34	0	0	0	inf	up	0.03	0.77
circRNA22273	49.98	103.28	62.34	0	0	0	inf	up	0.03	0.77
circRNA22653	74.97	103.28	41.56	0	0	0	inf	up	0.03	0.77
circRNA22658	49.98	103.28	62.34	0	0	0	inf	up	0.03	0.77
circRNA16661	74.97	103.28	41.56	0	0	0	inf	up	0.03	0.77
circRNA2834	174.94	103.28	124.68	23.97	47.23	23.22	2.09	up	0.03	0.77
circRNA6111	149.95	77.46	0	359.59	330.62	325.06	-2.16	down	0.03	0.77
circRNA2452	74.97	38.73	103.90	0	0	0	inf	up	0.03	0.77
circRNA16376	49.98	90.37	62.34	0	0	0	inf	up	0.03	0.77
circRNA4083	49.98	90.37	62.34	0	0	0	inf	up	0.03	0.77
circRNA10750	274.91	232.37	187.01	47.95	47.23	92.87	1.88	up	0.03	0.77
circRNA42386	0	0	0	119.86	70.85	46.44	-inf	down	0.03	0.78
circRNA16922	224.92	206.55	145.45	0	94.46	46.44	2.03	up	0.03	0.78
circRNA10064	0	154.91	124.68	0	0	0	inf	up	0.03	0.78
circRNA7962	124.96	90.37	62.34	0	23.62	0	3.56	up	0.03	0.78
circRNA8067	199.93	116.19	0	0	0	0	inf	up	0.03	0.78
ciRNA455	49.98	258.19	0	0	0	0	inf	up	0.03	0.79
circRNA939	499.83	400.19	353.25	0	141.70	185.75	1.94	up	0.03	0.79
circRNA4274	174.94	142.00	187.01	95.89	0	0	2.39	up	0.03	0.79
circRNA6814	24.99	167.82	41.56	0	0	0	inf	up	0.03	0.79
circRNA1253	124.96	90.37	124.68	0	0	46.44	2.87	up	0.03	0.79
circRNA8440	149.95	142.00	0	0	0	0	inf	up	0.03	0.80
circRNA16418	0	38.73	0	71.92	188.93	139.31	-3.37	down	0.03	0.80
circRNA6702	249.91	193.64	103.90	0	94.46	23.22	2.22	up	0.03	0.81
ciRNA293	0	0	0	191.78	141.70	0	-inf	down	0.03	0.81
circRNA7805	0	38.73	0	95.89	212.54	92.87	-3.37	down	0.03	0.81
circRNA2047	0	38.73	0	191.78	0	417.93	-3.98	down	0.03	0.81
circRNA7527	74.97	64.55	62.34	0	0	0	inf	up	0.03	0.81
circRNA24146	74.97	64.55	62.34	0	0	0	inf	up	0.03	0.81
circRNA13518	99.97	116.19	124.68	47.95	0	0	2.83	up	0.03	0.81
circRNA13921	0	193.64	83.12	0	0	0	inf	up	0.03	0.81
circRNA5111	0	0	0	71.92	23.62	162.53	-inf	down	0.03	0.82
ciRNA820	99.97	51.64	62.34	0	0	0	inf	up	0.03	0.82
circRNA17281	99.97	51.64	62.34	0	0	0	inf	up	0.03	0.82
circRNA8321	599.79	258.19	498.70	143.84	94.46	208.97	1.60	up	0.03	0.83

circRNA24959	99.97	90.37	145.45	0	47.23	0	2.83	up	0.03	0.83
circRNA1400	249.91	219.46	270.13	71.92	70.85	92.87	1.65	up	0.03	0.83
circRNA398	249.91	90.37	124.68	71.92	23.62	0	2.28	up	0.03	0.83
circRNA2734	199.93	219.46	228.57	47.95	94.46	46.44	1.78	up	0.03	0.83
circRNA16547	0	77.46	207.79	0	0	0	inf	up	0.03	0.83
circRNA3252	249.91	77.46	0	0	0	0	inf	up	0.03	0.83
circRNA4418	124.96	64.55	0	167.81	212.54	557.24	-2.31	down	0.03	0.83
circRNA10876	149.95	103.28	145.45	0	70.85	0	2.49	up	0.03	0.83
circRNA360	0	38.73	0	167.81	118.08	92.87	-3.29	down	0.03	0.83
circRNA22621	149.95	38.73	41.56	0	0	0	inf	up	0.03	0.83
ciRNA2643	0	0	0	239.73	94.46	0	-inf	down	0.03	0.83
circRNA14153	99.97	64.55	41.56	0	0	0	inf	up	0.03	0.83
circRNA8102	74.97	38.73	83.12	0	0	0	inf	up	0.03	0.83
circRNA23782	74.97	38.73	83.12	0	0	0	inf	up	0.03	0.83
circRNA25033	49.98	64.55	83.12	0	0	0	inf	up	0.03	0.83
circRNA5888	49.98	64.55	83.12	0	0	0	inf	up	0.03	0.83
circRNA15070	74.97	38.73	83.12	0	0	0	inf	up	0.03	0.83
circRNA13755	74.97	38.73	83.12	0	0	0	inf	up	0.03	0.83
circRNA23791	49.98	64.55	83.12	0	0	0	inf	up	0.03	0.83
circRNA16802	99.97	38.73	62.34	0	0	0	inf	up	0.03	0.83
circRNA13118	0	64.55	228.57	0	0	0	inf	up	0.03	0.83
circRNA16683	49.98	77.46	62.34	0	0	0	inf	up	0.03	0.83
circRNA3346	49.98	77.46	62.34	0	0	0	inf	up	0.03	0.83
circRNA29619	49.98	77.46	62.34	0	0	0	inf	up	0.03	0.83
circRNA24938	74.97	77.46	41.56	0	0	0	inf	up	0.03	0.83
circRNA3906	424.85	464.74	166.23	95.89	165.31	0	2.02	up	0.03	0.83
circRNA1211	0	0	0	71.92	47.23	92.87	-inf	down	0.03	0.83
circRNA2132	0	12.91	0	71.92	283.39	23.22	-4.87	down	0.03	0.83
circRNA13385	0	0	0	95.89	70.85	46.44	-inf	down	0.03	0.83
circRNA11493	174.94	90.37	103.90	47.95	23.62	0	2.37	up	0.03	0.83
circRNA9907	0	0	0	95.89	47.23	69.66	-inf	down	0.03	0.83
circRNA1991	99.97	193.64	103.90	0	70.85	0	2.49	up	0.03	0.83
circRNA2694	0	0	0	311.65	0	46.44	-inf	down	0.03	0.83
circRNA12515	0	206.55	62.34	0	0	0	inf	up	0.03	0.83
circRNA4113	574.80	142.00	249.35	71.92	0	139.31	2.19	up	0.03	0.83
circRNA603	49.98	25.82	83.12	311.65	425.09	69.66	-2.34	down	0.03	0.83
circRNA4048	149.95	129.10	62.34	0	47.23	0	2.85	up	0.03	0.83
circRNA240	0	129.10	124.68	0	0	0	inf	up	0.03	0.84
circRNA17336	0	129.10	124.68	0	0	0	inf	up	0.03	0.84
circRNA18243	674.77	322.74	249.35	0	0	208.97	2.58	up	0.03	0.85
circRNA582	49.98	77.46	0	311.65	259.77	92.87	-2.38	down	0.03	0.85

circRNA13263	124.96	51.64	41.56	0	0	0	inf	up	0.03	0.86
circRNA8376	0	0	0	143.84	165.31	0	-inf	down	0.04	0.86
circRNA5989	224.92	116.19	83.12	0	70.85	0	2.58	up	0.04	0.86
circRNA13436	99.97	284.01	228.57	0	0	116.09	2.40	up	0.04	0.86
circRNA3055	124.96	38.73	0	287.67	212.54	185.75	-2.07	down	0.04	0.87
circRNA8453	349.88	271.10	332.47	95.89	141.70	116.09	1.43	up	0.04	0.87
circRNA5064	124.96	51.64	103.90	0	0	23.22	3.59	up	0.04	0.87
circRNA11135	0	0	0	0	188.93	116.09	-inf	down	0.04	0.87
circRNA8331	474.84	258.19	290.91	71.92	0	185.75	1.99	up	0.04	0.88
circRNA1928	224.92	129.10	124.68	0	94.46	0	2.34	up	0.04	0.88
circRNA16419	24.99	180.73	20.78	0	0	0	inf	up	0.04	0.88
circRNA730	99.97	167.82	0	0	0	0	inf	up	0.04	0.88
circRNA8278	124.96	90.37	103.90	0	0	46.44	2.78	up	0.04	0.88
circRNA3096	149.95	0	145.45	0	0	0	inf	up	0.04	0.88
circRNA2972	0	0	0	47.95	118.08	46.44	-inf	down	0.04	0.88
circRNA4285	0	0	0	47.95	118.08	46.44	-inf	down	0.04	0.88
circRNA11046	174.94	116.19	187.01	0	0	92.87	2.36	up	0.04	0.88
circRNA13357	49.98	90.37	41.56	0	0	0	inf	up	0.04	0.88
circRNA4220	124.96	142.00	290.91	71.92	0	46.44	2.24	up	0.04	0.88
circRNA27150	49.98	38.73	103.90	0	0	0	inf	up	0.04	0.88
circRNA23816	124.96	38.73	41.56	0	0	0	inf	up	0.04	0.88
circRNA13504	124.96	116.19	83.12	0	0	46.44	2.80	up	0.04	0.88
circRNA24459	124.96	38.73	41.56	0	0	0	inf	up	0.04	0.88
circRNA5496	99.97	64.55	187.01	0	0	46.44	2.92	up	0.04	0.88
circRNA23575	99.97	25.82	83.12	0	0	0	inf	up	0.04	0.88
circRNA14407	99.97	25.82	83.12	0	0	0	inf	up	0.04	0.88
circRNA3483	49.98	0	41.56	167.81	188.93	92.87	-2.30	down	0.04	0.88
ciRNA3572	0	0	0	215.75	94.46	0	-inf	down	0.04	0.88
circRNA13733	99.97	25.82	83.12	0	0	0	inf	up	0.04	0.88
circRNA13656	99.97	25.82	83.12	0	0	0	inf	up	0.04	0.88
circRNA32485	199.93	0	103.90	0	0	0	inf	up	0.04	0.88
circRNA26725	0	0	0	119.86	47.23	46.44	-inf	down	0.04	0.88
circRNA8096	0	0	41.56	143.84	118.08	92.87	-3.09	down	0.04	0.88
circRNA6458	49.98	25.82	0	47.95	401.47	116.09	-2.90	down	0.04	0.88
circRNA956	949.67	722.93	727.27	407.54	401.47	255.40	1.17	up	0.04	0.88
circRNA5235	299.90	90.37	83.12	0	70.85	23.22	2.33	up	0.04	0.88
circRNA13845	74.97	193.64	0	0	0	0	inf	up	0.04	0.88
circRNA12569	174.94	25.82	41.56	0	0	0	inf	up	0.04	0.88
circRNA81	449.85	167.82	187.01	0	0	139.31	2.53	up	0.04	0.88
circRNA3396	0	25.82	41.56	95.89	212.54	92.87	-2.57	down	0.04	0.89
circRNA3692	124.96	77.46	145.45	167.81	307.01	859.08	-1.94	down	0.04	0.90

circRNA17275	0	180.73	62.34	0	0	0	inf	up	0.04	0.90
circRNA17547	74.97	180.73	0	0	0	0	inf	up	0.04	0.90
circRNA13445	74.97	142.00	41.56	0	23.62	0	3.45	up	0.04	0.90
circRNA2693	324.89	335.65	249.35	191.78	70.85	23.22	1.67	up	0.04	0.90
circRNA325	199.93	180.73	103.90	47.95	23.62	46.44	2.04	up	0.04	0.90
circRNA3686	149.95	180.73	62.34	551.37	307.01	487.59	-1.78	down	0.04	0.90
circRNA3398	0	0	0	0	283.39	46.44	-inf	down	0.04	0.90
circRNA11025	99.97	129.10	83.12	0	0	46.44	2.75	up	0.04	0.90
circRNA3926	0	0	0	239.73	0	69.66	-inf	down	0.04	0.90
circRNA7251	49.98	64.55	228.57	23.97	23.62	0	2.85	up	0.04	0.90
circRNA3356	49.98	0	0	95.89	94.46	162.53	-2.82	down	0.04	0.90
circRNA12531	124.96	103.28	41.56	0	23.62	0	3.51	up	0.04	0.90
circRNA6363	249.91	258.19	249.35	95.89	0	116.09	1.84	up	0.04	0.90
circRNA3567	0	25.82	41.56	0	377.85	301.84	-3.33	down	0.04	0.90
circRNA3143	49.98	0	0	167.81	94.46	92.87	-2.83	down	0.04	0.90
circRNA10357	74.97	129.10	83.12	23.97	0	23.22	2.61	up	0.04	0.90
circRNA1821	99.97	154.91	124.68	263.70	543.17	441.15	-1.72	down	0.04	0.90
circRNA2585	49.98	51.64	83.12	0	0	0	inf	up	0.04	0.90
circRNA15308	99.97	51.64	41.56	0	0	0	inf	up	0.04	0.90
circRNA22643	99.97	51.64	41.56	0	0	0	inf	up	0.04	0.90
circRNA23886	99.97	51.64	41.56	0	0	0	inf	up	0.04	0.90
circRNA16771	99.97	51.64	41.56	0	0	0	inf	up	0.04	0.90
circRNA20168	49.98	51.64	83.12	0	0	0	inf	up	0.04	0.90
circRNA13917	49.98	51.64	83.12	0	0	0	inf	up	0.04	0.90
circRNA14028	49.98	51.64	83.12	0	0	0	inf	up	0.04	0.90
circRNA6297	849.71	580.93	685.71	0	188.93	371.50	1.92	up	0.04	0.90
circRNA6226	149.95	64.55	311.69	0	47.23	69.66	2.17	up	0.04	0.90
circRNA4012	74.97	64.55	41.56	0	0	0	inf	up	0.04	0.90
circRNA18755	74.97	64.55	41.56	0	0	0	inf	up	0.04	0.90
circRNA17016	74.97	64.55	41.56	0	0	0	inf	up	0.04	0.90
circRNA3098	224.92	142.00	103.90	599.32	637.63	255.40	-1.66	down	0.04	0.90
circRNA2330	1474.49	619.66	2846.76	647.26	377.85	766.21	1.46	up	0.04	0.90
circRNA3166	0	0	0	71.92	70.85	46.44	-inf	down	0.04	0.90
circRNA11269	124.96	64.55	20.78	0	0	0	inf	up	0.04	0.91
circRNA16573	99.97	154.91	0	0	0	0	inf	up	0.04	0.91
circRNA3774	199.93	38.73	124.68	47.95	0	0	2.92	up	0.04	0.91
circRNA7024	149.95	232.37	145.45	47.95	70.85	23.22	1.89	up	0.04	0.91
circRNA10350	74.97	90.37	20.78	0	0	0	inf	up	0.04	0.91
circRNA1631	549.81	387.29	498.70	2948.64	1109.95	557.24	-1.68	down	0.04	0.92
circRNA2761	124.96	103.28	187.01	23.97	23.62	69.66	1.82	up	0.04	0.92
circRNA8473	0	0	0	143.84	141.70	0	-inf	down	0.05	0.92

circRNA428	1199.59	555.11	581.82	263.70	472.32	116.09	1.46	up	0.05	0.92
circRNA9862	0	0	0	143.84	141.70	0	-inf	down	0.05	0.92
circRNA2866	0	77.46	0	167.81	94.46	255.40	-2.74	down	0.05	0.92
circRNA488	49.98	25.82	41.56	119.86	283.39	139.31	-2.21	down	0.05	0.92
circRNA3681	299.90	413.10	166.23	863.02	1487.80	510.81	-1.70	down	0.05	0.92
circRNA921	1299.55	1265.13	706.49	215.75	425.09	673.34	1.32	up	0.05	0.92
circRNA16292	74.97	103.28	20.78	0	0	0	inf	up	0.05	0.93
circRNA4664	574.80	335.65	394.81	215.75	165.31	139.31	1.33	up	0.05	0.93
circRNA107	49.98	0	20.78	167.81	118.08	92.87	-2.42	down	0.05	0.93
circRNA16782	49.98	25.82	124.68	0	0	0	inf	up	0.05	0.93
circRNA13469	149.95	25.82	41.56	0	0	0	inf	up	0.05	0.93
ciRNA790	49.98	25.82	124.68	0	0	0	inf	up	0.05	0.93
circRNA24255	149.95	25.82	41.56	0	0	0	inf	up	0.05	0.93
circRNA13698	0	245.28	20.78	0	0	0	inf	up	0.05	0.93
circRNA3794	49.98	0	41.56	119.86	188.93	116.09	-2.21	down	0.05	0.93
circRNA13655	99.97	193.64	83.12	71.92	0	0	2.39	up	0.05	0.93
circRNA5992	224.92	103.28	124.68	0	0	92.87	2.29	up	0.05	0.93
circRNA5295	399.86	258.19	103.90	23.97	0	139.31	2.22	up	0.05	0.93
circRNA1753	124.96	64.55	103.90	0	23.62	23.22	2.65	up	0.05	0.93
circRNA2714	99.97	77.46	207.79	23.97	23.62	46.44	2.03	up	0.05	0.94
circRNA17040	99.97	38.73	41.56	0	0	0	inf	up	0.05	0.94
circRNA2977	0	0	0	0	259.77	46.44	-inf	down	0.05	0.95
circRNA15895	0	0	0	95.89	47.23	46.44	-inf	down	0.05	0.95
circRNA22290	174.94	0	103.90	0	0	0	inf	up	0.05	0.95
circRNA4921	74.97	129.10	41.56	0	23.62	0	3.38	up	0.05	0.95
circRNA23168	149.95	142.00	83.12	47.95	47.23	0	1.98	up	0.05	0.96
circRNA3221	99.97	38.73	83.12	287.67	236.16	185.75	-1.68	down	0.05	0.96

Table S4 complete DECs lists (Som vs Stm)

Accession	Som1_srpbm	Som2_srpbm	Som3_srpbm	Stm1_srpbm	Stm3_srpbm	Stm2_srpbm	log_2FC	regulation	pvalue	qvalue
circRNA9931	0	0	0	132.90	442.60	1896.77	-inf	down	0.00	0.11
circRNA4250	287.60	454.71	367.44	0	0	0	inf	up	0.00	0.11
circRNA9747	0	0	0	903.73	590.13	129.33	-inf	down	0.00	0.11
circRNA9976	0	0	0	53.16	553.24	2543.39	-inf	down	0.00	0.11
ciRNA153	156.87	294.23	452.23	0	0	0	inf	up	0.00	0.25
circRNA4035	313.75	133.74	395.70	0	0	0	inf	up	0.00	0.27
circRNA3297	52.29	0	84.79	79.74	2323.63	4397.05	-5.63	down	0.00	0.27
circRNA9925	0	0	0	159.48	258.18	258.65	-inf	down	0.00	0.27
circRNA10101	0	0	0	132.90	147.53	409.53	-inf	down	0.00	0.35
circRNA5467	0	26.75	0	797.41	442.60	107.77	-5.66	down	0.00	0.35
circRNA2170	183.02	267.48	197.85	0	0	0	inf	up	0.00	0.35

circRNA10964	0	0	0	0	737.66	1379.47	-inf	down	0.00	0.37
circRNA8978	0	0	0	345.54	0	1056.16	-inf	down	0.00	0.59
circRNA9835	0	0	0	159.48	0	1918.32	-inf	down	0.00	0.59
circRNA4787	0	588.45	791.40	0	0	0	inf	up	0.00	0.59
circRNA4001	183.02	106.99	226.12	0	0	0	inf	up	0.00	0.65
circRNA3013	183.02	213.98	339.17	0	0	43.11	4.09	up	0.00	0.65
circRNA3071	156.87	106.99	254.38	0	0	0	inf	up	0.00	0.65
circRNA3752	156.87	133.74	197.85	0	0	0	inf	up	0.00	0.65
circRNA8910	0	0	0	212.64	36.88	344.87	-inf	down	0.00	0.65
circRNA11154	0	0	0	0	295.06	1034.60	-inf	down	0.00	0.65
circRNA2275	130.73	133.74	197.85	0	0	0	inf	up	0.00	0.68
circRNA10097	0	0	0	79.74	110.65	280.20	-inf	down	0.00	0.68
circRNA9987	0	0	0	398.70	442.60	0	-inf	down	0.00	0.68
circRNA7846	0	0	28.26	1196.11	442.60	0	-5.86	down	0.00	0.68
circRNA8775	0	0	0	265.80	627.01	0	-inf	down	0.00	0.68
circRNA10086	0	0	0	611.35	258.18	0	-inf	down	0.00	0.68
circRNA8412	0	0	84.79	956.89	553.24	107.77	-4.25	down	0.00	0.68
circRNA1402	287.60	347.72	367.44	0	0	86.22	3.54	up	0.01	0.87
circRNA11147	0	0	0	0	184.41	775.95	-inf	down	0.01	0.87
circRNA9958	0	0	0	292.38	368.83	0	-inf	down	0.01	0.93
circRNA2110	156.87	53.50	254.38	0	0	0	inf	up	0.01	0.93
circRNA9772	0	0	0	239.22	405.71	0	-inf	down	0.01	0.93
circRNA9014	0	0	0	106.32	73.77	215.54	-inf	down	0.01	0.93
circRNA9540	0	0	0	531.60	147.53	0	-inf	down	0.01	0.93
circRNA3938	130.73	106.99	169.59	0	0	0	inf	up	0.01	0.93
circRNA3560	130.73	106.99	169.59	0	0	0	inf	up	0.01	0.93
circRNA3805	183.02	53.50	197.85	0	0	0	inf	up	0.01	0.94
ciRNA79	156.87	80.24	169.59	0	0	0	inf	up	0.01	0.94
circRNA3947	78.44	0	0	212.64	221.30	495.75	-3.57	down	0.01	0.94
ciRNA142	104.58	106.99	197.85	0	0	0	inf	up	0.01	0.94
circRNA2947	156.87	53.50	226.12	0	0	0	inf	up	0.01	0.94
circRNA10051	0	0	0	505.02	110.65	0	-inf	down	0.01	0.98
circRNA3319	366.04	294.23	226.12	0	73.77	0	3.59	up	0.01	1
circRNA9795	0	0	0	159.48	0	409.53	-inf	down	0.01	1
circRNA2994	104.58	187.23	254.38	0	0	43.11	3.66	up	0.01	1
circRNA9834	0	0	0	159.48	110.65	86.22	-inf	down	0.01	1
circRNA28	444.48	187.23	254.38	0	0	86.22	3.36	up	0.01	1
circRNA3350	209.17	374.47	0	0	0	0	inf	up	0.01	1
circRNA351	287.60	80.24	56.53	0	0	0	inf	up	0.01	1
circRNA3855	339.89	374.47	367.44	0	0	129.33	3.06	up	0.01	1
circRNA9156	0	0	0	292.38	184.41	0	-inf	down	0.01	1

circRNA10017	0	0	0	79.74	73.77	215.54	-inf	down	0.01	1
circRNA3146	156.87	401.22	0	0	0	0	inf	up	0.01	1
circRNA3767	78.44	133.74	141.32	0	0	0	inf	up	0.01	1
circRNA10314	0	0	0	0	295.06	280.20	-inf	down	0.01	1
circRNA9912	0	0	0	106.32	147.53	86.22	-inf	down	0.01	1
circRNA9769	0	0	0	372.12	110.65	0	-inf	down	0.01	1
circRNA10055	0	0	0	79.74	184.41	86.22	-inf	down	0.01	1
circRNA9457	0	0	0	212.64	258.18	0	-inf	down	0.01	1
circRNA3032	104.58	106.99	113.06	0	0	0	inf	up	0.02	1
circRNA3173	78.44	187.23	84.79	0	0	0	inf	up	0.02	1
circRNA11108	0	0	0	0	147.53	409.53	-inf	down	0.02	1
circRNA9982	0	0	0	186.06	0	258.65	-inf	down	0.02	1
circRNA9697	0	0	0	239.22	184.41	0	-inf	down	0.02	1
circRNA1923	104.58	187.23	141.32	26.58	0	0	4.03	up	0.02	1
circRNA3162	183.02	80.24	84.79	0	0	0	inf	up	0.02	1
circRNA3396	183.02	80.24	84.79	0	0	0	inf	up	0.02	1
circRNA3246	104.58	53.50	0	584.77	553.24	107.77	-2.98	down	0.02	1
circRNA3476	287.60	53.50	56.53	0	0	0	inf	up	0.02	1
circRNA3361	78.44	133.74	197.85	1116.37	737.66	172.43	-2.31	down	0.02	1
circRNA10120	0	0	0	0	331.95	172.43	-inf	down	0.02	1
circRNA9946	0	0	0	186.06	221.30	0	-inf	down	0.02	1
circRNA8819	0	0	0	186.06	221.30	0	-inf	down	0.02	1
circRNA4242	183.02	133.74	113.06	0	0	43.11	3.32	up	0.02	1
circRNA9854	0	0	0	159.48	258.18	0	-inf	down	0.02	1
circRNA670	78.44	213.98	56.53	0	0	0	inf	up	0.02	1
circRNA1773	104.58	53.50	197.85	0	0	0	inf	up	0.02	1
circRNA9608	0	0	0	79.74	0	409.53	-inf	down	0.02	1
circRNA10080	0	0	0	132.90	295.06	0	-inf	down	0.02	1
circRNA1817	418.33	213.98	310.91	0	110.65	43.11	2.62	up	0.02	1
circRNA1897	104.58	0	84.79	584.77	258.18	215.54	-2.48	down	0.02	1
circRNA6297	0	80.24	0	132.90	147.53	560.41	-3.39	down	0.02	1
circRNA9580	0	0	0	398.70	36.88	0	-inf	down	0.02	1
circRNA4161	156.87	80.24	84.79	0	0	0	inf	up	0.02	1
circRNA3440	104.58	0	395.70	0	0	0	inf	up	0.02	1
circRNA9956	0	0	0	53.16	110.65	150.88	-inf	down	0.02	1
circRNA10945	0	0	0	0	73.77	452.64	-inf	down	0.02	1
circRNA2833	392.18	240.73	423.97	26.58	0	150.88	2.57	up	0.02	1
circRNA9787	0	0	0	79.74	73.77	129.33	-inf	down	0.02	1
circRNA4577	0	294.23	141.32	0	0	0	inf	up	0.02	1
circRNA4178	104.58	0	0	212.64	258.18	215.54	-2.71	down	0.02	1
circRNA4051	130.73	133.74	141.32	0	0	43.11	3.23	up	0.02	1

circRNA10087	0	0	0	239.22	0	129.33	-inf	down	0.03	1
circRNA8570	0	0	56.53	79.74	147.53	344.87	-3.34	down	0.03	1
circRNA1389	104.58	133.74	141.32	26.58	0	0	3.84	up	0.03	1
circRNA9813	0	0	0	79.74	147.53	64.66	-inf	down	0.03	1
circRNA10089	0	0	0	132.90	258.18	0	-inf	down	0.03	1
ciRNA464	0	0	0	79.74	0	366.42	-inf	down	0.03	1
circRNA3901	130.73	160.49	84.79	850.57	405.71	258.65	-2.01	down	0.03	1
circRNA4164	183.02	53.50	84.79	0	0	0	inf	up	0.03	1
circRNA3867	78.44	160.49	113.06	664.51	737.66	172.43	-2.16	down	0.03	1
circRNA4167	130.73	80.24	84.79	0	0	0	inf	up	0.03	1
circRNA9848	0	0	0	106.32	147.53	43.11	-inf	down	0.03	1
circRNA9231	0	0	0	106.32	147.53	43.11	-inf	down	0.03	1
circRNA3896	156.87	294.23	197.85	0	0	86.22	2.91	up	0.03	1
circRNA3554	78.44	80.24	113.06	0	0	0	inf	up	0.03	1
circRNA3951	78.44	106.99	84.79	0	0	0	inf	up	0.03	1
circRNA627	104.58	187.23	84.79	0	0	21.55	4.13	up	0.03	1
circRNA3916	78.44	213.98	226.12	0	0	64.66	3.00	up	0.03	1
circRNA971	130.73	53.50	113.06	0	0	0	inf	up	0.03	1
circRNA925	104.58	53.50	141.32	0	0	0	inf	up	0.03	1
circRNA2927	235.31	160.49	0	0	0	0	inf	up	0.03	1
circRNA968	26.15	213.98	84.79	0	0	0	inf	up	0.03	1
circRNA8095	0	0	84.79	132.90	147.53	366.42	-2.93	down	0.04	1
circRNA3534	52.29	53.50	197.85	0	0	0	inf	up	0.04	1
circRNA3187	156.87	53.50	84.79	0	0	0	inf	up	0.04	1
circRNA3123	209.17	133.74	56.53	0	0	43.11	3.21	up	0.04	1
circRNA6268	0	240.73	141.32	0	0	0	inf	up	0.04	1
circRNA2611	104.58	106.99	56.53	0	0	0	inf	up	0.04	1
circRNA3971	104.58	106.99	56.53	0	0	0	inf	up	0.04	1
circRNA2113	104.58	26.75	28.26	451.86	368.83	43.11	-2.44	down	0.04	1
circRNA1986	52.29	106.99	113.06	0	0	0	inf	up	0.04	1
circRNA9796	0	0	0	79.74	110.65	64.66	-inf	down	0.04	1
circRNA1505	156.87	133.74	84.79	26.58	0	0	3.82	up	0.04	1
circRNA9744	0	0	0	186.06	147.53	0	-inf	down	0.04	1
circRNA8245	0	0	56.53	558.18	184.41	0	-3.72	down	0.04	1
circRNA3331	52.29	187.23	56.53	0	0	0	inf	up	0.04	1
circRNA9455	0	0	0	186.06	147.53	0	-inf	down	0.04	1
circRNA10019	0	0	0	186.06	147.53	0	-inf	down	0.04	1
circRNA3995	78.44	53.50	141.32	0	0	0	inf	up	0.04	1
circRNA287	104.58	160.49	28.26	0	0	0	inf	up	0.04	1
circRNA3280	52.29	80.24	141.32	0	0	0	inf	up	0.04	1
circRNA3143	104.58	133.74	113.06	0	0	43.11	3.03	up	0.04	1

circRNA2885	26.15	160.49	113.06	0	0	0	inf	up	0.04	1
circRNA2601	26.15	26.75	254.38	186.06	258.18	1595.01	-2.73	down	0.04	1
circRNA9456	0	0	0	212.64	110.65	0	-inf	down	0.04	1
circRNA8626	0	0	0	106.32	73.77	64.66	-inf	down	0.04	1
circRNA998	104.58	26.75	56.53	186.06	221.30	323.31	-1.96	down	0.04	1
ciRNA87	209.17	160.49	0	0	0	0	inf	up	0.04	1
circRNA3835	52.29	0	0	106.32	184.41	150.88	-3.08	down	0.04	1
circRNA1138	78.44	0	0	505.02	110.65	64.66	-3.12	down	0.04	1
circRNA1898	104.58	0	28.26	637.93	368.83	0	-2.92	down	0.04	1
circRNA6381	0	160.49	197.85	0	0	0	inf	up	0.04	1
circRNA9753	0	0	0	79.74	0	280.20	-inf	down	0.04	1
circRNA2402	52.29	80.24	28.26	159.48	184.41	387.98	-2.19	down	0.04	1
circRNA833	104.58	53.50	113.06	0	0	0	inf	up	0.05	1
circRNA8905	0	0	0	239.22	73.77	0	-inf	down	0.05	1
circRNA9254	0	0	0	132.90	184.41	0	-inf	down	0.05	1
circRNA9639	0	0	0	132.90	110.65	21.55	-inf	down	0.05	1
circRNA9889	0	0	0	159.48	147.53	0	-inf	down	0.05	1
circRNA9830	0	0	0	159.48	147.53	0	-inf	down	0.05	1
circRNA9176	0	0	0	132.90	73.77	43.11	-inf	down	0.05	1
circRNA3652	78.44	53.50	113.06	0	0	0	inf	up	0.05	1
circRNA710	104.58	80.24	56.53	0	0	0	inf	up	0.05	1
circRNA2463	104.58	80.24	56.53	0	0	0	inf	up	0.05	1
circRNA3157	78.44	106.99	56.53	0	0	0	inf	up	0.05	1
circRNA314	26.15	0	0	53.16	73.77	237.10	-3.80	down	0.05	1
circRNA4115	52.29	106.99	84.79	0	0	0	inf	up	0.05	1
circRNA3661	52.29	106.99	84.79	0	0	0	inf	up	0.05	1
circRNA4212	104.58	240.73	0	0	0	0	inf	up	0.05	1

Table S5 complete DECs lists (Stm vs Sad)

Accession	Stm1_srpbm	Stm3_srpbm	Stm2_srpbm	Sad2_srpbm	Sad1_srpbm	Sad3_srpbm	log_2FC	regulation	pvalue	qvalue
circRNA4250	0	0	0	647.26	921.02	371.50	-inf	down	0.00	0.26
circRNA2170	0	0	0	575.35	566.78	348.28	-inf	down	0.00	0.28
circRNA17143	0	0	0	215.75	283.39	789.43	-inf	down	0.00	0.76
circRNA2275	0	0	0	191.78	236.16	464.37	-inf	down	0.00	1
circRNA3187	0	0	0	215.75	330.62	278.62	-inf	down	0.00	1
circRNA7112	0	0	0	215.75	401.47	185.75	-inf	down	0.00	1
circRNA627	0	0	21.55	455.48	283.39	278.62	-5.56	down	0.00	1
circRNA7846	1196.11	442.60	0	0	0	0	inf	up	0.00	1
circRNA3938	0	0	0	287.67	188.93	232.18	-inf	down	0.00	1
circRNA17145	0	0	0	215.75	212.54	232.18	-inf	down	0.00	1
circRNA3752	0	0	0	287.67	188.93	185.75	-inf	down	0.00	1

circRNA16946	0	0	0	239.73	330.62	139.31	-inf	down	0.00	1
circRNA4282	0	0	0	191.78	448.70	92.87	-inf	down	0.00	1
circRNA1271	0	0	0	215.75	283.39	92.87	-inf	down	0.00	1
circRNA2994	0	0	43.11	527.40	307.01	325.06	-4.75	down	0.00	1
circRNA8412	956.89	553.24	107.77	0	70.85	69.66	3.53	up	0.00	1
circRNA8775	265.80	627.01	0	0	0	0	inf	up	0.00	1
circRNA10086	611.35	258.18	0	0	0	0	inf	up	0.00	1
circRNA16664	0	0	0	191.78	165.31	162.53	-inf	down	0.00	1
circRNA3071	0	0	0	143.84	188.93	185.75	-inf	down	0.00	1
circRNA6413	0	0	0	335.62	188.93	69.66	-inf	down	0.00	1
circRNA3095	0	0	0	215.75	165.31	139.31	-inf	down	0.00	1
circRNA817	0	0	0	167.81	141.70	185.75	-inf	down	0.01	1
circRNA3349	0	184.41	668.18	0	0	0	inf	up	0.01	1
circRNA3735	0	0	0	143.84	94.46	301.84	-inf	down	0.01	1
circRNA2631	0	0	0	143.84	94.46	301.84	-inf	down	0.01	1
circRNA8382	0	0	0	215.75	165.31	116.09	-inf	down	0.01	1
circRNA3054	0	0	0	215.75	118.08	162.53	-inf	down	0.01	1
circRNA3584	0	0	0	215.75	141.70	116.09	-inf	down	0.01	1
circRNA9958	292.38	368.83	0	0	0	0	inf	up	0.01	1
ciRNA86	425.28	331.95	258.65	95.89	0	0	3.41	up	0.01	1
circRNA9772	239.22	405.71	0	0	0	0	inf	up	0.01	1
circRNA9540	531.60	147.53	0	0	0	0	inf	up	0.01	1
circRNA4922	0	0	0	239.73	118.08	116.09	-inf	down	0.01	1
circRNA4289	79.74	73.77	193.99	0	0	0	inf	up	0.01	1
circRNA3554	0	0	0	119.86	141.70	185.75	-inf	down	0.01	1
circRNA425	0	0	0	71.92	212.54	208.97	-inf	down	0.01	1
circRNA16596	0	0	0	215.75	70.85	185.75	-inf	down	0.01	1
circRNA4577	0	0	0	0	543.17	325.06	-inf	down	0.01	1
circRNA3183	0	0	0	239.73	70.85	162.53	-inf	down	0.01	1
circRNA17290	0	0	0	167.81	188.93	92.87	-inf	down	0.01	1
circRNA3013	0	0	43.11	143.84	377.85	441.15	-4.48	down	0.01	1
circRNA1891	0	0	64.66	359.59	236.16	673.34	-4.29	down	0.01	1
circRNA3144	0	0	0	335.62	118.08	69.66	-inf	down	0.01	1
circRNA10051	505.02	110.65	0	0	0	0	inf	up	0.01	1
circRNA3696	0	0	0	47.95	354.24	139.31	-inf	down	0.01	1
circRNA4001	0	0	0	143.84	283.39	69.66	-inf	down	0.01	1
circRNA2233	0	0	0	143.84	283.39	69.66	-inf	down	0.01	1
circRNA6062	0	0	0	119.86	165.31	139.31	-inf	down	0.01	1
circRNA3734	0	0	0	143.84	118.08	162.53	-inf	down	0.01	1
circRNA3794	0	0	0	119.86	188.93	116.09	-inf	down	0.01	1
circRNA3463	0	0	0	95.89	236.16	116.09	-inf	down	0.01	1

circRNA4085	0	221.30	366.42	0	0	0	inf	up	0.01	1
circRNA5152	0	0	21.55	383.56	236.16	92.87	-5.05	down	0.01	1
circRNA5751	106.32	110.65	86.22	0	0	0	inf	up	0.01	1
circRNA3922	0	0	0	167.81	259.77	46.44	-inf	down	0.01	1
circRNA3531	0	0	0	215.75	118.08	92.87	-inf	down	0.01	1
circRNA9156	292.38	184.41	0	0	0	0	inf	up	0.01	1
circRNA2113	451.86	368.83	43.11	0	0	69.66	3.63	up	0.01	1
circRNA2927	0	0	0	359.59	0	325.06	-inf	down	0.01	1
circRNA9769	372.12	110.65	0	0	0	0	inf	up	0.01	1
circRNA9457	212.64	258.18	0	0	0	0	inf	up	0.01	1
circRNA6445	0	0	0	95.89	236.16	92.87	-inf	down	0.01	1
circRNA8504	0	0	0	47.95	118.08	301.84	-inf	down	0.01	1
circRNA9987	398.70	442.60	0	47.95	0	0	4.13	up	0.01	1
circRNA22762	0	0	0	215.75	188.93	46.44	-inf	down	0.01	1
circRNA5467	797.41	442.60	107.77	23.97	141.70	23.22	2.83	up	0.01	1
circRNA8458	0	0	0	71.92	236.16	116.09	-inf	down	0.01	1
circRNA3246	584.77	553.24	107.77	0	0	116.09	3.42	up	0.01	1
circRNA6082	0	0	64.66	479.45	283.39	255.40	-3.98	down	0.01	1
circRNA8513	0	0	0	239.73	118.08	69.66	-inf	down	0.01	1
circRNA3987	0	0	0	167.81	118.08	92.87	-inf	down	0.01	1
circRNA360	0	0	0	167.81	118.08	92.87	-inf	down	0.01	1
circRNA10244	0	36.88	21.55	215.75	212.54	534.02	-4.04	down	0.01	1
circRNA19564	0	0	0	311.65	330.62	0	-inf	down	0.01	1
circRNA4214	0	0	0	95.89	188.93	116.09	-inf	down	0.01	1
circRNA16706	0	0	0	95.89	118.08	185.75	-inf	down	0.01	1
circRNA17392	0	0	0	143.84	47.23	255.40	-inf	down	0.01	1
circRNA2518	0	0	0	335.62	94.46	46.44	-inf	down	0.01	1
circRNA1898	637.93	368.83	0	0	23.62	46.44	3.85	up	0.01	1
circRNA2110	0	0	0	191.78	188.93	46.44	-inf	down	0.01	1
circRNA3753	0	0	0	143.84	165.31	69.66	-inf	down	0.02	1
circRNA3767	0	0	0	143.84	165.31	69.66	-inf	down	0.02	1
circRNA6295	0	0	0	167.81	47.23	208.97	-inf	down	0.02	1
circRNA4211	0	0	0	167.81	70.85	139.31	-inf	down	0.02	1
circRNA9697	239.22	184.41	0	0	0	0	inf	up	0.02	1
circRNA2967	239.22	184.41	0	0	0	0	inf	up	0.02	1
circRNA16418	0	0	0	71.92	188.93	139.31	-inf	down	0.02	1
circRNA24136	0	0	0	191.78	141.70	69.66	-inf	down	0.02	1
circRNA3396	0	0	0	95.89	212.54	92.87	-inf	down	0.02	1
circRNA5727	0	0	0	215.75	94.46	92.87	-inf	down	0.02	1
circRNA3440	0	0	0	0	236.16	371.50	-inf	down	0.02	1
circRNA3760	0	0	0	71.92	212.54	116.09	-inf	down	0.02	1

circRNA9946	186.06	221.30	0	0	0	0	inf	up	0.02	1
circRNA10080	132.90	295.06	0	0	0	0	inf	up	0.02	1
circRNA440	0	0	0	71.92	94.46	208.97	-inf	down	0.02	1
circRNA1765	0	0	0	143.84	118.08	92.87	-inf	down	0.02	1
circRNA4010	0	0	0	119.86	141.70	92.87	-inf	down	0.02	1
circRNA1899	239.22	147.53	0	0	0	0	inf	up	0.02	1
circRNA16926	0	0	0	95.89	165.31	92.87	-inf	down	0.02	1
circRNA17150	0	0	0	167.81	94.46	92.87	-inf	down	0.02	1
circRNA3844	0	0	0	47.95	141.70	208.97	-inf	down	0.02	1
circRNA1061	265.80	110.65	0	0	0	0	inf	up	0.02	1
circRNA3262	345.54	442.60	581.96	0	0	185.75	2.88	up	0.02	1
circRNA6271	0	0	0	143.84	141.70	69.66	-inf	down	0.02	1
circRNA10089	132.90	258.18	0	0	0	0	inf	up	0.02	1
circRNA3635	0	0	0	167.81	118.08	69.66	-inf	down	0.02	1
circRNA2885	0	0	0	119.86	165.31	69.66	-inf	down	0.02	1
circRNA3638	0	0	64.66	191.78	307.01	394.71	-3.79	down	0.02	1
circRNA3145	0	0	0	95.89	141.70	92.87	-inf	down	0.02	1
circRNA2575	0	0	0	95.89	94.46	139.31	-inf	down	0.02	1
circRNA8413	0	0	0	71.92	188.93	92.87	-inf	down	0.02	1
circRNA16864	0	0	0	407.54	0	139.31	-inf	down	0.02	1
circRNA2503	0	0	0	143.84	70.85	116.09	-inf	down	0.02	1
circRNA8343	0	0	0	311.65	212.54	0	-inf	down	0.02	1
circRNA2535	0	0	0	119.86	283.39	23.22	-inf	down	0.02	1
circRNA3162	0	0	0	95.89	165.31	69.66	-inf	down	0.02	1
circRNA3692	0	0	107.77	167.81	307.01	859.08	-3.63	down	0.03	1
circRNA4018	0	0	0	167.81	94.46	69.66	-inf	down	0.03	1
circRNA6912	0	0	0	47.95	118.08	185.75	-inf	down	0.03	1
circRNA3439	0	0	0	71.92	212.54	69.66	-inf	down	0.03	1
circRNA6555	0	0	0	191.78	165.31	23.22	-inf	down	0.03	1
circRNA9744	186.06	147.53	0	0	0	0	inf	up	0.03	1
circRNA9455	186.06	147.53	0	0	0	0	inf	up	0.03	1
circRNA8118	0	0	0	47.95	283.39	69.66	-inf	down	0.03	1
circRNA10019	186.06	147.53	0	0	0	0	inf	up	0.03	1
circRNA9747	903.73	590.13	129.33	47.95	236.16	46.44	2.30	up	0.03	1
circRNA17054	0	0	0	215.75	0	278.62	-inf	down	0.03	1
circRNA8273	0	0	0	143.84	141.70	46.44	-inf	down	0.03	1
circRNA7250	0	0	0	311.65	165.31	0	-inf	down	0.03	1
circRNA7506	0	36.88	0	191.78	188.93	232.18	-4.05	down	0.03	1
circRNA9456	212.64	110.65	0	0	0	0	inf	up	0.03	1
circRNA3889	0	0	0	119.86	47.23	162.53	-inf	down	0.03	1
circRNA2368	0	0	0	167.81	118.08	46.44	-inf	down	0.03	1

circRNA17507	0	0	0	95.89	94.46	116.09	-inf	down	0.03	1
circRNA6075	0	0	0	167.81	47.23	116.09	-inf	down	0.03	1
circRNA4153	0	0	0	191.78	70.85	69.66	-inf	down	0.03	1
circRNA4104	0	0	0	119.86	94.46	92.87	-inf	down	0.03	1
circRNA4167	0	0	0	335.62	141.70	0	-inf	down	0.03	1
circRNA6415	0	0	0	0	354.24	139.31	-inf	down	0.03	1
circRNA3361	1116.37	737.66	172.43	263.70	165.31	116.09	1.89	up	0.03	1
circRNA3357	0	258.18	344.87	0	0	46.44	3.70	up	0.03	1
circRNA6254	0	0	107.77	407.54	259.77	371.50	-3.27	down	0.03	1
circRNA6416	0	0	0	47.95	47.23	278.62	-inf	down	0.03	1
circRNA17296	0	0	0	119.86	70.85	116.09	-inf	down	0.03	1
circRNA5609	0	0	43.11	191.78	283.39	139.31	-3.83	down	0.03	1
circRNA8434	0	0	0	191.78	94.46	46.44	-inf	down	0.03	1
circRNA4181	0	0	0	191.78	47.23	92.87	-inf	down	0.03	1
circRNA7610	0	0	172.43	431.51	661.24	673.34	-3.36	down	0.03	1
circRNA24668	0	0	0	71.92	141.70	92.87	-inf	down	0.03	1
circRNA24495	0	0	0	71.92	141.70	92.87	-inf	down	0.03	1
circRNA8467	0	0	0	71.92	94.46	139.31	-inf	down	0.03	1
circRNA6257	0	0	43.11	143.84	212.54	208.97	-3.71	down	0.03	1
circRNA8067	53.16	110.65	64.66	0	0	0	inf	up	0.03	1
circRNA3036	0	0	43.11	311.65	236.16	92.87	-3.89	down	0.03	1
circRNA3331	0	0	0	263.70	188.93	0	-inf	down	0.03	1
circRNA5274	0	0	0	0	259.77	185.75	-inf	down	0.03	1
circRNA6788	0	0	64.66	95.89	212.54	603.68	-3.82	down	0.03	1
circRNA6141	0	0	0	71.92	212.54	46.44	-inf	down	0.03	1
circRNA3539	451.86	995.84	2370.96	5274.00	8596.18	5781.39	-2.36	down	0.04	1
circRNA4267	0	0	0	71.92	165.31	69.66	-inf	down	0.04	1
ciRNA117	0	0	0	143.84	118.08	46.44	-inf	down	0.04	1
circRNA2559	53.16	73.77	129.33	503.43	614.01	464.37	-2.63	down	0.04	1
ciRNA463	186.06	110.65	0	0	0	0	inf	up	0.04	1
circRNA9966	186.06	110.65	0	0	0	0	inf	up	0.04	1
circRNA8091	0	0	0	215.75	212.54	0	-inf	down	0.04	1
circRNA2394	0	36.88	0	239.73	165.31	139.31	-3.88	down	0.04	1
circRNA19989	0	0	0	23.97	165.31	139.31	-inf	down	0.04	1
circRNA3937	0	0	0	95.89	165.31	46.44	-inf	down	0.04	1
circRNA5108	0	0	0	95.89	94.46	92.87	-inf	down	0.04	1
circRNA17571	0	0	0	95.89	94.46	92.87	-inf	down	0.04	1
circRNA17562	0	0	0	95.89	94.46	92.87	-inf	down	0.04	1
circRNA10381	0	331.95	0	1845.90	1487.80	1044.83	-3.72	down	0.04	1
circRNA5960	53.16	0	0	215.75	259.77	255.40	-3.78	down	0.04	1
circRNA2581	0	0	21.55	119.86	141.70	139.31	-4.22	down	0.04	1

circRNA8540	0	0	0	71.92	94.46	116.09	-inf	down	0.04	1
circRNA6091	0	0	0	95.89	118.08	69.66	-inf	down	0.04	1
circRNA17074	0	0	0	95.89	94.46	69.66	-inf	down	0.04	1
circRNA3793	0	0	0	95.89	94.46	69.66	-inf	down	0.04	1
circRNA10944	0	221.30	107.77	0	0	0	inf	up	0.04	1
circRNA3032	0	0	0	119.86	94.46	69.66	-inf	down	0.04	1
circRNA4035	0	0	0	119.86	94.46	69.66	-inf	down	0.04	1
circRNA10010	212.64	73.77	0	0	0	0	inf	up	0.04	1
circRNA17621	0	0	0	47.95	47.23	232.18	-inf	down	0.04	1
circRNA3902	292.38	147.53	0	0	0	46.44	3.24	up	0.04	1
circRNA9973	132.90	147.53	0	0	0	0	inf	up	0.04	1
circRNA3381	0	0	0	71.92	188.93	46.44	-inf	down	0.04	1
circRNA2580	0	0	0	71.92	118.08	69.66	-inf	down	0.04	1
circRNA402	0	0	0	191.78	47.23	69.66	-inf	down	0.04	1
circRNA7338	79.74	0	215.54	0	0	0	inf	up	0.04	1
circRNA3540	0	0	129.33	503.43	448.70	208.97	-3.17	down	0.04	1
circRNA1270	292.38	663.89	1810.55	3667.82	5620.58	4388.29	-2.31	down	0.04	1
circRNA11292	0	0	43.11	143.84	212.54	162.53	-3.59	down	0.04	1
circRNA1062	265.80	0	129.33	0	23.62	0	4.06	up	0.04	1
circRNA8819	186.06	221.30	0	0	23.62	0	4.11	up	0.04	1
circRNA8297	0	0	0	215.75	188.93	0	-inf	down	0.05	1
circRNA22990	0	0	0	143.84	70.85	69.66	-inf	down	0.05	1
circRNA8111	0	0	0	143.84	70.85	69.66	-inf	down	0.05	1
circRNA9282	26.58	0	43.11	119.86	236.16	278.62	-3.19	down	0.05	1
circRNA863	0	0	0	143.84	70.85	69.66	-inf	down	0.05	1
circRNA6357	0	0	0	239.73	165.31	0	-inf	down	0.05	1
circRNA3516	0	0	43.11	0	354.24	510.81	-4.33	down	0.05	1
circRNA5979	0	0	0	47.95	118.08	116.09	-inf	down	0.05	1
circRNA17044	0	0	0	47.95	118.08	116.09	-inf	down	0.05	1
circRNA3173	0	0	0	191.78	188.93	0	-inf	down	0.05	1
circRNA3004	0	0	43.11	215.75	118.08	185.75	-3.59	down	0.05	1
circRNA6194	0	0	0	167.81	0	208.97	-inf	down	0.05	1
circRNA3632	0	0	0	215.75	165.31	0	-inf	down	0.05	1
circRNA16306	0	0	0	215.75	165.31	0	-inf	down	0.05	1
circRNA8267	0	0	0	167.81	212.54	0	-inf	down	0.05	1
circRNA6296	0	0	0	167.81	212.54	0	-inf	down	0.05	1
circRNA17272	0	0	0	47.95	118.08	92.87	-inf	down	0.05	1
circRNA16503	0	0	0	47.95	94.46	116.09	-inf	down	0.05	1
circRNA15378	0	0	0	47.95	94.46	116.09	-inf	down	0.05	1
circRNA29846	0	0	0	119.86	47.23	116.09	-inf	down	0.05	1
circRNA24949	0	0	0	119.86	47.23	92.87	-inf	down	0.05	1

circRNA47487	0	0	0	95.89	47.23	116.09	-inf	down	0.05	1
circRNA6356	0	0	0	95.89	47.23	116.09	-inf	down	0.05	1
circRNA5927	0	0	0	95.89	47.23	116.09	-inf	down	0.05	1
circRNA7845	239.22	147.53	0	0	0	23.22	4.06	up	0.05	1
circRNA25954	0	0	0	47.95	141.70	92.87	-inf	down	0.05	1
circRNA2302	0	73.77	172.43	599.32	212.54	998.39	-2.88	down	0.05	1
circRNA16905	0	0	0	0	141.70	255.40	-inf	down	0.05	1
circRNA51906	0	0	0	0	259.77	139.31	-inf	down	0.05	1
circRNA687	159.48	73.77	64.66	839.04	826.56	348.28	-2.76	down	0.05	1
circRNA8514	0	0	0	95.89	47.23	139.31	-inf	down	0.05	1
circRNA16564	0	0	0	95.89	47.23	139.31	-inf	down	0.05	1
circRNA4230	0	0	43.11	335.62	212.54	46.44	-3.79	down	0.05	1
circRNA8963	79.74	110.65	21.55	0	0	0	inf	up	0.05	1
circRNA8237	0	0	0	143.84	47.23	92.87	-inf	down	0.05	1
circRNA10100	186.06	73.77	0	0	0	0	inf	up	0.05	1
circRNA2497	26.58	110.65	172.43	0	23.62	23.22	2.73	up	0.05	1

Table S6 complete DECs lists (Som vs Sad)

Accession	Som1_srpbm	Som2_srpbm	Som3_srpbm	Sad2_srpbm	Sad1_srpbm	Sad3_srpbm	log_2FC	regulation	pvalue	qvalue
circRNA10381	0	0	0	1845.90	1487.80	1044.83	-inf	down	0.00	0.00
circRNA10101	0	0	0	743.15	590.40	348.28	-inf	down	0.00	0.02
circRNA8910	0	0	0	623.29	283.39	441.15	-inf	down	0.00	0.05
circRNA10867	0	0	0	503.43	472.32	255.40	-inf	down	0.00	0.06
circRNA17143	0	0	0	215.75	283.39	789.43	-inf	down	0.00	0.10
circRNA10244	0	0	0	215.75	212.54	534.02	-inf	down	0.00	0.22
circRNA9976	0	0	0	0	1653.11	2136.10	-inf	down	0.00	0.28
circRNA1817	418.33	213.98	310.91	0	47.23	23.22	3.74	up	0.00	0.32
circRNA17145	0	0	0	215.75	212.54	232.18	-inf	down	0.00	0.37
circRNA9608	0	0	0	143.84	188.93	487.59	-inf	down	0.00	0.37
circRNA16946	0	0	0	239.73	330.62	139.31	-inf	down	0.00	0.37
circRNA13739	0	0	0	167.81	212.54	232.18	-inf	down	0.00	0.37
circRNA9282	0	0	0	119.86	236.16	278.62	-inf	down	0.00	0.37
circRNA13493	0	0	0	239.73	118.08	371.50	-inf	down	0.00	0.37
circRNA9925	0	0	0	239.73	188.93	162.53	-inf	down	0.00	0.41
circRNA10948	0	0	0	287.67	118.08	232.18	-inf	down	0.00	0.43
circRNA11099	0	0	0	191.78	165.31	185.75	-inf	down	0.00	0.45
circRNA10039	0	0	0	335.62	165.31	116.09	-inf	down	0.00	0.45
circRNA16664	0	0	0	191.78	165.31	162.53	-inf	down	0.00	0.52
circRNA13276	0	0	0	71.92	118.08	719.77	-inf	down	0.00	0.52
circRNA11292	0	0	0	143.84	212.54	162.53	-inf	down	0.00	0.52
circRNA2330	52.29	0	84.79	647.26	377.85	766.21	-3.71	down	0.00	0.60

circRNA11016	0	0	0	239.73	165.31	116.09	-inf	down	0.00	0.60
circRNA12406	0	0	0	143.84	165.31	185.75	-inf	down	0.00	0.60
circRNA9848	0	0	0	191.78	236.16	92.87	-inf	down	0.00	0.64
circRNA9956	0	0	0	215.75	141.70	139.31	-inf	down	0.00	0.66
circRNA11165	0	0	0	167.81	236.16	92.87	-inf	down	0.00	0.76
circRNA13942	0	0	0	47.95	259.77	255.40	-inf	down	0.00	0.78
circRNA194	26.15	0	0	119.86	259.77	325.06	-4.75	down	0.00	0.80
circRNA10314	0	0	0	95.89	94.46	371.50	-inf	down	0.00	0.80
circRNA9884	0	0	0	215.75	118.08	139.31	-inf	down	0.00	0.80
circRNA13502	0	0	0	191.78	118.08	139.31	-inf	down	0.00	0.81
circRNA16596	0	0	0	215.75	70.85	185.75	-inf	down	0.00	0.81
circRNA10975	0	0	0	143.84	94.46	232.18	-inf	down	0.00	0.81
circRNA11102	0	0	0	119.86	165.31	162.53	-inf	down	0.00	0.81
circRNA13510	0	0	0	119.86	70.85	325.06	-inf	down	0.00	0.81
circRNA13865	0	0	0	119.86	141.70	162.53	-inf	down	0.00	0.81
circRNA3350	209.17	374.47	0	0	0	0	inf	up	0.00	0.81
circRNA9894	0	0	0	167.81	236.16	69.66	-inf	down	0.00	0.81
circRNA13304	0	0	0	47.95	236.16	208.97	-inf	down	0.01	0.81
circRNA3351	156.87	133.74	56.53	0	0	0	inf	up	0.01	0.81
circRNA17290	0	0	0	167.81	188.93	92.87	-inf	down	0.01	0.81
circRNA6841	0	0	56.53	239.73	330.62	417.93	-4.13	down	0.01	0.81
circRNA9909	0	0	0	335.62	425.09	0	-inf	down	0.01	0.81
circRNA14108	0	0	0	167.81	70.85	208.97	-inf	down	0.01	0.81
circRNA3445	52.29	0	0	503.43	401.47	162.53	-4.35	down	0.01	0.81
circRNA8626	0	0	0	359.59	47.23	116.09	-inf	down	0.01	0.81
circRNA8875	0	0	0	239.73	47.23	208.97	-inf	down	0.01	0.81
circRNA3146	156.87	401.22	0	0	0	0	inf	up	0.01	0.82
circRNA13387	0	0	0	95.89	118.08	208.97	-inf	down	0.01	0.82
circRNA13842	0	0	0	47.95	212.54	208.97	-inf	down	0.01	0.85
circRNA10097	0	0	0	0	401.47	325.06	-inf	down	0.01	0.85
circRNA565	104.58	80.24	56.53	335.62	708.48	998.39	-3.08	down	0.01	0.85
circRNA9860	0	0	0	119.86	118.08	162.53	-inf	down	0.01	0.85
circRNA22762	0	0	0	215.75	188.93	46.44	-inf	down	0.01	0.85
circRNA13583	0	0	0	0	283.39	394.71	-inf	down	0.01	0.85
circRNA19564	0	0	0	311.65	330.62	0	-inf	down	0.01	0.85
circRNA13932	0	0	0	167.81	141.70	92.87	-inf	down	0.01	0.85
circRNA4099	52.29	0	0	263.70	354.24	255.40	-4.06	down	0.01	0.85
circRNA16706	0	0	0	95.89	118.08	185.75	-inf	down	0.01	0.85
circRNA4279	104.58	0	56.53	455.48	708.48	348.28	-3.23	down	0.01	0.85
circRNA3560	130.73	106.99	169.59	0	0	46.44	3.13	up	0.01	0.85
circRNA17392	0	0	0	143.84	47.23	255.40	-inf	down	0.01	0.85

circRNA10017	0	0	0	71.92	141.70	208.97	-inf	down	0.01	0.85
circRNA13825	0	0	0	119.86	141.70	116.09	-inf	down	0.01	0.85
circRNA9014	0	0	0	71.92	188.93	139.31	-inf	down	0.01	0.85
circRNA16418	0	0	0	71.92	188.93	139.31	-inf	down	0.01	0.85
ciRNA79	156.87	80.24	169.59	0	0	46.44	3.13	up	0.01	0.85
circRNA7060	0	0	28.26	335.62	118.08	116.09	-4.33	down	0.01	0.85
circRNA11053	0	0	0	143.84	70.85	185.75	-inf	down	0.01	0.85
circRNA24136	0	0	0	191.78	141.70	69.66	-inf	down	0.01	0.85
circRNA2631	26.15	0	0	143.84	94.46	301.84	-4.37	down	0.01	0.85
circRNA3692	52.29	0	56.53	167.81	307.01	859.08	-3.62	down	0.01	0.85
circRNA8551	0	0	84.79	455.48	236.16	441.15	-3.74	down	0.01	0.85
circRNA642	26.15	0	0	143.84	212.54	139.31	-4.24	down	0.01	0.85
circRNA10601	0	0	0	119.86	165.31	92.87	-inf	down	0.01	0.85
circRNA9796	0	0	0	143.84	94.46	139.31	-inf	down	0.01	0.85
circRNA4039	78.44	80.24	310.91	0	47.23	0	3.31	up	0.01	0.85
circRNA3903	78.44	0	0	455.48	259.77	371.50	-3.79	down	0.01	0.85
circRNA3262	444.48	615.20	282.64	0	0	185.75	2.85	up	0.01	0.85
circRNA11045	0	0	0	119.86	70.85	208.97	-inf	down	0.01	0.85
circRNA3887	183.02	53.50	84.79	0	0	0	inf	up	0.01	0.85
circRNA3297	52.29	0	84.79	8989.77	70.85	0	-6.05	down	0.01	0.85
circRNA9838	0	0	0	431.51	0	162.53	-inf	down	0.01	0.85
circRNA13961	0	0	0	0	212.54	394.71	-inf	down	0.01	0.85
circRNA925	104.58	53.50	141.32	0	0	0	inf	up	0.01	0.86
circRNA9254	0	0	0	167.81	0	417.93	-inf	down	0.01	0.87
circRNA5517	0	53.50	0	119.86	236.16	742.99	-4.36	down	0.01	0.87
circRNA7506	0	0	28.26	191.78	188.93	232.18	-4.44	down	0.01	0.87
circRNA13841	0	0	0	47.95	165.31	185.75	-inf	down	0.01	0.87
circRNA11017	0	0	0	47.95	141.70	232.18	-inf	down	0.01	0.87
circRNA9488	0	0	0	47.95	141.70	232.18	-inf	down	0.01	0.87
circRNA13970	0	0	0	191.78	165.31	46.44	-inf	down	0.01	0.89
circRNA3393	104.58	80.24	84.79	0	0	0	inf	up	0.01	0.89
circRNA13738	0	0	0	0	354.24	208.97	-inf	down	0.01	0.89
circRNA4056	339.89	240.73	113.06	0	0	92.87	2.90	up	0.01	0.89
circRNA10474	0	0	0	71.92	212.54	92.87	-inf	down	0.01	0.89
circRNA16864	0	0	0	407.54	0	139.31	-inf	down	0.01	0.89
circRNA8583	0	0	56.53	239.73	236.16	301.84	-3.78	down	0.01	0.89
circRNA13428	0	0	0	0	141.70	441.15	-inf	down	0.01	0.89
circRNA16926	0	0	0	95.89	165.31	92.87	-inf	down	0.01	0.90
circRNA3534	52.29	53.50	197.85	0	0	0	inf	up	0.01	0.91
circRNA351	287.60	80.24	56.53	0	0	23.22	4.19	up	0.01	0.91
circRNA10925	0	0	0	263.70	0	255.40	-inf	down	0.01	0.91

circRNA3677	78.44	0	113.06	527.40	519.55	464.37	-2.98	down	0.01	0.92
circRNA17150	0	0	0	167.81	94.46	92.87	-inf	down	0.01	0.92
circRNA12076	0	0	0	119.86	165.31	69.66	-inf	down	0.01	0.92
circRNA3971	104.58	106.99	56.53	0	0	0	inf	up	0.01	0.92
circRNA9517	0	0	0	143.84	70.85	139.31	-inf	down	0.01	0.92
circRNA10132	0	0	0	263.70	70.85	69.66	-inf	down	0.01	0.92
circRNA1986	52.29	106.99	113.06	0	0	0	inf	up	0.02	0.94
circRNA3349	130.73	0	282.64	0	0	0	inf	up	0.02	0.94
circRNA10066	0	0	0	167.81	70.85	116.09	-inf	down	0.02	0.94
circRNA13849	0	0	0	71.92	94.46	185.75	-inf	down	0.02	0.95
circRNA6268	0	240.73	141.32	0	0	0	inf	up	0.02	0.95
circRNA3280	52.29	80.24	141.32	0	0	0	inf	up	0.02	0.95
circRNA11023	0	0	0	95.89	70.85	185.75	-inf	down	0.02	0.95
circRNA13821	0	0	0	95.89	118.08	116.09	-inf	down	0.02	0.95
circRNA17054	0	0	0	215.75	0	278.62	-inf	down	0.02	0.95
circRNA9948	0	0	0	119.86	118.08	92.87	-inf	down	0.02	0.95
ciRNA546	0	0	0	47.95	94.46	232.18	-inf	down	0.02	0.95
ciRNA87	209.17	160.49	0	0	0	0	inf	up	0.02	0.95
circRNA9920	0	0	0	119.86	94.46	116.09	-inf	down	0.02	0.95
circRNA11626	0	0	0	47.95	141.70	162.53	-inf	down	0.02	0.95
circRNA6254	0	53.50	56.53	407.54	259.77	371.50	-3.24	down	0.02	0.95
ciRNA405	0	0	0	215.75	0	255.40	-inf	down	0.02	0.95
circRNA12357	0	0	0	239.73	0	232.18	-inf	down	0.02	0.95
circRNA8783	0	0	0	95.89	94.46	139.31	-inf	down	0.02	0.95
circRNA10908	0	0	0	95.89	94.46	139.31	-inf	down	0.02	0.95
circRNA14195	0	0	0	47.95	188.93	116.09	-inf	down	0.02	0.95
circRNA3838	78.44	80.24	0	431.51	637.63	278.62	-3.09	down	0.02	0.95
circRNA11200	0	0	0	143.84	47.23	162.53	-inf	down	0.02	0.95
circRNA11151	0	0	0	95.89	94.46	116.09	-inf	down	0.02	0.95
circRNA9231	0	0	0	95.89	94.46	116.09	-inf	down	0.02	0.95
circRNA17507	0	0	0	95.89	94.46	116.09	-inf	down	0.02	0.95
circRNA6381	0	160.49	197.85	0	0	0	inf	up	0.02	0.95
circRNA1865	52.29	160.49	113.06	791.10	448.70	789.43	-2.64	down	0.02	0.95
circRNA6788	0	0	56.53	95.89	212.54	603.68	-4.01	down	0.02	0.95
circRNA4112	261.46	106.99	0	0	0	0	inf	up	0.02	0.95
circRNA9190	0	0	0	119.86	70.85	139.31	-inf	down	0.02	0.95
circRNA4303	52.29	0	56.53	239.73	307.01	325.06	-3.00	down	0.02	0.95
circRNA9889	0	0	0	335.62	0	139.31	-inf	down	0.02	0.95
circRNA1773	104.58	53.50	197.85	23.97	0	0	3.89	up	0.02	0.95
circRNA682	26.15	53.50	56.53	143.84	377.85	673.34	-3.13	down	0.02	0.95
circRNA10917	0	0	0	71.92	165.31	92.87	-inf	down	0.02	0.95

circRNA2463	104.58	80.24	56.53	0	0	0	inf	up	0.02	0.95
circRNA10136	0	0	0	191.78	283.39	0	-inf	down	0.02	0.95
circRNA608	52.29	0	0	287.67	141.70	301.84	-3.81	down	0.02	0.95
circRNA11386	0	0	0	23.97	141.70	208.97	-inf	down	0.02	0.95
circRNA17296	0	0	0	119.86	70.85	116.09	-inf	down	0.02	0.95
circRNA4270	52.29	80.24	113.06	0	0	0	inf	up	0.02	0.95
circRNA3157	78.44	106.99	56.53	0	0	0	inf	up	0.02	0.95
circRNA3040	130.73	106.99	84.79	0	47.23	0	2.77	up	0.02	0.95
circRNA14197	0	0	0	95.89	70.85	139.31	-inf	down	0.02	0.95
circRNA3661	52.29	106.99	84.79	0	0	0	inf	up	0.02	0.95
circRNA3055	52.29	0	0	287.67	212.54	185.75	-3.71	down	0.02	0.95
circRNA704	52.29	53.50	84.79	335.62	543.17	301.84	-2.63	down	0.02	0.96
circRNA12683	0	0	0	191.78	0	255.40	-inf	down	0.02	0.96
circRNA3113	287.60	0	0	1462.34	991.87	812.65	-3.51	down	0.02	0.98
circRNA10403	0	0	0	143.84	141.70	46.44	-inf	down	0.02	1
circRNA420	78.44	80.24	56.53	503.43	401.47	325.06	-2.51	down	0.02	1
circRNA2085	52.29	187.23	28.26	0	0	0	inf	up	0.02	1
circRNA4291	52.29	0	0	311.65	236.16	139.31	-3.72	down	0.02	1
circRNA11412	0	0	0	95.89	94.46	92.87	-inf	down	0.03	1
circRNA17571	0	0	0	95.89	94.46	92.87	-inf	down	0.03	1
circRNA17562	0	0	0	95.89	94.46	92.87	-inf	down	0.03	1
circRNA3407	130.73	133.74	56.53	0	47.23	0	2.76	up	0.03	1
circRNA10979	0	0	0	95.89	47.23	185.75	-inf	down	0.03	1
circRNA760	26.15	0	28.26	311.65	118.08	208.97	-3.55	down	0.03	1
circRNA9764	0	0	0	71.92	141.70	92.87	-inf	down	0.03	1
circRNA9753	0	0	0	71.92	141.70	92.87	-inf	down	0.03	1
circRNA24668	0	0	0	71.92	141.70	92.87	-inf	down	0.03	1
circRNA24495	0	0	0	71.92	141.70	92.87	-inf	down	0.03	1
circRNA1842	78.44	80.24	56.53	0	0	0	inf	up	0.03	1
circRNA13800	0	0	0	71.92	94.46	139.31	-inf	down	0.03	1
circRNA2603	78.44	80.24	56.53	0	0	0	inf	up	0.03	1
ciRNA86	418.33	588.45	0	95.89	0	0	3.39	up	0.03	1
circRNA13547	0	0	0	167.81	259.77	0	-inf	down	0.03	1
circRNA7782	0	0	28.26	167.81	141.70	92.87	-3.83	down	0.03	1
circRNA13919	0	0	0	95.89	118.08	69.66	-inf	down	0.03	1
circRNA10994	0	0	0	167.81	94.46	46.44	-inf	down	0.03	1
circRNA11167	0	0	0	95.89	70.85	116.09	-inf	down	0.03	1
circRNA9776	0	0	0	95.89	70.85	116.09	-inf	down	0.03	1
circRNA1908	26.15	0	56.53	191.78	236.16	278.62	-3.10	down	0.03	1
circRNA6493	0	0	56.53	95.89	330.62	278.62	-3.64	down	0.03	1
circRNA3454	261.46	160.49	197.85	47.95	70.85	69.66	1.72	up	0.03	1

circRNA4609	0	80.24	0	263.70	401.47	185.75	-3.41	down	0.03	1
circRNA11170	0	0	0	71.92	141.70	69.66	-inf	down	0.03	1
circRNA13292	0	0	0	47.95	141.70	116.09	-inf	down	0.03	1
circRNA11171	0	0	0	71.92	70.85	139.31	-inf	down	0.03	1
circRNA10164	0	0	0	71.92	70.85	139.31	-inf	down	0.03	1
circRNA17044	0	0	0	47.95	118.08	116.09	-inf	down	0.03	1
circRNA998	104.58	26.75	56.53	431.51	401.47	278.62	-2.56	down	0.03	1
circRNA19989	0	0	0	23.97	165.31	139.31	-inf	down	0.03	1
circRNA25954	0	0	0	47.95	141.70	92.87	-inf	down	0.03	1
circRNA22990	0	0	0	143.84	70.85	69.66	-inf	down	0.03	1
circRNA10541	0	0	0	47.95	94.46	139.31	-inf	down	0.03	1
ciRNA534	0	0	0	47.95	165.31	92.87	-inf	down	0.03	1
circRNA22	732.08	80.24	310.91	0	94.46	139.31	2.26	up	0.03	1
ciRNA434	0	0	0	119.86	118.08	46.44	-inf	down	0.03	1
circRNA29846	0	0	0	119.86	47.23	116.09	-inf	down	0.03	1
circRNA4035	313.75	133.74	395.70	119.86	94.46	69.66	1.57	up	0.03	1
circRNA4485	0	26.75	0	167.81	70.85	278.62	-4.27	down	0.03	1
circRNA3295	52.29	0	0	239.73	118.08	278.62	-3.61	down	0.03	1
circRNA2896	26.15	106.99	310.91	0	47.23	23.22	2.66	up	0.03	1
circRNA16564	0	0	0	95.89	47.23	139.31	-inf	down	0.03	1
circRNA731	26.15	0	0	215.75	23.62	208.97	-4.10	down	0.04	1
circRNA9747	0	0	0	47.95	236.16	46.44	-inf	down	0.04	1
circRNA6579	0	0	28.26	239.73	307.01	0	-4.27	down	0.04	1
circRNA3979	52.29	0	0	191.78	259.77	69.66	-3.32	down	0.04	1
circRNA16866	0	0	0	47.95	165.31	69.66	-inf	down	0.04	1
circRNA17621	0	0	0	47.95	47.23	232.18	-inf	down	0.04	1
circRNA14203	0	0	0	95.89	94.46	69.66	-inf	down	0.04	1
circRNA17074	0	0	0	95.89	94.46	69.66	-inf	down	0.04	1
circRNA14048	0	0	0	95.89	94.46	69.66	-inf	down	0.04	1
circRNA686	78.44	160.49	56.53	287.67	448.70	975.17	-2.53	down	0.04	1
circRNA9912	0	0	0	119.86	283.39	0	-inf	down	0.04	1
circRNA13451	0	0	0	47.95	188.93	69.66	-inf	down	0.04	1
circRNA11109	0	0	0	47.95	70.85	162.53	-inf	down	0.04	1
circRNA1884	104.58	106.99	0	479.45	283.39	650.12	-2.74	down	0.04	1
circRNA577	78.44	106.99	28.26	0	0	0	inf	up	0.04	1
circRNA3121	52.29	0	0	167.81	283.39	69.66	-3.32	down	0.04	1
circRNA11055	0	0	0	0	70.85	371.50	-inf	down	0.04	1
circRNA2967	52.29	53.50	84.79	0	0	0	inf	up	0.04	1
circRNA6009	0	80.24	226.12	0	0	0	inf	up	0.04	1
circRNA4090	52.29	0	0	287.67	188.93	139.31	-3.56	down	0.04	1
circRNA3806	130.73	80.24	84.79	0	0	46.44	2.67	up	0.04	1

circRNA3912	52.29	133.74	0	311.65	401.47	580.46	-2.80	down	0.04	1
circRNA3448	261.46	240.73	113.06	47.95	118.08	0	1.89	up	0.04	1
circRNA6388	0	106.99	169.59	455.48	472.32	835.86	-2.67	down	0.04	1
circRNA4280	130.73	160.49	84.79	71.92	0	0	2.39	up	0.04	1
circRNA13344	0	0	0	167.81	47.23	69.66	-inf	down	0.04	1
circRNA4225	156.87	454.71	226.12	119.86	118.08	46.44	1.56	up	0.04	1
circRNA16624	0	0	0	191.78	0	162.53	-inf	down	0.04	1
circRNA16306	0	0	0	215.75	165.31	0	-inf	down	0.04	1
circRNA3241	52.29	0	0	215.75	141.70	232.18	-3.50	down	0.04	1
circRNA10974	0	0	0	0	165.31	232.18	-inf	down	0.04	1
circRNA9690	0	0	0	119.86	70.85	69.66	-inf	down	0.04	1
circRNA17272	0	0	0	47.95	118.08	92.87	-inf	down	0.04	1
circRNA369	26.15	0	0	215.75	307.01	0	-4.32	down	0.04	1
circRNA6281	0	133.74	141.32	0	0	0	inf	up	0.04	1
circRNA13700	0	0	0	47.95	94.46	116.09	-inf	down	0.04	1
circRNA11030	0	0	0	47.95	94.46	116.09	-inf	down	0.04	1
circRNA15378	0	0	0	47.95	94.46	116.09	-inf	down	0.04	1
circRNA16503	0	0	0	47.95	94.46	116.09	-inf	down	0.04	1
circRNA51906	0	0	0	0	259.77	139.31	-inf	down	0.04	1
circRNA5945	0	53.50	0	215.75	212.54	162.53	-3.47	down	0.04	1
circRNA7774	0	0	56.53	215.75	236.16	139.31	-3.39	down	0.04	1
circRNA14001	0	0	0	95.89	118.08	46.44	-inf	down	0.04	1
circRNA16905	0	0	0	0	141.70	255.40	-inf	down	0.04	1
circRNA47487	0	0	0	95.89	47.23	116.09	-inf	down	0.04	1
circRNA3293	52.29	0	0	71.92	165.31	255.40	-3.24	down	0.04	1
circRNA27645	0	0	0	239.73	118.08	0	-inf	down	0.04	1
circRNA4129	52.29	0	0	71.92	118.08	325.06	-3.30	down	0.04	1
circRNA9607	0	0	0	119.86	47.23	92.87	-inf	down	0.04	1
circRNA24949	0	0	0	119.86	47.23	92.87	-inf	down	0.04	1
ciRNA648	0	0	0	119.86	47.23	92.87	-inf	down	0.04	1
circRNA8570	0	0	56.53	407.54	0	580.46	-4.13	down	0.04	1
circRNA9865	0	0	0	47.95	141.70	69.66	-inf	down	0.04	1
circRNA34	130.73	106.99	226.12	934.94	779.32	417.93	-2.20	down	0.04	1
circRNA6125	0	240.73	56.53	0	0	0	inf	up	0.04	1
circRNA4889	0	106.99	0	335.62	259.77	417.93	-3.24	down	0.05	1
circRNA10916	0	0	0	47.95	70.85	139.31	-inf	down	0.05	1
circRNA1585	339.89	240.73	310.91	119.86	94.46	162.53	1.24	up	0.05	1
circRNA2058	209.17	133.74	28.26	0	23.62	46.44	2.41	up	0.05	1
circRNA2966	52.29	133.74	0	239.73	330.62	766.21	-2.84	down	0.05	1
circRNA13686	0	0	0	191.78	47.23	46.44	-inf	down	0.05	1
circRNA11149	0	0	0	191.78	165.31	0	-inf	down	0.05	1

circRNA21061	0	0	0	0	236.16	139.31	-inf	down	0.05	1
circRNA3678	130.73	0	141.32	0	0	0	inf	up	0.05	1
circRNA9108	0	0	0	143.84	47.23	69.66	-inf	down	0.05	1
circRNA13750	0	0	0	143.84	47.23	69.66	-inf	down	0.05	1
circRNA16657	0	0	0	143.84	70.85	46.44	-inf	down	0.05	1
circRNA1450	156.87	106.99	0	0	0	0	inf	up	0.05	1
circRNA1853	392.18	53.50	113.06	743.15	614.01	1439.54	-2.32	down	0.05	1
circRNA751	287.60	133.74	113.06	71.92	23.62	46.44	1.91	up	0.05	1
circRNA3021	78.44	53.50	56.53	0	0	0	inf	up	0.05	1
circRNA3085	156.87	0	113.06	0	0	0	inf	up	0.05	1
circRNA13687	0	0	0	119.86	0	232.18	-inf	down	0.05	1
circRNA27642	0	0	0	143.84	0	185.75	-inf	down	0.05	1
circRNA13246	0	0	0	0	141.70	208.97	-inf	down	0.05	1
circRNA13616	0	0	0	191.78	0	139.31	-inf	down	0.05	1

Table S7 complete DEMs lists (Ud vs Sad)

miR_name	up/down	log_2fc	pvalue(t_test)	Sad_1(norm)	Sad_2(norm)	Sad_3(norm)	Ud_1(norm)	Ud_2(norm)	Ud_3(norm)
bta-miR-346	down	-1.95	0.00	26	26	28	6	7	8
mmu-miR-26a-2-3p_1ss4GA	up	3.07	0.00	0	5	8	34	34	40
bta-miR-484	down	-0.77	0.00	3,494	3,864	3,707	1,940	2,224	2,327
cgr-miR-1260_L+1R+1	down	-inf	0.00	6	6	6	0	0	0
bta-miR-141_R+1	up	3.87	0.00	6	0	0	23	27	31
bta-miR-423-5p	down	-1.45	0.00	13,154	15,694	13,374	5,455	5,987	3,979
bta-miR-9851	down	-inf	0.00	8	7	7	0	0	0
chi-miR-199a-5p	up	0.98	0.00	134,155	126,897	121,161	252,949	265,975	235,915
bta-miR-2478_L+2	down	-3.80	0.00	27	20	21	5	0	0
oga-miR-28b_R+1	up	1.79	0.00	166	180	82	563	477	436
hsa-miR-151b_R+4_2	up	1.79	0.00	166	180	82	563	477	436
hsa-miR-151b_R+4_1	up	1.79	0.00	166	180	82	563	477	436
bta-let-7a-3p_R+1	up	1.54	0.00	247	253	392	812	984	792
mmu-miR-145a-3p_R-1	up	1.90	0.00	7,016	3,769	10,219	22,505	29,597	26,314
ssc-mir-4335-p5_1ss18CT	up	inf	0.00	0	0	0	14	14	12
bta-miR-98	up	1.21	0.00	3,144	2,065	3,590	6,463	7,464	6,422
bta-miR-186	up	0.51	0.00	30,865	33,989	29,686	41,462	46,657	46,376
PC-3p-33596_118	down	-inf	0.00	6	6	5	0	0	0
bta-miR-6525_R+3_1ss2TC	down	-3.15	0.00	20	21	15	0	0	6
oar-miR-148a	up	0.91	0.00	59,169	65,610	83,067	114,498	136,389	138,896
efu-mir-9226-p5_1ss17GA	down	-0.71	0.01	61	63	54	35	39	34
chi-miR-191-3p	up	2.06	0.01	30	36	10	127	89	101
chi-miR-192-3p_R-2	down	-inf	0.01	10	10	8	0	0	0
bta-miR-129-3p	down	-2.36	0.01	453	630	626	163	76	94

hsa-miR-194-3p_R+1	down	-3.20	0.01	15	18	12	5	0	0
bta-miR-491_R+1	down	-1.14	0.01	385	343	295	155	131	179
chi-let-7d-3p_R+1	down	-1.44	0.01	5,109	5,130	3,776	2,302	1,745	1,121
bta-miR-199b	up	1.79	0.01	2,515	2,404	6,876	15,310	14,535	11,027
bta-miR-2284y_L+1R+1	down	-1.41	0.01	38	45	32	9	17	18
hsa-miR-574-5p	down	-2.85	0.01	4,747	3,562	3,598	744	365	542
bta-miR-1247-5p	down	-2.35	0.01	840	1,138	785	259	222	62
oar-miR-369-3p	up	1.30	0.01	375	229	455	787	1,034	788
chi-miR-877-3p_1ss22GT	down	-1.43	0.01	36	28	36	12	7	19
chi-miR-3432-3p	down	-0.72	0.01	192	238	201	150	107	125
PC-5p-12827_407	down	-1.18	0.01	26	35	27	10	16	13
cgr-miR-1260_R+3	down	-inf	0.01	5	6	7	0	0	0
pal-miR-9226-5p	down	-3.60	0.01	67	38	59	0	0	13
bta-miR-30b-3p_R+1	down	-1.04	0.01	77	89	106	53	41	38
hsa-miR-1260b_R-1_1ss9AG	down	-1.24	0.01	2,009	1,864	1,613	1,011	392	913
bta-miR-2332	up	2.07	0.01	0	5	7	21	14	15
bta-miR-2285n_L-1R+2_1ss21GT	up	inf	0.02	0	0	0	6	9	8
chi-miR-10b-3p_L-1	up	0.88	0.02	674	756	1,067	1,349	1,459	1,795
chi-miR-378-5p	down	-1.01	0.02	296	332	245	201	114	118
bta-miR-199a-3p_R+2	up	5.46	0.02	0	0	4	56	72	44
bta-miR-296-3p_L-1R+1	down	-1.30	0.02	983	1,164	1,185	439	239	677
bta-miR-320a	down	-1.43	0.02	53,319	64,867	47,756	19,711	20,407	21,441
chi-miR-1306-3p_1ss22GT	down	-1.64	0.02	29	40	46	14	8	16
cgr-miR-1260_L+1_1	down	-2.02	0.02	9	15	15	3	0	7
cgr-miR-1260_L+1_2	down	-2.02	0.02	9	15	15	3	0	7
cpo-miR-328-3p_2ss20TC22TC	down	-2.54	0.02	12	9	15	0	0	6
bta-mir-2285bm-p5	up	inf	0.02	0	0	0	6	7	9
bta-miR-214	down	-0.90	0.02	11,030	8,143	9,493	6,694	4,977	3,674
chi-miR-148b-5p	up	1.19	0.02	29	44	67	99	111	111
hsa-miR-320d_R+1_1ss19AT	down	-2.92	0.02	8	5	5	2	0	0
hsa-miR-320c_1ss19GT	down	-2.92	0.02	8	5	5	2	0	0
oar-miR-154b-3p_L-1	up	inf	0.02	0	0	0	8	9	5
chi-miR-125a-3p	down	-0.56	0.03	233	248	294	197	157	172
bta-miR-331-3p_R+1	down	-1.06	0.03	4,879	6,711	7,174	3,344	2,304	3,357
bta-miR-1306	down	-1.14	0.03	296	426	315	183	118	170
oar-miR-30b_R+1	up	0.80	0.03	11,786	8,708	12,520	17,675	17,077	22,924
cpo-miR-99b-3p_R+1	down	-0.71	0.03	256	279	289	173	205	125
oar-miR-218a	up	1.03	0.03	2,463	1,222	2,469	3,604	5,169	3,826
bta-miR-500	down	-1.59	0.03	1,989	2,094	1,334	632	474	688
hsa-miR-136-3p	up	1.83	0.03	429	797	804	1,986	3,135	2,082

PC-3p-9091_609	up	1.74	0.03	6	16	37	50	61	86
bta-miR-24_R-2	up	1.45	0.03	113	51	107	173	289	279
hsa-miR-1260a_R+3_1ss9TG	down	-1.43	0.03	26	34	40	13	12	12
bta-miR-744_R-1	down	-1.09	0.03	580	907	722	378	212	449
bta-miR-2447	up	inf	0.03	0	0	0	12	7	7
pal-miR-9993a-3p	down	-0.50	0.03	79	71	90	66	50	53
bta-miR-423-3p_L-1	down	-0.84	0.03	22,898	27,760	20,390	14,069	12,308	13,335
hsa-miR-574-3p	down	-1.29	0.03	32,243	29,087	19,231	15,250	6,941	10,669
oar-miR-3959-3p	up	0.52	0.03	52	72	75	93	89	104
mmu-miR-218-1-3p_R-1	down	-1.34	0.04	68	63	64	42	18	17
chi-miR-532-3p_R+1	down	-1.23	0.04	2,369	3,355	2,155	1,490	848	1,016
bta-miR-1249	down	-1.16	0.04	947	1,017	973	475	612	227
chi-miR-204-3p_R+2	down	-0.87	0.04	38	31	27	21	10	21
bta-miR-193a-3p_R-2	down	-0.68	0.04	593	661	801	351	540	393
bta-miR-504	down	-1.82	0.04	1,239	1,883	1,076	466	554	170
mmu-miR-452-3p_1ss20GA	up	1.77	0.04	156	95	210	470	406	695
chi-let-7b-3p	down	-1.10	0.04	1,437	1,145	907	649	569	408
bta-miR-24-3p_R-2	up	0.81	0.04	11,668	7,068	12,688	17,582	18,954	18,375
bta-miR-197	down	-2.24	0.04	8,640	11,478	6,163	2,424	1,955	1,189
mdo-let-7f-2-3p_1ss22CT	up	1.02	0.04	56	103	67	115	184	159
oar-miR-136_R-1	up	1.64	0.04	15	38	87	132	110	193
bta-miR-760-3p_R+2	down	-1.22	0.04	126	106	173	55	77	41
bta-miR-126-5p	up	2.29	0.04	5,122	2,649	5,736	19,394	29,881	16,912
pal-miR-9226-5p_L-4	down	-1.70	0.04	39	24	39	11	0	21
chi-let-7f-3p_1ss22CT	up	0.33	0.04	47	45	55	55	65	64
bta-miR-30f_R-1	down	-0.70	0.04	6,368	6,203	4,972	3,765	4,534	2,483
chi-let-7e-3p	down	-0.67	0.04	745	940	687	540	374	579
PC-3p-43326_76	down	-inf	0.04	10	5	6	0	0	0
bta-miR-450b_R-1	up	1.92	0.04	1,752	1,132	1,659	4,700	4,834	7,692
oar-miR-191	down	-1.09	0.04	88,115	103,286	64,211	46,772	40,395	32,581
bta-miR-6119-3p_L-1_1ss23AT	up	1.60	0.05	79	29	62	164	123	228
bta-miR-628_R+1	up	1.26	0.05	11	8	21	22	34	39
bta-miR-877_R+2	down	-2.97	0.05	40	63	83	11	8	5
chi-miR-214-5p	up	1.08	0.05	311	182	114	441	328	515
chi-let-7g-3p_1ss22CT	down	-1.00	0.05	116	89	72	30	46	63
PC-5p-20319_236	down	-3.81	0.05	38	34	16	0	0	6
hsa-miR-320b_1ss20CA	down	-1.56	0.05	723	827	482	230	229	231
hsa-miR-301a-5p	up	2.11	0.05	13	7	11	37	37	62
bta-miR-6123	down	-1.80	0.05	35	44	63	30	0	10

Table S8 complete DEMs lists (Som vs Stm)

miR_name	up/down	log_2fc	pvalue(t_test)	Som_1(norm)	Som_2(norm)	Som_3(norm)	Stm_1(norm)	Stm_2(norm)	Stm_3(norm)
PC-5p-17912_273	up	6.10	0.00	129	124	123	5	0	0
oar-miR-541-5p	up	3.33	0.00	216	218	208	22	23	19
oar-let-7g_R+1	down	-0.93	0.00	58,914	60,502	55,888	111,817	113,585	107,460
oar-miR-380-3p	up	2.26	0.00	6,570	6,379	5,930	1,013	1,569	1,347
mmu-miR-300-3p_R-2	up	0.89	0.00	58	62	59	32	31	34
bta-miR-767_R-1	up	5.15	0.00	138	137	126	5	6	0
oar-miR-370-3p_R-2	up	2.69	0.00	4,574	4,208	4,101	825	574	590
oar-miR-154a-3p	up	1.65	0.00	183	166	165	54	63	47
oar-miR-299-5p	up	2.44	0.00	792	808	769	108	187	143
oar-miR-409-3p_L-1	up	1.94	0.00	6,492	6,386	6,287	1,945	1,554	1,489
hsa-miR-320d_L-1R+1	up	inf	0.00	2	2	2	0	0	0
bta-miR-181b_R-1	up	1.59	0.00	11,980	10,530	10,953	3,273	4,286	3,528
chi-miR-532-3p_R+1	up	1.74	0.00	3,188	3,391	3,150	1,183	961	763
bta-miR-542-5p_R+1	up	2.50	0.00	1,395	1,250	1,415	120	356	242
oar-miR-494-3p	up	1.59	0.00	10,486	11,093	9,620	2,821	3,907	3,646
hsa-miR-135a-3p_L+1R-1	up	3.81	0.00	129	122	128	0	12	15
sha-miR-125a_R+2	up	1.66	0.00	563	494	530	151	190	160
oar-miR-411a-3p	up	1.44	0.00	2,523	2,592	2,386	760	1,079	935
chi-miR-181d	up	1.95	0.00	2,985	2,737	2,906	789	738	703
mml-miR-503-3p_2ss21AC23GA	up	2.23	0.00	66	64	60	8	17	15
oar-miR-485-5p	up	2.26	0.00	2,004	2,018	2,048	515	394	359
bta-miR-500	up	1.53	0.00	1,765	1,718	1,985	752	612	536
oar-mir-329b-p5	up	inf	0.00	32	32	30	0	0	0
hsa-miR-431-3p_L+2_1ss21TG	up	3.80	0.00	48	40	47	5	4	0
oar-miR-154b-5p	up	2.10	0.00	1,282	1,325	1,110	386	237	245
oar-miR-134-3p_R-1	up	4.21	0.00	48	45	50	8	0	0
bta-miR-214	up	1.62	0.00	20,154	22,957	22,053	6,275	7,706	7,165
PC-3p-2996_2464	up	2.57	0.00	420	400	444	30	106	77
bta-miR-149-5p	up	2.48	0.00	1,642	1,630	1,362	459	187	183
oar-miR-323c	up	2.01	0.00	359	327	310	120	65	62
oar-miR-487a-3p_L+1R-1	up	2.48	0.00	101	109	98	16	19	20
eca-miR-876-3p_L-3R-1	up	3.01	0.00	40	36	32	0	8	5
oar-miR-487b-3p	up	1.86	0.00	1,978	2,073	1,792	555	596	459
oar-miR-432_1ss23GT	up	1.47	0.00	2,654	2,919	2,638	744	1,212	1,010
oar-miR-3957-3p_R-1	up	2.65	0.00	108	108	87	5	26	16

bta-mir-502b-p5_1ss18GA	up	inf	0.00	20	22	22	0	0	0
bta-miR-2331-5p	up	1.91	0.00	39	36	35	13	7	9
oar-miR-668-3p_L-1	up	4.56	0.00	71	80	67	0	4	5
bta-miR-503-3p_1ss14TC	up	1.76	0.00	172	184	211	37	72	58
chi-miR-125b-3p_R+1	up	0.54	0.00	9,505	8,694	9,294	6,768	5,958	6,207
bta-miR-10172-3p_R-1	up	inf	0.00	29	28	26	0	0	0
oar-miR-125b_R+1	up	0.91	0.00	1,035,270	943,048	910,122	523,996	543,988	466,595
oar-miR-433-3p	up	2.62	0.00	1,564	1,520	1,356	249	267	204
bta-miR-187_R+1	up	3.63	0.00	95	76	74	20	0	0
chi-miR-342-5p_R-2	up	0.93	0.00	158	145	138	78	88	66
oar-miR-381-3p_L-1R+2_1ss5AG	up	0.90	0.00	616	614	633	359	341	301
oar-miR-411b-3p_1ss6CA	up	1.60	0.00	75	82	66	19	32	22
PC-3p-18281_268	up	1.37	0.00	25	30	29	11	8	13
chi-miR-202-3p_R+1	up	3.36	0.00	2,246	2,309	2,251	32	349	281
hsa-miR-181a-2-3p_1ss6TC	up	1.42	0.00	1,243	1,159	1,285	589	391	395
oar-miR-410-3p	up	1.93	0.00	1,832	1,989	1,648	428	566	438
mmu-let-7c-5p_R+2	up	1.01	0.00	187	174	180	76	100	92
oar-miR-370-5p	up	3.45	0.00	16	17	21	0	5	0
oar-miR-665-5p_R-2	up	1.97	0.00	137	150	115	52	26	25
chi-miR-335-3p	up	0.70	0.00	405	420	403	235	245	276
PC-5p-39537_90	up	3.81	0.00	35	31	43	8	0	0
bta-miR-483_R+2	up	3.75	0.00	784	854	740	59	55	63
bta-miR-342_R-2	up	0.63	0.00	27,360	30,490	30,646	18,477	20,576	18,072
oan-miR-23b-3p_R-4_1ss17TA	down	-2.65	0.00	6	2	7	30	37	29
hsa-miR-432-3p	up	3.39	0.00	44	33	38	11	0	0
bta-miR-193a-3p_R-2	down	-1.30	0.00	97	129	150	332	324	269
pal-mir-9226-p5_1ss18CT	up	1.15	0.00	92	82	83	48	36	31
hsa-miR-320b_1ss20CA	up	1.07	0.00	660	705	657	264	374	322
bta-miR-455-3p_R-1	up	1.68	0.00	21,749	20,387	18,527	6,881	6,044	6,032
oar-miR-758-3p_R+1	up	1.99	0.00	763	794	755	121	260	201
oar-miR-665-3p_R+1	up	2.56	0.00	2,184	2,291	2,231	620	214	300
hsa-miR-320b	up	1.01	0.00	392	376	334	193	187	166
hsa-miR-194-3p_R+1	up	3.31	0.00	17	21	23	0	0	6
bta-miR-181c_R-2	up	1.94	0.00	1,635	1,551	1,340	401	442	338

chi-miR-27b-5p	down	-1.25	0.00	32	42	32	76	94	83
bta-miR-363_L+1	up	1.59	0.00	268	316	266	76	138	69
oar-miR-409-5p	up	1.50	0.00	4,319	4,210	4,804	1,554	1,632	1,529
oar-miR-379-3p_R-1	up	1.28	0.00	1,612	1,580	1,497	485	766	681
bta-miR-125a_R-1	up	0.96	0.00	179,843	179,665	154,068	107,680	82,957	73,683
mmu-miR-92a-3p_R+1	up	1.11	0.00	665	675	630	373	270	273
oar-miR-30a-5p_R+1	down	-1.11	0.00	23,546	21,959	23,617	53,560	49,060	46,431
oar-miR-127_L-1	up	1.35	0.00	52,408	48,532	59,130	13,506	27,473	21,960
oar-miR-655-3p	up	1.70	0.00	1,134	1,287	966	262	453	330
bta-miR-24-3p_R-2	down	-1.10	0.00	4,936	5,552	4,833	11,393	11,591	9,812
oar-mir-495-p5	up	2.58	0.00	105	95	85	16	15	16
hsa-miR-383-3p_L+2	up	inf	0.00	9	9	11	0	0	0
oar-miR-543-3p_R-1	up	1.68	0.01	1,061	1,130	844	236	398	313
hsa-miR-378d	down	-2.30	0.01	10	8	11	48	53	41
bta-miR-105a	up	6.04	0.01	91	78	104	0	4	0
oar-miR-362_R-2	up	1.02	0.01	3,607	4,109	3,878	1,440	2,319	1,942
bta-miR-125b_R+1	up	1.41	0.01	3,800	3,278	3,290	1,344	1,279	1,279
bta-miR-2285cs_R-1	up	5.65	0.01	22	20	27	0	1	0
PC-3p-2770_2765	down	-2.16	0.01	52	37	39	219	175	175
bta-miR-873_L+1R-1	up	inf	0.01	15	16	20	0	0	0
chi-miR-181c-3p	up	1.44	0.01	532	491	480	259	141	153
bta-miR-7	down	-1.65	0.01	174	171	182	578	489	590
chi-miR-330-5p_R+2	up	0.91	0.01	143	124	114	82	64	57
bta-miR-151-3p	down	-0.49	0.01	7,424	7,197	7,115	10,812	9,946	9,824
oar-miR-221	down	-3.20	0.01	872	997	785	9,271	8,004	7,086
bta-miR-507b_R-1_1ss 22GA	down	-1.96	0.01	171	191	176	606	753	735
chi-miR-18a-3p_R-2	up	1.82	0.01	47	53	39	5	22	11
chi-miR-146b-3p	up	3.50	0.01	353	264	286	16	36	28
chi-miR-127-5p_L+2R -1	up	1.24	0.01	6,341	6,138	6,924	1,777	3,432	2,992
hsa-miR-181a-3p	up	1.10	0.01	258	221	258	111	116	117
bta-miR-2285k_L-1R+ 1	down	-inf	0.01	0	0	0	15	19	15
hsa-miR-320c_L-1R+1 _1ss20TA	up	0.93	0.01	15	14	14	8	9	6
oar-miR-134-5p_R+1	up	1.77	0.01	286	265	269	41	114	86
PC-3p-99783_12	up	inf	0.01	1	2	2	0	0	0
hsa-miR-299-3p_1ss10 TC	up	1.73	0.01	2,257	2,175	2,224	379	891	736
dno-miR-432-5p_L-2R +3_2	up	inf	0.01	9	12	11	0	0	0

dno-miR-432-5p_L-2R+3_1	up	inf	0.01	9	12	11	0	0	0
bta-miR-10172-5p	up	inf	0.01	5	5	4	0	0	0
hsa-miR-4454_L-3R+1	up	2.92	0.01	7	6	5	0	2	0
oar-miR-376e-3p	up	1.33	0.01	565	605	692	148	349	244
oar-miR-329a-3p	up	2.89	0.01	12	13	18	0	6	0
hsa-miR-9983-3p	up	2.05	0.01	54	65	48	0	20	21
PC-5p-82157_19	up	inf	0.01	3	3	4	0	0	0
bta-miR-3154_1ss13CT	up	inf	0.01	4	4	3	0	0	0
bta-miR-20b_R+1	up	0.56	0.01	48	54	49	33	36	34
oar-miR-329b-3p_R-2	up	1.47	0.01	1,630	1,735	1,239	475	721	466
oan-miR-23b-3p_R-3_1ss17TA	down	-1.83	0.01	3	2	3	10	10	8
oar-miR-377-5p	up	3.17	0.01	82	78	57	5	12	6
bta-miR-1814c_R-2	down	-1.38	0.01	4	5	4	12	10	12
hsa-miR-320b_R-1_1ss20CA	up	0.85	0.01	339	301	299	131	207	183
bta-miR-424-3p	up	1.68	0.01	7,125	7,220	6,464	1,020	3,272	2,203
oar-miR-323a-3p_L+1	up	1.51	0.01	167	172	159	32	77	66
hsa-miR-4454_L-2R+1	up	inf	0.01	4	3	4	0	0	0
bta-miR-224_R-1	down	-0.64	0.01	5,712	6,050	5,893	9,372	9,748	8,365
chi-miR-1185-3p	up	1.45	0.01	41	51	35	10	23	13
hsa-miR-106b-3p	up	0.40	0.01	2,859	2,510	2,853	2,253	2,035	1,961
bta-miR-2284k_R+2_1ss11GT	down	-0.90	0.01	20	14	14	25	33	32
bta-miR-335_R-2	up	1.20	0.01	4,185	4,376	4,134	1,327	2,387	1,824
dno-miR-134-5p_R+1_1ss22CA	up	2.26	0.01	34	48	39	0	19	6
PC-3p-9091_609	down	-0.87	0.01	28	40	26	62	53	58
oar-miR-412-3p	up	1.29	0.01	196	174	201	47	109	77
mmu-let-7j_1ss8TG	down	-0.22	0.01	409	388	424	482	471	470
chi-let-7e-3p	up	1.30	0.01	1,365	1,377	1,123	793	374	399
mdo-miR-181a-5p_R+3_2	up	1.26	0.01	57	81	79	23	41	28
mdo-miR-181a-5p_R+3_1	up	1.26	0.01	57	81	79	23	41	28
oar-miR-493-5p	up	1.39	0.01	4,232	4,512	4,254	1,013	2,275	1,679
bta-miR-140_L-1R+1	up	0.74	0.01	63,909	61,582	66,742	30,479	43,615	40,784
bta-miR-93_R+1	down	-0.44	0.01	5,972	5,578	5,637	8,266	7,882	7,165
hsa-miR-320d_R+2	up	inf	0.01	1	2	2	0	0	0
PC-3p-16460_302	down	-2.79	0.01	0	0	3	7	8	7

oar-miR-374b	up	0.41	0.02	4,289	4,131	4,659	3,381	3,576	2,892
chi-miR-15b-3p	down	-1.26	0.02	17	15	15	33	44	37
chi-miR-188-3p	up	2.06	0.02	18	22	21	5	9	0
oar-miR-181a	up	1.19	0.02	22,927	24,400	20,746	6,328	14,023	9,515
oar-miR-27a_R-1	down	-1.24	0.02	5,597	6,740	5,992	15,267	16,086	12,025
mmu-miR-5100_L-1R-1	up	inf	0.02	3	2	2	0	0	0
oar-miR-493-3p	up	1.30	0.02	1,539	1,488	1,350	315	802	656
oar-miR-369-5p	up	1.50	0.02	121	140	90	24	65	36
oar-miR-3957-5p	up	3.03	0.02	22	41	35	12	0	0
chi-miR-30d-3p_L+1	down	-1.39	0.02	31	31	29	74	93	73
hsa-miR-574-5p	up	1.08	0.02	4,632	5,174	4,304	3,100	1,515	2,053
PC-3p-36930_101	up	2.77	0.02	8	8	7	3	0	0
hsa-mir-378c-p5	down	-1.86	0.02	30	23	19	108	80	75
bta-miR-378c_1ss21TA	down	-1.86	0.02	30	23	19	108	80	75
bta-miR-2447	up	inf	0.02	40	30	26	0	0	0
oan-miR-23b-3p_R-3	down	-1.59	0.02	13	17	20	40	55	58
bta-miR-16a_R+1_1ss21TA	down	-0.64	0.02	23,627	24,778	22,149	36,712	40,592	32,909
oar-let-7f_R+1	down	-0.96	0.02	88,098	86,605	86,246	150,673	189,742	165,757
hsa-miR-500a-3p_R-1	up	1.08	0.02	4,706	4,247	4,786	3,003	1,598	1,917
mmu-miR-92a-3p_R+2	up	1.18	0.02	250	207	242	153	81	75
oar-miR-495-3p	up	1.20	0.02	1,495	1,574	1,522	415	851	738
bta-mir-10173-p3_1ss16CT	up	inf	0.02	7	5	5	0	0	0
oar-miR-487a-5p	up	2.76	0.02	12	10	6	0	4	0
oar-miR-485-3p	up	2.31	0.02	1,022	1,014	657	249	133	161
PC-5p-29736_142	up	2.91	0.02	36	19	26	0	11	0
hsa-miR-320d_1ss18GA	up	0.67	0.02	92	86	83	45	65	54
oar-miR-323b	up	inf	0.02	4	4	3	0	0	0
bta-miR-184	up	inf	0.02	8	5	6	0	0	0
bta-miR-451_R-2	down	-1.00	0.02	172	197	219	341	464	373
bta-mir-11986-p3_1ss17AG	up	inf	0.02	15	11	19	0	0	0
hsa-miR-26a-1-3p_1ss9TC	down	-2.57	0.02	0	3	0	7	7	5
ssc-miR-7134-3p_1ss7AC	up	1.38	0.02	249	234	230	142	62	71
bta-miR-1246_L+1R+1	down	-1.12	0.02	8	11	16	30	26	20

bta-miR-2474	up	inf	0.02	6	4	4	0	0	0
hsa-miR-221-5p	down	-3.43	0.02	57	46	73	813	513	571
bta-miR-92a	up	0.90	0.02	71,400	70,390	67,773	48,472	32,226	31,570
bta-miR-185	down	-1.11	0.03	2,299	2,261	2,112	5,599	4,063	4,784
bta-let-7f_R+1	down	-0.69	0.03	197	165	204	254	334	321
chi-miR-128-5p_R-2	up	1.12	0.03	35	32	31	22	10	13
hsa-miR-365b-5p_L+1	down	-inf	0.03	0	0	0	9	5	6
bta-miR-378_R+1_1ss22CT	down	-2.46	0.03	10	3	6	47	32	28
chi-miR-125a-3p	up	0.93	0.03	455	453	396	317	181	187
oan-miR-27b-3p_R+1	down	-1.17	0.03	31	32	33	64	86	67
oar-let-7i	down	-0.73	0.03	48,574	50,035	47,552	70,727	90,468	80,845
ocu-miR-513-5p_R-2	down	-1.13	0.03	3	7	10	15	12	15
efu-mir-9298-p3_1ss2TG	down	-0.76	0.03	54	56	68	119	89	93
oan-miR-27b-3p_R+2	down	-0.96	0.03	35	30	37	53	70	75
oar-miR-382-5p	up	1.19	0.03	1,908	2,051	1,774	431	1,227	853
hsa-miR-105-3p_L+2R-2	up	inf	0.03	17	9	15	0	0	0
chi-miR-105b-3p	up	inf	0.03	17	9	15	0	0	0
bta-miR-378d_R-3	down	-2.85	0.03	2	2	5	25	14	23
hsa-miR-212-3p_R+1	up	0.68	0.03	68	64	60	47	29	43
bta-miR-1246_L-1R+1	down	-inf	0.03	0	0	0	3	5	3
oar-miR-23b_R+4_2	down	-0.89	0.04	18,143	18,195	17,292	38,896	29,372	30,811
oar-miR-23b_R+4_1	down	-0.89	0.04	18,143	18,195	17,292	38,896	29,372	30,811
bta-let-7a-3p_R+1	down	-1.03	0.04	261	281	235	424	542	617
bta-miR-2285ad_L-1_1ss8AG	up	0.92	0.04	62	55	54	42	27	22
bta-miR-378c_2ss20GA21TA	down	-1.89	0.04	4	3	7	12	19	23
hsa-miR-132-5p	up	1.62	0.04	23	14	19	0	10	8
PC-3p-56454_44	up	inf	0.04	25	18	12	0	0	0
hsa-miR-7977_R+4_1ss6AG	up	1.85	0.04	22	15	21	12	4	0
oar-miR-107_R-1	up	0.30	0.04	6,406	6,557	5,599	5,351	5,053	4,642
bta-miR-2448-3p	up	0.89	0.04	75	99	68	54	46	31
oar-miR-30b_R+1	down	-0.76	0.04	5,722	6,395	5,730	11,803	9,621	8,794
bta-miR-105b	up	inf	0.04	42	24	24	0	0	0
cgr-miR-532-5p_R+2_2	up	1.57	0.04	15	15	21	0	9	8
cgr-miR-532-5p_R+2_1	up	1.57	0.04	15	15	21	0	9	8

oar-miR-30a-3p_R+1	down	-1.30	0.04	360	335	302	632	987	836
bta-miR-2285k_L-1R+2_1ss7GC_2	down	-1.77	0.04	8	7	7	20	32	22
bta-miR-378	down	-2.52	0.04	1,891	1,895	1,876	14,544	8,220	9,683
chi-miR-1306-3p_1ss22GT	up	1.13	0.04	74	92	61	37	32	34
eca-miR-483	up	inf	0.04	10	7	14	0	0	0
bta-miR-532	up	0.91	0.04	15,197	14,506	15,723	4,924	10,358	8,945
bta-miR-2285bj_1ss1CA	up	inf	0.04	1	1	2	0	0	0
bta-miR-2285ba_1ss11CT	up	inf	0.04	1	1	2	0	0	0
chi-miR-873-3p_R+1	up	inf	0.04	6	3	7	0	0	0
cfa-miR-486_R+3	up	1.35	0.04	37	44	38	18	4	25
bta-miR-146b	up	1.38	0.04	3,042	2,598	2,925	249	1,639	1,397
cja-miR-3546_R+2	up	inf	0.04	4	8	4	0	0	0
mdo-miR-181b-5p_R+3	up	inf	0.05	14	6	13	0	0	0
bta-miR-222_R+2	down	-2.90	0.05	520	589	551	5,705	3,141	3,568
bta-miR-142-5p_L+2R-1	down	-3.46	0.05	244	290	265	4,114	2,343	2,339
PC-3p-13885_370	down	-1.63	0.05	15	18	23	75	55	42
oar-miR-26a	down	-0.51	0.05	364,289	362,968	331,011	439,662	570,298	497,012
mmu-mir-3968-p5_1ss12AT	up	inf	0.05	4	2	2	0	0	0
bta-mir-3431-p3	down	-1.02	0.05	211	217	197	521	382	361
PC-3p-33085_120	up	inf	0.05	10	6	4	0	0	0
bta-miR-361	up	0.73	0.05	16,185	15,711	14,210	12,467	7,243	8,167

Table S9 complete DEMs lists (Stm vs Sad)

miR_name	up/down	log_2fc	pvalue(t_test)	Sad_1(norm)	Sad_2(norm)	Sad_3(norm)	Stm_1(norm)	Stm_2(norm)	Stm_3(norm)
bta-miR-2285bz_R+1	up	inf	0.00	0	0	0	8	7	7
mmu-let-7c-5p_R+2	down	-0.91	0.00	152	185	167	76	100	92
ocu-miR-513-5p_R-2	up	inf	0.00	0	0	0	15	12	15
bta-miR-507b_R-1_1ss22GA	up	2.25	0.01	110	43	286	606	753	735
bta-miR-1814c_R-2	up	1.13	0.01	7	5	4	12	10	12
PC-3p-2770_2765	up	2.44	0.01	13	12	79	219	175	175
chi-miR-192-3p_R-2	down	-inf	0.01	10	10	8	0	0	0
oar-miR-107_R-1	up	0.53	0.01	3,179	3,294	3,960	5,351	5,053	4,642
bta-miR-1247-5p	down	-2.11	0.01	840	1,138	785	344	146	148
PC-3p-15354_329	down	-2.92	0.01	11	12	8	0	4	0
hsa-miR-194-3p_R+1	down	-2.86	0.01	15	18	12	0	0	6

oan-miR-145-5p_R+1_1ss19TA	down	-2.57	0.01	237	144	189	4	61	30
oar-let-7g_R+1	up	0.32	0.01	93,464	81,515	91,499	111,817	113,585	107,460
bta-miR-193a-3p_R-2	down	-1.15	0.02	593	661	801	332	324	269
oar-miR-362_R-2	down	-1.10	0.02	4,324	4,657	3,238	1,440	2,319	1,942
hsa-miR-32-5p_R-1	up	1.72	0.02	38	42	87	156	236	159
oar-miR-487a-3p_L+1R-1	down	-0.48	0.02	27	23	27	16	19	20
bta-miR-145	down	-1.57	0.02	361,549	233,552	377,870	53,241	154,574	120,091
bta-miR-98	up	0.89	0.02	3,144	2,065	3,590	4,826	6,485	4,970
bta-miR-2285n_L-1R+2_1ss21GT	up	inf	0.02	0	0	0	5	5	3
PC-5p-12815_407	up	1.87	0.03	11	15	0	24	41	32
chi-miR-204-3p_R+2	down	-0.84	0.03	38	31	27	14	19	21
bta-miR-199b	up	2.48	0.03	2,515	2,404	6,876	14,841	27,037	23,915
oar-let-7i	up	0.55	0.03	51,692	49,596	64,206	70,727	90,468	80,845
PC-3p-13885_370	up	1.85	0.03	20	0	27	75	55	42
bta-miR-500	down	-1.51	0.03	1,989	2,094	1,334	752	612	536
bta-miR-128	up	0.63	0.03	1,045	1,048	735	1,583	1,582	1,221
mmu-miR-1983	up	3.63	0.03	0	7	0	35	36	17
PC-3p-16460_302	down	-2.21	0.03	30	28	44	7	8	7
bta-miR-2284y_L+1R+1	down	-0.85	0.03	38	45	32	22	23	19
chi-miR-532-3p_R+1	down	-1.44	0.04	2,369	3,355	2,155	1,183	961	763
bta-let-7a-3p_R+1	up	0.83	0.04	247	253	392	424	542	617
PC-3p-6743_861	up	1.70	0.04	7	8	14	41	25	27
bta-miR-342_R-2	down	-0.61	0.04	33,788	26,897	26,773	18,477	20,576	18,072
oar-miR-3955-5p	up	1.11	0.04	21	33	43	51	79	79
bta-miR-132	down	-0.48	0.04	364	376	320	301	246	213
oar-miR-26a	down	-0.74	0.04	959,980	885,070	679,463	439,662	570,298	497,012
oar-let-7f_R+1	up	0.49	0.04	116,073	102,999	142,160	150,673	189,742	165,757
PC-5p-5825_1028	down	-1.84	0.04	70	90	46	5	26	26
bta-miR-2285bp_1ss19GT	up	1.29	0.04	13	8	28	52	36	34
hsa-miR-574-5p	down	-0.84	0.05	4,747	3,562	3,598	3,100	1,515	2,053
PC-3p-9091_609	up	1.54	0.05	6	16	37	62	53	58
mdo-miR-26-5p_R+3	down	-0.69	0.05	64	56	64	45	44	26

Table S10 complete DEMs lists (Som vs Sad)

miR_name	up/down	log_2fc	pvalue(t_test)	Sad_1(norm)	Sad_2(norm)	Sad_3(norm)	Som_1(norm)	Som_2(norm)	Som_3(norm)
chi-miR-202-3p_R+1	up	3.81	0.00	164	138	184	2,246	2,309	2,251
oar-miR-495-3p	up	1.19	0.00	710	640	658	1,495	1,574	1,522
bta-miR-202	up	3.26	0.00	744	340	824	5,861	5,972	6,407
oar-miR-432_1ss23GT	up	2.35	0.00	413	482	713	2,654	2,919	2,638

chi-miR-181d	up	2.12	0.00	578	699	705	2,985	2,737	2,906
bta-miR-767_R-1	up	5.26	0.00	6	0	5	138	137	126
oar-miR-3958-3p	up	2.01	0.00	327	428	369	1,605	1,500	1,435
oar-miR-494-3p	up	1.96	0.00	1,906	3,082	3,039	10,486	11,093	9,620
oar-miR-433-3p	up	2.77	0.00	162	300	190	1,564	1,520	1,356
oar-miR-323a-3p_L+1	up	1.96	0.00	35	54	39	167	172	159
oar-miR-409-5p	up	2.49	0.00	431	1,153	789	4,319	4,210	4,804
hsa-miR-4454_L+1_1ss3GA	up	1.70	0.00	8	10	7	26	26	29
bta-miR-542-5p_R+1	up	2.28	0.00	320	297	216	1,395	1,250	1,415
oar-miR-382-5p	up	1.68	0.00	537	731	518	1,908	2,051	1,774
chi-miR-181c-3p	up	2.05	0.00	84	160	118	532	491	480
oar-miR-487a-3p_L+1R-1	up	2.00	0.00	27	23	27	101	109	98
pal-mir-9226-p5_1ss18CT	up	1.32	0.00	33	40	29	92	82	83
oar-miR-323c	up	2.44	0.00	30	90	63	359	327	310
bta-miR-138_R+1	up	4.61	0.00	12	0	6	139	135	157
oar-miR-410-3p	up	2.51	0.00	180	353	429	1,832	1,989	1,648
cpo-miR-99b-3p_R+1	up	0.85	0.00	256	279	289	511	511	468
oar-miR-668-3p_L-1	up	3.98	0.00	7	7	0	71	80	67
bta-miR-214	up	1.18	0.00	11,030	8,143	9,493	20,154	22,957	22,053
bta-miR-504	up	1.81	0.00	1,239	1,883	1,076	5,269	4,467	5,020
oar-miR-412-3p	up	2.68	0.00	22	32	35	196	174	201
oar-miR-487b-3p	up	2.00	0.00	487	564	412	1,978	2,073	1,792
oar-miR-370-3p_R-2	up	2.13	0.00	807	1,415	711	4,574	4,208	4,101
bta-miR-135b	up	inf	0.00	0	0	0	38	36	39
oar-miR-544-5p	up	1.45	0.00	70	101	88	245	249	215
bta-miR-199a-3p_R+2	up	3.88	0.00	0	0	4	21	19	18
bta-miR-483_R+2	up	1.89	0.00	177	298	164	784	854	740
hsa-miR-1260a_L+1_1ss10TG	up	inf	0.00	0	0	0	2	2	2
bta-miR-496_R-1	up	3.03	0.00	0	5	6	30	27	33
oar-miR-493-3p	up	1.73	0.00	434	577	313	1,539	1,488	1,350
bta-miR-146b	up	2.63	0.00	586	404	393	3,042	2,598	2,925
bta-miR-149-5p	up	2.39	0.00	251	415	218	1,642	1,630	1,362
eca-miR-876-3p_L-3R-1	up	3.79	0.00	0	0	8	40	36	32
bta-miR-744_R-1	up	1.37	0.00	580	907	722	2,046	1,804	1,845
hsa-miR-28-3p_1ss11TA	down	-1.10	0.00	9,548	8,782	9,166	4,433	4,240	4,196
bta-miR-484	up	0.68	0.00	3,494	3,864	3,707	6,230	5,894	5,650

hsa-miR-210-5p_R+1_ 1ss5CA	up	3.16	0.00	6	0	17	65	61	73
bta-miR-2331-5p	up	3.08	0.00	0	6	7	39	36	35
oar-miR-409-3p_L-1	up	2.48	0.00	774	1,568	1,090	6,492	6,386	6,287
hsa-miR-30c-1-3p	up	0.86	0.00	83	92	72	149	142	157
PC-3p-42302_79	up	3.73	0.00	0	0	5	25	22	19
bta-miR-7862	up	2.93	0.00	0	5	10	43	33	37
oar-miR-411a-3p	up	1.47	0.00	849	1,128	734	2,523	2,592	2,386
bta-miR-1185	up	1.86	0.00	8	14	7	33	33	39
chi-miR-324-3p_1ss21 GT	up	1.29	0.00	108	103	154	303	315	273
bta-miR-491_R+1	up	1.05	0.00	385	343	295	693	731	699
bta-miR-378d_R-3	down	-2.33	0.00	12	16	15	2	2	5
oar-miR-376c-3p	up	1.35	0.00	183	114	164	367	371	438
bta-miR-6123	up	1.97	0.00	35	44	63	182	181	190
oar-miR-3957-3p_R-1	up	2.66	0.00	9	21	19	108	108	87
oar-miR-380-3p	up	2.02	0.00	1,604	2,192	868	6,570	6,379	5,930
bta-miR-182	up	3.49	0.00	23	11	18	180	191	217
bta-mir-7865-p3_1ss21 GT	up	1.57	0.00	15	30	22	73	64	61
oar-miR-411b-5p	up	2.35	0.00	11	17	44	110	140	121
mdo-miR-26-5p_R+3	down	-0.75	0.00	64	56	64	32	36	41
mmu-miR-218-1-3p_R -1	down	-1.99	0.00	68	63	64	20	9	21
hsa-miR-135a-3p_L+1 R-1	up	3.50	0.00	9	25	0	129	122	128
oar-miR-369-3p	up	1.54	0.00	375	229	455	1,008	1,163	918
PC-5p-39537_90	up	4.13	0.00	0	6	0	35	31	43
hsa-miR-450a-1-3p_1s s8AG	up	1.74	0.00	41	20	24	107	83	94
oar-miR-154b-5p	up	1.85	0.00	340	387	307	1,282	1,325	1,110
oar-miR-107_R-1	up	0.83	0.00	3,179	3,294	3,960	6,406	6,557	5,599
chi-miR-330-5p_R+2	up	1.47	0.00	39	63	36	143	124	114
PC-3p-6743_861	up	1.59	0.00	7	8	14	32	28	26
pal-mir-9226-p5_1ss3 AG	up	0.94	0.00	30	25	32	50	55	60
pal-miR-9226-5p_L-1_ 1ss4AG	up	0.94	0.00	30	25	32	50	55	60
hsa-miR-200a-5p	up	inf	0.00	0	0	0	11	13	13
chi-miR-125a-3p	up	0.75	0.00	233	248	294	455	453	396
bta-miR-363_L+1	up	1.87	0.00	66	44	123	268	316	266
bta-miR-181c_R-2	up	1.79	0.00	498	397	415	1,635	1,551	1,340

bta-miR-335_R-2	up	1.81	0.00	1,653	1,111	857	4,185	4,376	4,134
bta-miR-181b_R-1	up	1.53	0.00	3,996	5,131	2,451	11,980	10,530	10,953
mmu-miR-3535_1ss20CT	down	-1.11	0.00	922	1,150	1,063	445	422	590
hsa-miR-431-3p_L+2_1ss21TG	up	inf	0.00	0	0	0	48	40	47
oar-miR-134-3p_R-1	up	3.66	0.00	0	11	0	48	45	50
bta-miR-138	up	5.93	0.00	4	0	0	71	78	90
oar-miR-154a-3p	up	1.29	0.00	83	80	48	183	166	165
bta-miR-27b	down	-1.53	0.00	92,384	86,272	77,868	28,566	31,425	28,536
bta-miR-331-3p_R+1	up	0.96	0.00	4,879	6,711	7,174	12,888	12,112	11,435
pal-miR-9226-5p_L-5R+1	up	1.30	0.00	34	36	30	88	74	88
bta-miR-10172-3p_R-1	up	2.60	0.00	7	0	7	29	28	26
bta-miR-296-3p_L-1R+1	up	1.06	0.00	983	1,164	1,185	2,590	2,209	2,169
bta-mir-2284aa-3-p3_2	up	2.29	0.00	0	8	10	32	25	30
bta-mir-2284aa-3-p3_1	up	2.29	0.00	0	8	10	32	25	30
oar-miR-370-5p	up	3.14	0.00	0	6	0	16	17	21
bta-miR-1306	up	1.22	0.00	296	426	315	803	827	785
oar-miR-758-3p_R+1	up	2.02	0.00	129	287	152	763	794	755
chi-let-7d-3p_R+1	up	0.78	0.00	5,109	5,130	3,776	8,591	8,030	7,472
bta-miR-423-3p_L-1	up	0.79	0.01	22,898	27,760	20,390	43,506	39,989	39,751
bta-miR-2285ad_L-1_1ss8AG	up	1.16	0.01	18	27	32	62	55	54
hsa-miR-615-3p_R-1	up	1.81	0.01	578	772	584	2,439	2,406	1,918
bta-miR-185	down	-0.98	0.01	4,016	4,420	4,707	2,299	2,261	2,112
oar-miR-30a-5p_R+1	down	-1.35	0.01	64,641	55,998	55,510	23,546	21,959	23,617
oar-miR-541-5p	up	2.63	0.01	13	65	25	216	218	208
oar-miR-134-5p_R+1	up	1.55	0.01	98	123	59	286	265	269
bta-miR-183_R-1	up	2.88	0.01	22	5	5	79	78	81
bta-miR-1307_R+1	up	1.20	0.01	703	1,064	1,197	2,367	2,158	2,276
chi-miR-1185-3p	up	2.29	0.01	0	11	15	41	51	35
PC-5p-6437_910	down	-1.29	0.01	60	54	46	26	18	22
hsa-miR-4454_L-3R+1	up	inf	0.01	0	0	0	7	6	5
hsa-miR-4454_1ss1GC	up	inf	0.01	0	0	0	5	6	5
bta-miR-769	up	1.10	0.01	1,397	2,195	1,582	3,687	3,507	3,883
bta-miR-450a	up	1.29	0.01	1,967	1,029	1,571	3,882	3,808	3,479
bta-miR-873_L+1R-1	up	inf	0.01	0	0	0	15	16	20
bta-miR-105a	up	inf	0.01	0	0	0	91	78	104
bta-miR-153	up	2.57	0.01	0	7	2	17	15	22
bta-miR-346	up	1.28	0.01	26	26	28	71	64	58

oar-mir-495-p5	up	2.38	0.01	19	36	0	105	95	85
bta-miR-2331-3p_R+2	up	2.15	0.01	23	0	24	63	69	81
oar-miR-665-3p_R+1	up	2.14	0.01	416	824	283	2,184	2,291	2,231
oar-miR-200c_R+1	up	2.22	0.01	306	126	118	727	1,047	796
mmu-miR-200b-5p	up	1.30	0.01	17	18	32	50	53	63
hsa-miR-432-3p	up	inf	0.01	0	0	0	44	33	38
chi-miR-128-5p_R-2	up	2.37	0.01	0	8	11	35	32	31
bta-miR-2285cs_R-1	up	inf	0.01	0	0	0	22	20	27
oan-miR-1386_L+2	up	inf	0.01	0	0	0	2	2	3
hsa-miR-450b-3p_L+1 R-1_1ss8TA	up	2.10	0.01	7	6	0	17	16	22
bta-miR-1343-5p_R+1	up	inf	0.01	0	0	0	5	4	4
PC-3p-99783_12	up	inf	0.01	0	0	0	1	2	2
oar-let-7g_R+1	down	-0.60	0.01	93,464	81,515	91,499	58,914	60,502	55,888
bta-miR-141_R+1	up	4.08	0.01	6	0	0	24	34	37
dno-miR-432-5p_L-2R +3_2	up	inf	0.01	0	0	0	9	12	11
dno-miR-432-5p_L-2R +3_1	up	inf	0.01	0	0	0	9	12	11
chi-miR-378-5p	down	-1.99	0.01	296	332	245	88	65	66
bta-miR-10172-5p	up	inf	0.01	0	0	0	5	5	4
oar-miR-485-5p	up	1.90	0.01	422	824	380	2,004	2,018	2,048
bta-miR-193a-3p_R-2	down	-2.45	0.01	593	661	801	97	129	150
chi-miR-342-5p_R-2	up	0.61	0.01	80	102	106	158	145	138
bta-miR-2448-3p	up	1.98	0.01	18	11	32	75	99	68
hsa-miR-1260b_R-1_1 ss9AG	up	0.49	0.01	2,009	1,864	1,613	2,699	2,408	2,576
chi-miR-146b-3p	up	4.12	0.01	19	17	16	353	264	286
oar-miR-382-3p_R+1	up	1.15	0.01	204	244	309	562	552	564
chi-miR-18a-3p_R-2	up	2.80	0.01	8	6	6	47	53	39
oar-miR-329b-3p_R-2	up	1.39	0.01	609	786	364	1,630	1,735	1,239
chi-miR-29a-5p	down	-3.91	0.01	47	30	35	0	7	0
bta-miR-503-5p_R+3	up	0.30	0.01	2,491	2,525	2,514	3,076	3,207	2,996
PC-5p-82157_19	up	inf	0.01	0	0	0	3	3	4
oar-miR-200b_R+1	up	1.61	0.01	1,572	940	2,183	4,668	4,925	4,704
bta-miR-3154_1ss13C T	up	inf	0.01	0	0	0	4	4	3
bta-miR-664b	down	-0.49	0.01	632	567	626	441	424	431
PC-5p-17912_273	up	3.34	0.01	0	0	37	129	124	123
hsa-miR-9983-3p	up	2.83	0.01	0	0	23	54	65	48
bta-miR-2285n_L-1R+ 2_1ss21GT	up	inf	0.01	0	0	0	3	4	3

bta-miR-2366_R+1	up	inf	0.01	0	0	0	18	14	13
oar-miR-493-5p	up	1.49	0.01	1,483	2,160	978	4,232	4,512	4,254
bta-miR-11982	up	inf	0.01	0	0	0	23	16	19
bta-mir-3431-p3	down	-0.76	0.01	387	347	322	211	217	197
bta-miR-1296	up	1.16	0.01	1,793	2,178	1,073	4,076	3,653	3,522
bta-miR-1249	down	-0.94	0.01	947	1,017	973	613	515	398
bta-miR-2285aw_L+1_1ss20TG	up	2.25	0.01	0	5	7	14	22	21
hsa-miR-4454_L-2R+1	up	inf	0.01	0	0	0	4	3	4
dno-miR-134-5p_R+1_1ss22CA	up	1.53	0.01	12	22	8	34	48	39
mdo-miR-181a-5p_R+3_2	up	1.57	0.01	32	20	21	57	81	79
mdo-miR-181a-5p_R+3_1	up	1.57	0.01	32	20	21	57	81	79
PC-5p-12827_407	down	-2.24	0.01	26	35	27	7	5	6
hsa-miR-6529-3p_1ss15TC	up	inf	0.01	0	0	0	16	19	23
oar-miR-376e-3p	up	1.00	0.01	312	322	295	565	605	692
chi-miR-127-5p_L+2R-1	up	1.42	0.01	1,389	3,438	2,399	6,341	6,138	6,924
hsa-miR-129-1-3p	up	2.86	0.01	0	0	13	32	36	28
chi-let-7e-3p	up	0.70	0.01	745	940	687	1,365	1,377	1,123
bta-mir-2285bm-p5	up	inf	0.01	0	0	0	5	7	6
oar-miR-376b-3p	up	1.82	0.01	10	20	47	85	82	104
bta-miR-424-3p	up	1.30	0.01	3,505	3,345	1,610	7,125	7,220	6,464
oar-miR-376d_1ss6AG	up	0.93	0.01	54	51	82	104	129	123
oar-miR-154b-3p_L-1	up	inf	0.01	0	0	0	10	11	7
oar-miR-329a-3p	up	inf	0.01	0	0	0	12	13	18
PC-3p-2996_2464	up	1.66	0.01	199	142	59	420	400	444
hsa-miR-221-5p	down	-3.20	0.01	450	521	649	57	46	73
efu-mir-9226-p5_1ss17GA	up	1.04	0.01	61	63	54	121	107	138
hsa-miR-7977_1ss6AG	up	0.51	0.01	193	161	209	284	253	266
bta-miR-132	down	-0.64	0.01	364	376	320	228	233	219
bta-miR-33a_R-1	down	-0.84	0.01	41	43	50	24	26	25
oar-miR-665-5p_R-2	up	1.75	0.01	28	73	19	137	150	115
oar-miR-3955-5p	up	1.08	0.01	21	33	43	72	61	71
PC-3p-22911_203	up	1.98	0.01	0	6	9	22	15	22
bta-mir-7862-p5	up	4.54	0.01	0	0	5	44	27	42
bta-miR-656	up	2.11	0.01	7	10	20	60	59	38
hsa-miR-125b-1-3p	up	0.89	0.01	330	568	443	905	808	776

bta-miR-6519_R+1_1ss17GA	up	inf	0.01	0	0	0	4	5	6
oar-miR-1197-3p	up	1.93	0.02	0	6	9	21	15	20
bta-miR-760-3p_R+2	up	0.85	0.02	126	106	173	265	243	224
oar-miR-27a_R-1	down	-1.47	0.02	19,812	14,666	16,292	5,597	6,740	5,992
oar-miR-136_R-1	up	1.77	0.02	15	38	87	143	144	191
chi-miR-450-3p_R+4	up	1.70	0.02	75	17	45	142	161	144
bta-miR-1224_1ss21GA	up	inf	0.02	0	0	0	32	22	23
bta-mir-507-p5	up	inf	0.02	0	0	0	8	7	5
bta-miR-2285x_L-1R-1_1ss22GT	up	0.95	0.02	11	11	13	20	22	26
bta-miR-378c_R+1_1ss21TA	down	-1.88	0.02	42	58	62	17	16	11
oar-miR-377-5p	up	2.31	0.02	16	15	13	82	78	57
bta-mir-2284l-p5_1ss9GT	up	2.78	0.02	0	1	0	1	2	2
bta-miR-2284aa_L-1_1ss14TC	up	2.78	0.02	0	1	0	1	2	2
bta-mir-2285cm-p5_1ss13TC	up	2.78	0.02	0	1	0	1	2	2
bta-mir-2285n-7-p5_1ss15AG	up	2.78	0.02	0	1	0	1	2	2
bta-miR-2285al-5p_R-1_1ss2CA	up	2.78	0.02	0	1	0	1	2	2
bta-miR-216b	up	3.21	0.02	6	0	0	22	18	12
hsa-miR-101-5p_L+1R-1	down	-4.19	0.02	46	30	32	3	3	0
hsa-miR-136-3p	up	1.07	0.02	429	797	804	1,472	1,446	1,328
oar-miR-369-5p	up	1.50	0.02	57	36	31	121	140	90
oar-miR-381-3p_L-1R+2_1ss5AG	up	1.14	0.02	224	377	245	616	614	633
bta-miR-2447	up	inf	0.02	0	0	0	40	30	26
oar-miR-379-3p_R-1	up	1.06	0.02	772	965	516	1,612	1,580	1,497
PC-3p-16460_302	down	-5.00	0.02	30	28	44	0	0	3
bta-miR-151-3p	down	-0.59	0.02	11,115	11,792	9,889	7,424	7,197	7,115
bta-miR-190b_R+1	up	1.14	0.02	21	35	46	79	71	76
hsa-miR-196b-3p_R+1	up	inf	0.02	0	0	0	8	7	5
bta-miR-6525_R+3_1ss2TC	up	0.59	0.02	20	21	15	29	25	29
bta-miR-9851	up	1.96	0.02	8	7	7	25	26	35
bta-miR-210_L-1	up	1.40	0.02	1,136	1,663	474	3,172	2,862	2,630
oar-miR-323b	up	inf	0.02	0	0	0	4	4	3

bta-miR-1247-5p	down	-2.34	0.02	840	1,138	785	196	181	168
bta-miR-2284y_L+1R+1	down	-0.96	0.02	38	45	32	20	16	23
oan-miR-145-5p_R+1_1ss19TA	down	-4.61	0.02	237	144	189	6	8	9
bta-miR-450b_R-1	up	0.84	0.02	1,752	1,132	1,659	2,634	2,730	2,775
bta-miR-503-3p_1ss14TC	up	1.38	0.02	103	88	27	172	184	211
oar-miR-3957-5p	up	4.08	0.02	0	0	6	22	41	35
eca-miR-508-5p_R-3	up	inf	0.02	0	0	0	17	10	13
bta-miR-130b	up	1.13	0.02	84	96	48	167	160	168
bta-mir-2887-2-p3	up	1.45	0.02	13	13	33	68	49	46
bta-miR-184	up	inf	0.02	0	0	0	8	5	6
PC-3p-36930_101	up	1.84	0.02	3	0	3	8	8	7
oar-miR-181a	up	1.23	0.02	9,661	14,041	5,240	22,927	24,400	20,746
mmu-miR-1983	up	2.89	0.02	0	7	0	18	18	18
hsa-miR-181a-3p	up	1.29	0.02	90	154	57	258	221	258
bta-mir-11986-p3_1ss17AG	up	inf	0.02	0	0	0	15	11	19
bta-miR-429	up	1.82	0.02	16	0	14	26	43	35
hsa-miR-181a-2-3p_1ss6TC	up	1.35	0.02	471	720	258	1,243	1,159	1,285
oar-miR-26b_R+1	down	-1.25	0.02	81,307	59,015	78,280	30,574	32,531	28,653
pal-miR-9226-5p_L-3	up	1.23	0.02	12	4	11	22	19	22
bta-miR-2403_R+2	up	1.86	0.02	0	9	7	25	18	15
bta-miR-330	up	0.98	0.03	341	579	325	890	785	789
bta-miR-2387_R+1	up	1.43	0.03	77	129	33	225	192	228
oar-miR-16b_R+3	up	0.44	0.03	12,482	10,432	9,831	14,792	15,352	14,158
oar-miR-26a	down	-1.25	0.03	959,980	885,070	679,463	364,289	362,968	331,011
oar-let-7a	down	-0.73	0.03	264,996	197,498	243,136	141,277	157,951	126,030
bta-miR-29d-5p_R+2	down	-1.79	0.03	578	534	381	143	140	148
oar-miR-487a-5p	up	inf	0.03	0	0	0	12	10	6
chi-miR-671-3p	up	0.94	0.03	74	100	49	156	134	136
bta-mir-2285o-3-p5_1ss12TC	up	inf	0.03	0	0	0	7	4	6
bta-mir-2284z-1-p5_1ss1AG	up	0.89	0.03	1	2	1	2	2	2
bta-mir-2284z-4-p5_1ss1AG	up	0.89	0.03	1	2	1	2	2	2
bta-mir-2284z-2-p5_1ss1AG	up	0.89	0.03	1	2	1	2	2	2
bta-mir-2285cx-p5_1ss	up	0.89	0.03	1	2	1	2	2	2

1AG									
bta-mir-2285ce-p5_1ss1AG	up	0.89	0.03	1	2	1	2	2	2
bta-miR-2285bb_1ss12GT	up	0.89	0.03	1	2	1	2	2	2
bta-mir-2904-2-p3	up	1.08	0.03	49	38	64	132	93	94
hsa-miR-16-1-3p_R+1	down	-1.67	0.03	46	32	31	13	12	9
hsa-miR-32-3p_R-1	up	1.53	0.03	9	6	6	18	25	16
hsa-miR-139-3p	up	0.80	0.03	21	35	24	45	45	50
PC-5p-63694_34	up	inf	0.03	0	0	0	5	7	4
bta-miR-877_R+2	up	1.00	0.03	40	63	83	130	126	117
hsa-miR-383-3p_L+2	up	2.52	0.03	0	5	0	9	9	11
bta-miR-449b_R+3	up	inf	0.03	0	0	0	6	8	4
oan-miR-27b-3p_R+1	down	-1.54	0.03	109	99	72	31	32	33
oar-miR-3959-5p	up	0.88	0.03	1,319	2,199	1,606	3,116	3,173	3,137
PC-3p-15354_329	down	-1.31	0.03	11	12	8	4	5	3
pal-miR-9226-5p_L-2_1ss4AG	up	0.73	0.03	39	40	61	72	75	84
pal-mir-9226-p5_1ss2AG	up	0.73	0.03	39	40	61	72	75	84
pal-miR-9226-5p_1ss4AT	up	1.87	0.03	3	3	0	6	6	10
oar-miR-379-5p_R-1	up	0.87	0.03	2,778	4,150	5,300	7,316	7,986	7,106
oar-miR-3955-3p_R+1	up	inf	0.03	0	0	0	6	6	3
hsa-miR-105-3p_L+2R-2	up	inf	0.03	0	0	0	17	9	15
chi-miR-105b-3p	up	inf	0.03	0	0	0	17	9	15
hsa-miR-505-5p_R+1	up	0.50	0.03	21	22	16	26	30	27
rno-miR-450a-5p_R-1	up	inf	0.03	0	0	0	3	5	3
bta-miR-28_R-1	down	-1.16	0.03	7,451	5,648	8,174	3,183	3,332	2,986
bta-miR-145	down	-2.01	0.03	361,549	233,552	377,870	82,509	83,016	75,430
ssc-miR-769-3p_L-1_1ss19TC	up	inf	0.03	0	0	0	7	4	5
mdo-miR-200a-3p_R+2	up	1.41	0.03	44	11	58	93	109	97
bta-miR-6529a	up	1.53	0.03	67	80	230	398	346	348
chi-miR-93-3p	up	0.73	0.03	75	71	44	96	110	108
chi-miR-3432-3p	down	-0.48	0.03	192	238	201	146	165	142
bta-miR-4286_R+1	up	0.75	0.03	67	37	52	87	75	100
oar-miR-376a-5p_L+1R-2_1ss3AG	up	inf	0.04	0	0	0	11	20	14
oar-miR-29a	down	-2.25	0.04	120,111	140,505	81,086	24,676	24,929	22,047

pal-miR-9226-5p	up	0.73	0.04	67	38	59	106	82	84
oar-miR-543-3p_R-1	up	1.08	0.04	480	710	251	1,061	1,130	844
bta-miR-1343-3p_R+2	up	0.56	0.04	924	1,371	1,126	1,798	1,611	1,646
chi-miR-1306-3p_1ss22GT	up	0.98	0.04	29	40	46	74	92	61
chi-miR-542-3p_R+2	up	1.38	0.04	2,236	2,403	5,582	8,876	8,726	9,090
PC-3p-56454_44	up	inf	0.04	0	0	0	25	18	12
bta-mir-2284aa-3-p3_1ss23TA	up	1.10	0.04	5	10	13	19	21	20
oan-miR-27b-3p_R+2	down	-1.69	0.04	133	114	80	35	30	37
hsa-miR-219a-5p_R+2	up	2.12	0.04	7	9	0	29	25	14
oar-miR-200a_L+1R+3	up	1.22	0.04	700	323	1,127	1,615	1,874	1,507
bta-miR-105b	up	inf	0.04	0	0	0	42	24	24
cpo-miR-328-3p_2ss20TC22TC	up	0.85	0.04	12	9	15	23	25	17
bta-miR-4286_R+2_2	up	0.78	0.04	30	20	19	39	38	40
bta-miR-4286_R+2_1	up	0.78	0.04	30	20	19	39	38	40
bta-miR-129-3p	down	-0.99	0.04	453	630	626	278	294	286
bta-miR-196b_R-1_1ss22GA	up	2.05	0.04	0	111	667	815	1,392	1,024
mdo-miR-181b-5p_R+3	up	2.75	0.04	0	0	5	14	6	13
bta-miR-193a-5p	down	-1.16	0.04	1,991	2,550	2,996	1,166	1,142	1,061
efu-miR-1842_R-2_1ss11GA	up	2.41	0.04	0	13	0	25	21	25
bta-miR-188	up	0.68	0.04	119	84	73	128	146	170
bta-miR-219	up	inf	0.04	0	0	0	6	7	3
bta-miR-2440_L+1R+2_1ss3GT	up	0.93	0.04	143	158	70	230	256	223
mmu-miR-135a-2-3p_R+1	up	1.81	0.04	105	229	10	425	403	380
bta-mir-502b-p5_1ss18GA	up	1.84	0.04	0	6	12	20	22	22
bta-miR-340_R-1	up	1.01	0.04	15	10	7	21	26	16
PC-5p-31506_130	up	inf	0.04	0	0	0	7	4	8
bta-miR-222_R+2	down	-2.77	0.04	4,488	2,433	4,433	520	589	551
bta-miR-101_R+1	down	-2.08	0.04	32,270	53,678	56,178	11,505	10,457	11,592
PC-5p-12815_407	up	1.67	0.04	11	15	0	30	29	26
mdo-miR-26-5p_R+1_1ss10TC	down	-1.35	0.04	91	75	57	27	28	32
bta-miR-383	up	2.12	0.04	8	204	28	361	366	318
eca-miR-483	up	inf	0.04	0	0	0	10	7	14

bta-miR-128	up	0.55	0.04	1,045	1,048	735	1,435	1,368	1,342
bta-miR-2285bj_1ss1CA	up	inf	0.04	0	0	0	1	1	2
bta-miR-2285ba_1ss11CT	up	inf	0.04	0	0	0	1	1	2
chi-miR-873-3p_R+1	up	inf	0.04	0	0	0	6	3	7
ssc-miR-7144-5p_1ss13TC	up	2.25	0.04	0	0	7	9	9	14
chi-miR-27b-5p	down	-1.29	0.04	83	68	109	32	42	32
oar-miR-411a-5p	up	0.76	0.04	2,533	4,619	4,212	6,386	6,760	6,085
cja-miR-3546_R+2	up	inf	0.04	0	0	0	4	8	4
oar-miR-30b_R+1	down	-0.89	0.05	11,786	8,708	12,520	5,722	6,395	5,730
bta-miR-16a_R+1_1ss21TA	down	-1.49	0.05	85,155	56,374	56,802	23,627	24,778	22,149
hsa-miR-4286_R+3	up	2.25	0.05	8	0	0	11	15	11
bta-mir-2904-2-p5_1ss10TC	up	3.01	0.05	6	13	14	102	53	108
bta-miR-708_1ss23GA	up	0.36	0.05	1,835	2,343	2,328	2,891	2,917	2,536
oar-miR-29b_R+1	down	-3.53	0.05	2,428	5,134	4,696	310	321	427
oar-miR-10b_L+1R-1	down	-0.37	0.05	303,014	345,952	287,889	240,554	233,733	250,791
bta-miR-2284aa_L-1R+2_1ss10TC	up	4.14	0.05	0	1	0	5	5	2
pal-miR-9226-5p_L-4	up	1.06	0.05	39	24	39	64	58	91
chi-miR-21-3p	down	-1.63	0.05	256	146	220	60	69	73
bta-miR-652_R+1	up	0.55	0.05	1,276	1,582	1,961	2,493	2,352	2,191
hsa-miR-32-5p_R-1	up	1.01	0.05	38	42	87	111	96	129

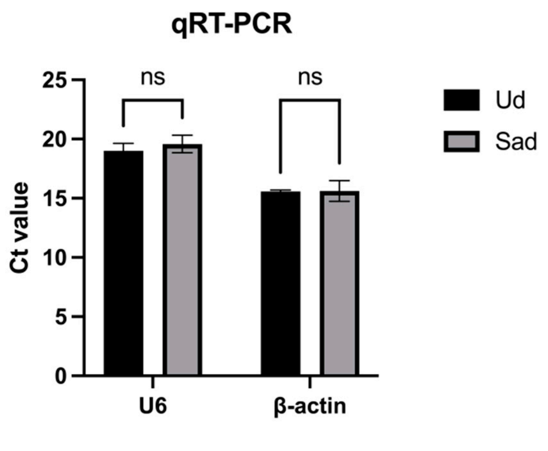

Figure. S1 the stability of reference genes

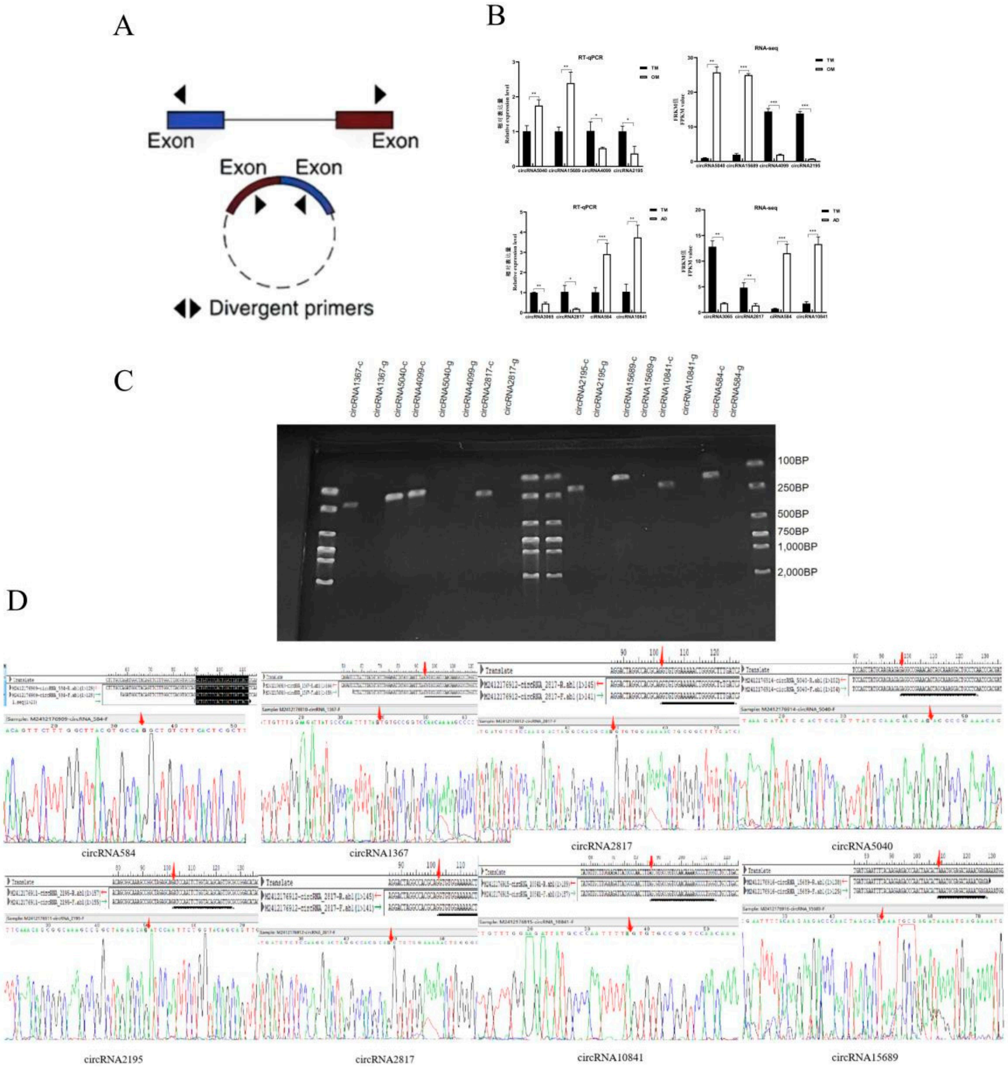

Figure. S2 Verification of DECs

A: Schematic of ring primer trans-cyclisation site design; B: RT-qPCR validation bar graph; C: Agarose gel validation graph; D: Sanger sequencing validation graph
